# Gold-catalyzed post-Ugi alkyne hydroarylation for the synthesis of 2-quinolones

**DOI:** 10.3762/bjoc.14.234

**Published:** 2018-10-04

**Authors:** Xiaochen Du, Jianjun Huang, Anton A Nechaev, Ruwei Yao, Jing Gong, Erik V Van der Eycken, Olga P Pereshivko, Vsevolod A Peshkov

**Affiliations:** 1 College of Chemistry, Chemical Engineering and Materials Science, Soochow University, Dushu Lake Campus, Suzhou, 215123, China; 2 Laboratory for Organic & Microwave-Assisted Chemistry (LOMAC), Department of Chemistry, University of Leuven (KU Leuven), Celestijnenlaan 200F, 3001 Leuven, Belgium; 3 Peoples’ Friendship University of Russia (RUDN University), 6 Miklukho-Maklaya street, Moscow, 117198, Russia; 4 Department of Chemistry, School of Science and Technology, Nazarbayev University, 53 Kabanbay Batyr Ave, Block 7, Astana, 010000, Republic of Kazakhstan

**Keywords:** gold catalysis, hydroarylation, 2-quinolones, Ugi reaction

## Abstract

A series of propargylamides containing an electron-rich benzene ring was prepared through the Ugi reaction of 3,5-dimethoxyaniline with various propiolic acids, aldehydes and isocyanides. Subjecting these adducts to a gold-catalyzed intramolecular alkyne hydroarylation process allowed to efficiently construct the 2-quinolone core bearing a branched substituent on the nitrogen atom.

## Introduction

Quinoline and its oxidized derivatives, 2-quinolone and 4-quinolone, are the core structural elements of many natural products and pharmaceutical agents [1–3]. In particular, 2-quinolone derivatives show a broad range of biological activities including antiviral [4–7], antimicrobial [8–9], antiparasitic [10–11], anti-inflammatory [12–13] and anticancer [9,13–19]. In addition, 2-quinolones were identified as promising entities for the treatment of neuropathic pain [20–22] and erectile dysfunction [23]. Therefore, the elaboration of practical methodologies for the synthesis [24] and functionalization [25–29] of the 2-quinolone scaffold has become a budding research trend. In the last decade, a great number of efficient approaches has been developed utilizing transition metal-catalyzed [30–32], Lewis acid-mediated [33], and radical cyclizations [34] of various aniline derivatives with most recent strategies focusing on the implementation of transition metal-catalyzed C–H activation methods [35–36].

**Scheme 1:**
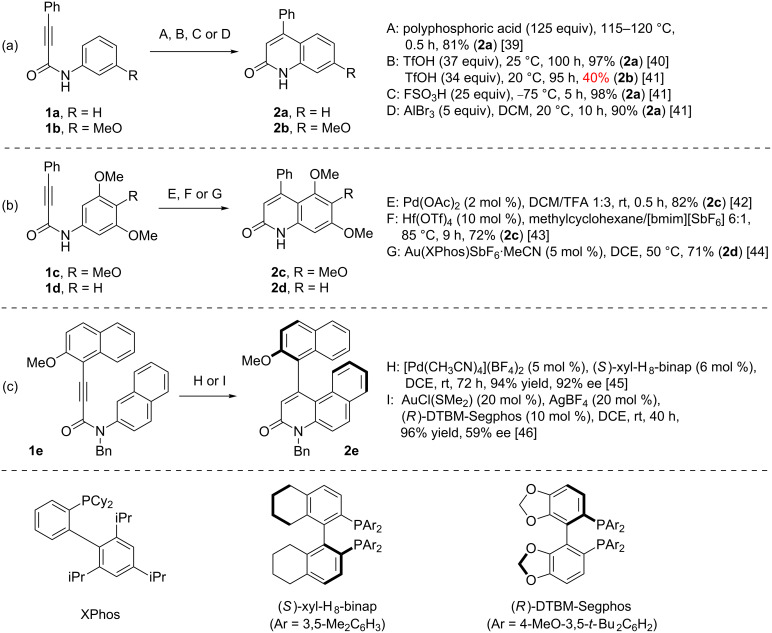
Synthesis of 2-quinolones **2** through intramolecular Friedel–Crafts hydroarylation of *N*-aryl propargylamides **1**.

One of the common approaches towards 2-quinolones **2** involves the intramolecular Friedel–Crafts hydroarylation [37–38] of *N*-arylamides of 3-substituted propynoic acids **1** (Scheme 1). It was found that the use of superstoichiometric amounts of strong Brønsted [39–41] or Lewis [40–41] acids give good results on substrates with non-activated *N*-aryl groups such as **1a**, while substrate **1b** bearing an additional electron-donating group on the *N*-aryl fragment gave a poorer outcome under similar settings (Scheme 1a). Consequently, several catalytic methods were developed to extend the scope of this Friedel–Crafts process to substrates such as **1c** and **1d** featuring highly electron-rich *N*-aryl moieties (Scheme 1b) [42–44]. Finally, following the success of the above procedures, two asymmetric versions were designed to provide access to axially chiral 2-quinolone-based heterobiaryls such as **2e** (Scheme 1c) [45–46].

**Scheme 2:**
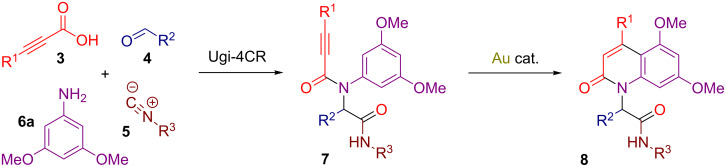
Strategy towards 2-quinolones **8** bearing a branched substituent on the nitrogen atom.

One substantial drawbacks of the previous methodologies is that they do lack an exploration of the substituent diversity on the nitrogen atom. We decided to address this issue by employing propargylamides **7** obtained by a four-component Ugi reaction of propiolic acids **3**, aldehydes **4**, isocyanides **5** and 3,5-dimethoxyaniline (**6a**) (Scheme 2). We anticipated that the resulting adducts **7** would readily produce 2-quinolones **8** bearing a branched substituent on the nitrogen atom through an intramolecular gold-catalyzed alkyne hydroarylation reaction (Scheme 2). It should be noted, that heterocyclic syntheses through post-Ugi transformations have been under extensive exploration for the last two decades [47–49], especially in terms of gold-catalyzed Friedel–Crafts-type cyclizations involving attack of electron-rich arenes on the triple bonds, leading to the formation of a great number of fused [50–53] and spirocyclic structures [54–59]. In addition, Ugi adducts have already been successfully utilized for the diversity-oriented synthesis of 2-quinolones using either intramolecular Heck reaction [60] or Knoevenagel condensation [61–62].

## Results and Discussion

We began our study with the preparation of the model substrate **7a** through the Ugi reaction of tetrolic acid (**3a**), benzaldehyde (**4a**), *tert*-butyl isocyanide (**5a**) and 3,5-dimethoxyaniline (**6a**). Next, the cycloisomerization of **7a** was investigated in order to identify the optimal conditions. At first, we attempted two reactions using 5 mol % of the standard AuPPh_3_Cl/AgOTf precatalytic combination in conventional chlorinated solvents such as deuterated chloroform and dichloromethane (Table 1, entries 1 and 2). The latter proved to be a better choice affording the targeted 2-quinolone **8a** in up to 90% yield (Tabel 1, entry 2). Using AgOTf in the absence of gold slowed down the reaction leading to decreased yields of **8a** even at an elevated temperature of 60 °C (Table 1, entries 3–5). Conducting the AuPPh_3_Cl/AgOTf-catalyzed reaction in trifluoroethanol (TFE) led to improved results producing **8a** in up to 97% yield (Table 1, entry 6). Thus, this greener alternative [63] to chlorinated solvents was selected as the solvent of choice for the further exploration. Changing the silver counterpart to AgPF_6_ did not affect the reaction outcome yielding **8a** in 96% (Table 1, entry 7). Using AgOTf or AuPPh_3_Cl as sole catalyst did not provide satisfactory results (Table 1, entries 8–10). In particular, the AgOTf-catalyzed reaction conducted at 60 °C produced substantial amounts of trifluoroethanol adduct **9a** (Table 1, entry 8), while the AuPPh_3_Cl-catalyzed reaction suffered from a slow conversion rate of starting **7a** (Table 1, entries 9 and 10).

**Table 1:** Screening of the conditions for the intramolecular hydroarylation of the Ugi adduct **7a**.^a^

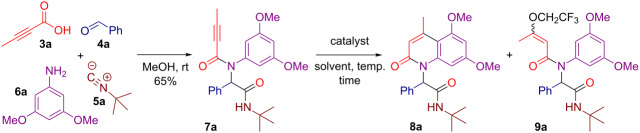							

Entry	Catalyst (x mol %)	Solvent	Temp., °C	Time, h	Conversion of **7a**, %^b^	Yield of **8a**, %^b^	Yield of **9a**, %^b^

1	AuPPh_3_Cl/AgOTf (5)	CDCl_3_	rt	12	94	84	–
2	AuPPh_3_Cl/AgOTf (5)	DCM	rt	12	100	90	–
3	AgOTf (5)	CDCl_3_	rt	12	40	30	–
4	AgOTf (5)	CDCl_3_	60 °C	4	70	58	–
5	AgOTf (5 + 5)^c^	CDCl_3_	60 °C	6	90	74	–
6	AuPPh_3_Cl/AgOTf (5)	CF_3_CH_2_OH	rt	12	100	97 (96)^d^	trace
7	AuPPh_3_Cl/AgPF_6_ (5)	CF_3_CH_2_OH	rt	12	100	96	–
8	AgOTf (5 + 5)^c^	CF_3_CH_2_OH	60 °C	6	100	86	11
9	AuPPh_3_Cl (5)	CF_3_CH_2_OH	rt	12	13	12	–
10	AuPPh_3_Cl (5)	CF_3_CH_2_OH	60 °C	12	79	76	–

^a^Reaction conditions: **7a** (0.2 mmol), solvent (2 mL), under air. ^b^Determined by ^1^H NMR using 3,4,5-trimethoxybenzaldehyde as an internal standard. ^c^The first portion of catalyst was added at the beginning of reaction, while the second portion was added after 3 h. ^d^Isolated yield for a 0.5 mmol scale reaction is given in parenthesis.

**Figure 1:**
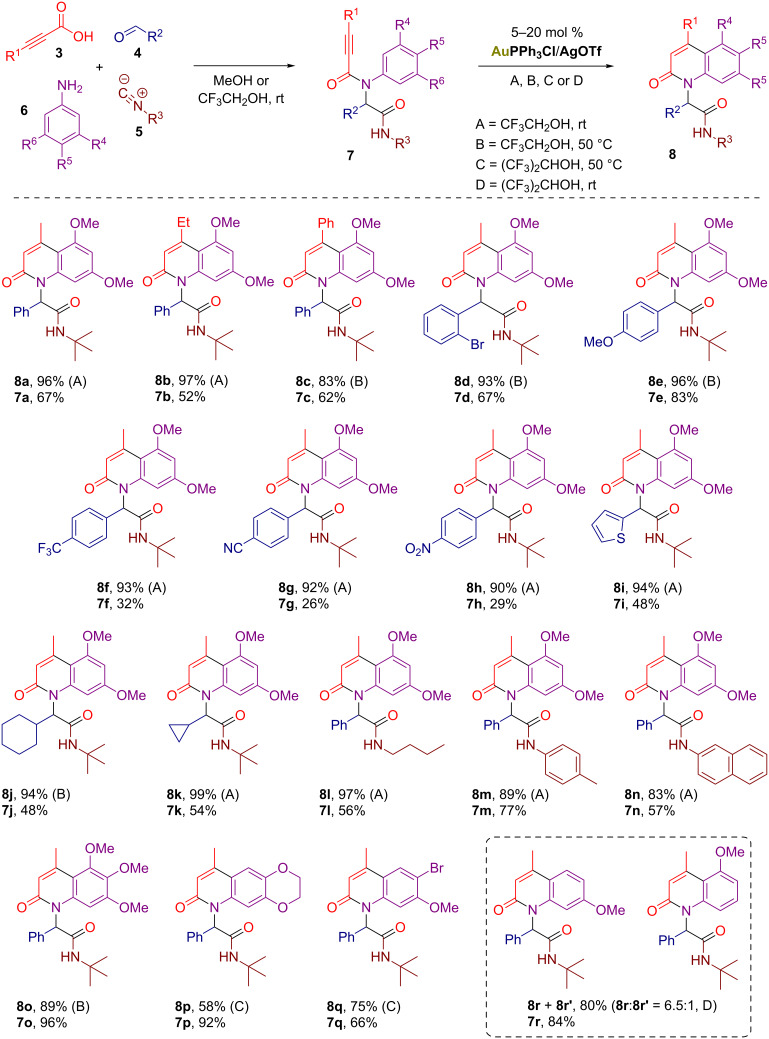
Scope of the protocol.

Next, we attempted to delineate the scope of our methodology (Figure 1). Exploring different 3-substituted propiolic acids **3** in combination with **4a**, **5a** and **6a** gave moderate results for the Ugi reaction and good to excellent efficiency for the gold-catalyzed cyclization (Figure 1, products **8a–c**). As for the aldehyde component **4**, both (hetero)aromatic and aliphatic ones were found to be tolerable (Figure 1, products **8d–k**), although for the electron-deficient aromatic aldehydes only low yields were achieved during the Ugi step. Yet, the subsequent gold-catalyzed cyclization worked well delivering the 2-quinolones **8f–h** in up to 93% yield. Besides, several aliphatic and aromatic isocyanates **5** have been successfully employed for the preparation of 2-quinolones **8l–n**. Finally, we decided to investigate the reactions of Ugi adducts **7o–r** derived from various electron-rich anilines **6**. The gold-catalyzed cyclization of 3,4,5-trimethoxyaniline-derived substrate **7o** proceeded at the elevated temperature of 50 °C producing 2-quinolone **8o** in 89% yield. The cyclizations of **7p–r** required further optimization of the reaction conditions (Tables 2–4) and were complicated due the formation of regioisomeric products. After all, 2-quinolones **8p** and **8q** were isolated in good yields from gold-catalyzed reactions in hexafluoroisopropanol (HFIP), while 2-quinolone **8r** was obtained as a mixture with isomer **8r’**.

As noted above, the intramolecular alkyne hydroarylations with certain Ugi adducts required further adjustment of the reaction conditions. For example, the reaction of substrate **7p** being conducted in TFE at room temperature delivered a complex mixture of regioisomeric 2-quinolones **8p/8p’** and TFE-adduct **9b** (Table 2, entry 1). However, the application of branched fluorinated alcohols as solvent solved the problem of the competing alkoxylation reaction. Thus, no alcohol adducts were formed when the gold-catalyzed cyclization of **7p** was conducted in hexafluoro-2-methylpropan-2-ol or HFIP (Table 2, entries 2–4). Using the latter solvent a full conversion of **7p** was achieved at 50 °C within 20 h, allowing to isolate both possible products **8p** and **8p’** in 58% and 15% yields, respectively (Table 2, entry 4). It should also be stressed, that reacting **7p** in chloroform at rt failed to produce any traces of cyclized products (Table 2, entry 5).

**Table 2:** Screening the conditions for the gold-catalyzed intramolecular hydroarylation of the Ugi adduct **7p**.^a^

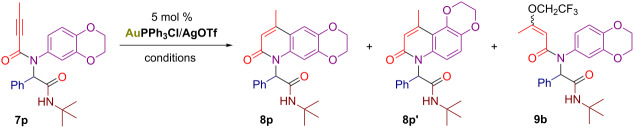					

Entry	Conditions	Conversion of **7p**, %^b^	Yield of **8p**, %^b^	Yield of **8p’**, %^b^	Yield of **9b**, %^b^

1	CF_3_CH_2_OH, rt	79	30	7	14
2	CH_3_(CF_3_)_2_COH, rt	34	27	6	–
3	CH_3_(CF_3_)_2_COH, 50 °C	87	66 (48)^c^	14	–
4^d^	(CF_3_)_2_CHOH, 50 °C	100	71 (58)^c^	20 (15)^c^	–
5	CHCl_3_, rt	10	–	–	–

^a^Reaction conditions: **7p** (0.2 mmol), solvent (2 mL), 12 h, under air. ^b^Determined by ^1^H NMR using 3,4,5-trimethoxybenzaldehyde as an internal standard. ^c^Isolated yields for 0.3 mmol scale reactions are given in parentheses. ^d^The reaction was conducted for 20 h.

A 4-bromo-3-methoxyaniline-derived Ugi adduct **7q** proved to be the most difficult substrate. The initial attempts to perform an intramolecular alkyne hydroarylation of **7q** in TFE were characterized by a rather slow reaction rate which concomitantly promoted the competing alkoxylation reaction (Table 3, entries 1 and 2). Consequently, we were able to isolate and characterize the corresponding TFE-adduct **9c** (Table 3, entry 2). Switching to HFIP as the solvent prevented the alkoxylation but led to an even slower reaction rate (Table 3, entries 3–5). Increasing the catalyst loading to 10 and finally up to 20 mol % allowed to achieve a full conversion of **7q** within 30 h producing 2-quinolone **8q** in 75% isolated yield (Table 3, entries 5 and 6).

**Table 3:** Screening the conditions for the gold-catalyzed intramolecular hydroarylation of the Ugi adduct **7q**.^a^

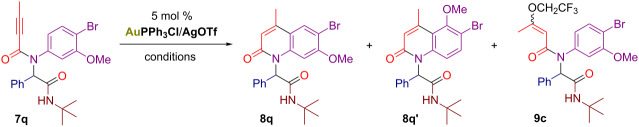					

Entry	Conditions	Conversion of **7q**, %^b^	Yield of **8q**, %^b^	Yield of **8q’**, %^b^	Yield of **9c**, %^b^

1	CF_3_CH_2_OH, rt	28	4	1	11
2	CF_3_CH_2_OH, 50 °C	58	8	2	22 (18)^c^
3	(CF_3_)_2_CHOH, rt	12	7	1	-
4	(CF_3_)_2_CHOH, 50 °C	24	14	4	-
5^d^	(CF_3_)_2_CHOH, 50 °C	51	40	6	-
6^e^	(CF_3_)_2_CHOH, 50 °C	100	76 (75)^c^	18	-

^a^Reaction conditions: **7q** (0.2 mmol), AuPPh_3_Cl/AgOTf (5 mol %), solvent (2 mL), 12 h, under air. ^b^Determined by ^1^H NMR using 3,4,5-trimethoxybenzaldehyde as an internal standard. ^c^Isolated yields for 0.4 mmol scale reactions are given in parentheses. ^d^The reaction was conducted for 24 h using 10 mol % of AuPPh_3_Cl/AgOTf catalyst. ^e^The reaction was conducted for 30 h using 20 mol % of AuPPh_3_Cl/AgOTf catalyst.

Analogously to **7p** and **7q**, the cyclization of 3-methoxyaniline-derived substrate **7r** being conducted in TFE did not lead to a full conversion within 12 h at rt (Table 4, entry 1). Switching to branched fluorinated solvents led to a faster conversion of **7r** simultaneously suppressing the competing alkoxylation (Table 4, entries 2–4). Nonetheless, the transformation of **7r** was further complicated by the fact that the resulting cyclized products **8r** and **8r’** were essentially inseparable. The best **8r**/**8r’** ratio could be obtained using hexafluoro-2-methylpropan-2-ol while the best overall yield was obtained using HFIP (Table 4, entry 2 versus entry 4).

**Table 4:** Screening the conditions for the gold-catalyzed intramolecular hydroarylation of the Ugi adduct **7r**.^a^

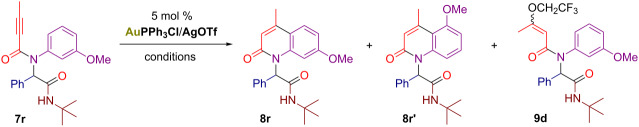				

Entry	Conditions	Conversion of **7r**, %^b^	Combined yield of **8r** and **8r’** (**8r**:**8r’**), %^b^	Yield of **9d**, %^b^

1	CF_3_CH_2_OH, rt, 12 h	70	48 (3.9:1)	10
2	CH_3_(CF_3_)_2_COH, rt, 12 h	100	85 (3.9:1)	–
3	CH_3_(CF_3_)_2_COH, 50 °C, 4 h	100	84 (3.4:1)	–
4	(CF_3_)_2_CHOH, rt, 12 h	100	94 (3.5:1); 80^c^ (6.5:1)	–

^a^Reaction conditions: **7r** (0.15 mmol), solvent (1.5 mL), under air. ^b^Determined by ^1^H NMR using 3,4,5-trimethoxybenzaldehyde as an internal standard. ^c^Combined yield for a 0.4 mmol scale reaction obtained after column chromatography.

## Conclusion

We have elaborated a fast and diversity-oriented approach towards 2-quinolones bearing a branched substituent on the nitrogen atom. The strategy relies on the application of a four-component Ugi reaction followed by a gold-catalyzed intramolecular alkyne hydroarylation. The developed process has a broad scope and simple reaction settings. Another important highlight of the developed process is that it strongly benefits from the employment of fluorinated alcohols as environmentally benign solvents.

## Supporting Information

File 1 Full experimental procedures and spectroscopic characterizations, as well as the copies of ^1^H and ^13^C NMR spectra of Ugi products **7** and final 2-quinolones **8**.
