# Supplementary material for: Gold-catalyzed post-Ugi alkyne hydroarylation for the synthesis of 2-quinolones
Source: Beilstein J Org Chem. 2018 Oct 4;14:2572–9. doi: 10.3762/bjoc.14.234 (PMC6204774; doi:10.3762/bjoc.14.234)
Supplement: File 1 — Full experimental procedures and spectroscopic characterizations, as well as the copies of 1H and 13C NMR spectra of Ugi products 7 and final 2-quinolones 8. [file Beilstein_J_Org_Chem-14-2572-s001.pdf]

Supporting Information

for

Gold-catalyzed post-Ugi alkyne hydroarylation for the synthesis of 2-quinolones

Xiaochen Du[1,§], Jianjun Huang[1,§], Anton A. Nechaev[2], Ruwei Yao[1], Jing Gong[1], Erik V. Van der Eycken[2,3], Olga P. Pereshivko[1]*, Vsevolod A. Peshkov[1,4,$]*

Address: [1]College of Chemistry, Chemical Engineering and Materials Science, Soochow University, Dushu Lake Campus, Suzhou, 215123, China, [2]Laboratory for Organic & Microwave-Assisted Chemistry (LOMAC), Department of Chemistry, University of Leuven (KU Leuven), Celestijnenlaan 200F, 3001 Leuven, Belgium, [3]Peoples' Friendship University of Russia (RUDN University), 6 Miklukho-Maklaya street, Moscow, 117198, Russia and [4]Department of Chemistry, School of Science and Technology, Nazarbayev University, 53 Kabanbay Batyr Ave, Block 7, Astana, 010000, Republic of Kazakhstan

Email: Olga P. Pereshivko - olga@suda.edu.cn; Vsevolod A. Peshkov - vsevolod@suda.edu.cn;

*Corresponding author

[§]Equally contributing authors

[$]Alternative email address: vsevolod.peshkov@nu.edu.kz

Full experimental procedures and spectroscopic characterizations, as well as the copies of ^{1}H and ^{13}C NMR spectra of Ugi products **7** and final 2-quinolones **8**

Table of contents

General remarks	S2
Synthesis of starting materials	S2
General procedure for Ugi reaction	S2
General procedure for the gold-catalyzed synthesis of 2-quinolones 8	S8
Copies of ^{1}H and ^{13}C NMR spectra	S15

General remarks

Unless otherwise specified, the starting materials and solvents were purchased from commercial sources and used as received. Melting points were measured using INESA WRR apparatus. Infrared (FTIR) spectra were recorded neat on a Bruker Vertex 70. ^{1}H NMR (400 MHz) and ^{13}C NMR (100 MHz) spectra were recorded using a Bruker Avance III HD instrument. The ^{1}H NMR and ^{13}C NMR chemical shifts are reported relative to TMS using the residual $CDCl_3$ signal as internal reference. HRMS were performed on a Bruker micrOTF-Q III.

Synthesis of starting materials

1-Isocyano-4-methylbenzene (**5c**) was synthesized following the reported procedure [1].

General procedure for the Ugi reaction

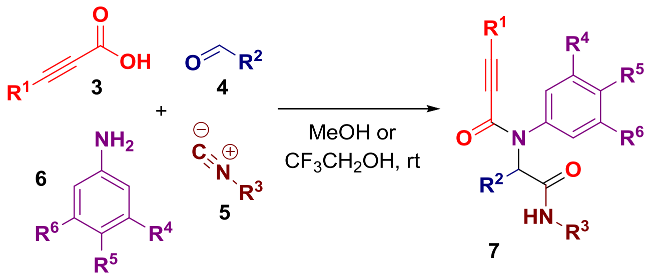

Aniline **6** (1.5 mmol) and 3-subsituted propiolic acid **3** (1.5 mmol) were dissolved in MeOH (7.5 mL) followed by the addition of aldehyde **4** (1.5 mmol) and isocyanide **5** (1.5 mmol). The reaction mixture was sealed and stirred at room temperature for 24 h. The resulting mixture was diluted with EtOAc and concentrated with silica. Column chromatography with petroleum ether/EtOAc or petroleum ether/DCM (the ratio was adjusted according to TLC) as eluent delivered Ugi adduct **7**. For the synthesis of Ugi adducts **7c** and **7d**, CF_3CH_2OH was used as the solvent instead of MeOH. For the synthesis of Ugi adducts **7m** and **7n**, the order of addition was as follows: isocyanide **5** (1.5 mmol) was dissolved in MeOH (7.5 mL) followed by the addition of aniline **6** (1.5 mmol), 3-subsituted propiolic acid **3** (1.5 mmol) and aldehyde **4** (1.5 mmol).

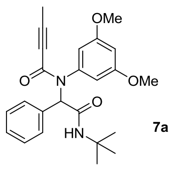

7a

***N*-(2-(*tert*-Butylamino)-2-oxo-1-phenylethyl)-*N*-(3,5-dimethoxyphenyl)but-2-ynamide (7a).** Yield: 67%; yellow solid; mp 188-189°C; ^{1}H NMR (400 MHz, $CDCl_3$): δ 7.26-7.14 (m, 5H), 6.32 (s, 3H), 5.85 (s, 1H), 5.65 (bs, 1H), 3.64 (s, 6H), 1.74 (s, 3H), 1.33 (s, 9H); ^{13}C NMR (100 MHz, $CDCl_3$): δ

[1] Leifert, D.; Artiukhin, D. G.; Neugebauer, J.; Galstyan, A.; Strassert, C. A.; Studer, A. *Chem. Commun.* **2016**, *52*, 5997−6000 (Supporting Information).

168.0, 160.3, 154.9, 141.5, 134.5, 130.3, 128.6, 128.5, 108.7, 101.1, 91.1, 74.1, 65.9, 55.6, 51.8, 28.7, 4.2; HRMS (ESI, $[M+Na]^+$) for $C_{24}H_{28}N_2O_4Na^+$ calcd 431.1941; found 431.1975.

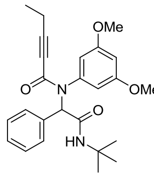

7b

***N*-(2-(*tert*-Butylamino)-2-oxo-1-phenylethyl)-*N*-(3,5-dimethoxyphenyl)pent-2-ynamide (7b).** Yield: 52%; yellow solid; mp 183-184°C; ^{1}H NMR (400 MHz, $CDCl_3$): δ 7.26-7.16 (m, 5H), 6.38-6.29 (m, 3H), 5.87 (s, 1H), 5.65 (bs, 1H), 3.64 (s, 6H), 2.08 (q, *J* = 7.5 Hz, 2H), 1.34 (s, 9H), 0.87 (t, *J* = 7.3 Hz, 3H); ^{13}C NMR (100 MHz, $CDCl_3$): δ 168.0, 160.3, 155.0, 141.7, 134.5, 130.3, 128.6, 128.5, 108.8, 101.1, 96.2, 74.4, 65.8, 55.6, 51.8, 28.7, 12.64, 12.62; HRMS (ESI, $[M+H]^+$) for $C_{25}H_{31}N_2O_4^+$ calcd 423.2278; found 423.2294.

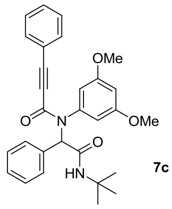

7c

***N*-(2-(*tert*-Butylamino)-2-oxo-1-phenylethyl)-*N*-(3,5-dimethoxyphenyl)-3-phenylpropiolamide (7c).** Yield: 62%; yellow solid; mp 93-94°C; ^{1}H NMR (400 MHz, $CDCl_3$): δ 7.38-7.19 (m, 8H), 7.17-7.09 (m, 2H), 6.48-6.34 (m, 3H), 5.93 (s, 1H), 5.65 (bs, 1H), 3.65 (s, 6H), 1.36 (s, 9H); ^{13}C NMR (100 MHz, $CDCl_3$): δ 167.9, 160.4, 155.0, 141.5, 134.4, 132.7, 130.4, 130.1, 128.7, 128.6, 128.4, 120.6, 108.9, 101.4, 91.9, 82.6, 65.9, 55.6, 51.8, 28.7; HRMS (ESI, $[M+H]^+$) for $C_{29}H_{31}N_2O_4^+$ calcd 471.2278; found 471.2289.

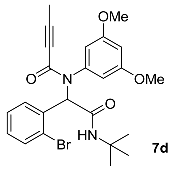

7d

***N*-(1-(2-Bromophenyl)-2-(*tert*-butylamino)-2-oxoethyl)-*N*-(3,5-dimethoxyphenyl)but-2-ynamide (7d).** Yield: 67%; white solid; mp 159-160°C; ^{1}H NMR (400 MHz, $CDCl_3$): δ 7.53-7.46 (m, 1H), 7.26-7.18 (m, 1H), 7.10-7.01 (m, 2H), 6.50 (bs, 2H), 6.25 (t, *J* = 2.1 Hz, 1H), 6.21 (s, 1H), 5.55 (s, 1H), 3.65 (s, 6H), 1.74 (s, 3H), 1.36 (s, 9H); ^{13}C NMR (100 MHz, $CDCl_3$) δ 167.8, 160.2, 154.8, 140.7, 133.9, 132.8, 132.1, 130.2, 127.4, 126.4, 108.5, 101.3, 90.9, 74.0, 64.3, 55.6, 52.0, 28.7, 4.2; HRMS (ESI, $[M+H]^+$) for $C_{24}H_{28}BrN_2O_4^+$ calcd 487.1227; found 487.1236.

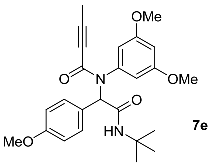

7e

***N*-(2-(*tert*-Butylamino)-1-(4-methoxyphenyl)-2-oxoethyl)-*N*-(3,5-dimethoxyphenyl)but-2-ynamide (7e).** Yield: 83%; white solid; mp 163-164°C; ^{1}H NMR (400 MHz, $CDCl_3$): δ 7.09 (d, *J* = 8.5 Hz, 2H), 6.74 (d, *J* = 8.7 Hz, 2H), 6.37-6.27 (m, 3H), 5.82 (s, 1H), 5.63 (bs, 1H), 3.75 (s, 3H), 3.66 (s, 6H), 1.73 (s, 3H), 1.33 (s, 9H); ^{13}C NMR (100 MHz, $CDCl_3$): δ 168.3, 160.3, 159.7, 154.9, 141.5, 131.7, 126.4, 113.8, 108.8, 101.1, 90.9, 74.2, 65.1, 55.6, 55.4, 51.7, 28.7, 4.2; HRMS (ESI, $[M+H]^+$) for $C_{25}H_{31}N_2O_5^+$ calcd 439.2227; found 439.2239.

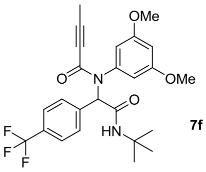

7f

***N*-(2-(*tert*-Butylamino)-2-oxo-1-(4-(trifluoromethyl)phenyl)ethyl)-*N*-(3,5-dimethoxyphenyl)but-2-ynamide (7f).** Yield: 32%; white solid; mp 199-200°C; ^{1}H NMR (400 MHz, $CDCl_3$): δ 7.49 (d, *J* = 8.2 Hz, 2H), 7.35 (d, *J* = 8.1 Hz, 2H), 6.35 (t, *J* = 2.2 Hz, 1H), 6.27 (bs, 2H), 5.95-5.85 (m, 2H), 3.66 (s, 6H), 1.76 (bs, 3H), 1.36 (s, 9H); ^{13}C NMR (100 MHz, $CDCl_3$): δ 167.4, 160.6, 155.0, 141.0, 138.3, 130.63, 130.61 (q, *J* = 32.4 Hz), 125.26 (q, *J* = 3.7 Hz), 124.0 (q, *J* = 272.2 Hz), 108.5, 101.2, 91.8, 73.8, 65.2, 55.6, 51.9, 28.7, 4.2; HRMS (ESI, $[M+H]^+$) for $C_{25}H_{28}F_3N_2O_4^+$ calcd 477.1996; found 477.2009.

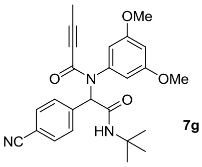

7g

***N*-(2-(*tert*-Butylamino)-1-(4-cyanophenyl)-2-oxoethyl)-*N*-(3,5-dimethoxyphenyl)but-2-ynamide (7g).** Yield: 26%; yellow solid; mp 240-241°C; ^{1}H NMR (400 MHz, $CDCl_3$): δ 7.52 (d, *J* = 8.3 Hz, 2H), 7.36 (d, *J* = 8.2 Hz, 2H), 6.36 (t, *J* = 2.0 Hz, 1H), 6.30-6.25 (m, 2H), 5.98 (bs, 1H), 5.88 (s, 1H), 3.68 (s, 6H), 1.77 (bs, 3H), 1.36 (s, 9H); ^{13}C NMR (100 MHz, $CDCl_3$) δ 167.2, 160.7, 155.0, 140.9, 139.5, 132.0, 130.9, 118.5, 112.4, 108.4, 101.0, 92.2, 73.7, 65.1, 55.6, 52.0, 28.7, 4.2; HRMS (ESI, $[M+H]^+$) for $C_{25}H_{28}N_3O_4^+$ calcd 434.2074; found 434.2084.

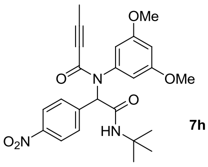

7h

***N*-(2-(*tert*-Butylamino)-1-(4-nitrophenyl)-2-oxoethyl)-*N*-(3,5-dimethoxyphenyl)but-2-ynamide (7h).** Yield: 29%; white solid; mp 197-198°C; ^{1}H NMR (400 MHz, $CDCl_3$): δ 8.08 (d, *J* = 8.8 Hz, 2H),

7.43 (d, J = 8.7 Hz, 2H), 6.36 (t, J = 2.1 Hz, 1H), 6.32-6.27 (m, 2H), 6.03 (bs, 1H), 5.92 (s, 1H), 3.69 (s, 6H), 1.77 (bs, 3H), 1.37 (s, 9H); ^{13}C NMR (100 MHz, $CDCl_3$): δ 167.1, 160.7, 155.1, 147.8, 141.4, 140.9, 131.2, 123.4, 108.4, 101.0, 92.4, 73.7, 64.9, 55.6, 52.1, 28.7, 4.2; HRMS (ESI, $[M+H]^+$) for $C_{24}H_{28}N_3O_6^+$ calcd 454.1973; found 454.1992.

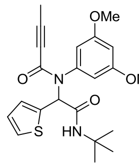

7i

***N*-(2-(*tert*-Butylamino)-2-oxo-1-(thiophen-2-yl)ethyl)-*N*-(3,5-dimethoxyphenyl)but-2-ynamide (7i).** Yield: 48%; yellow solid; mp 161-162°C; ^{1}H NMR (400 MHz, $CDCl_3$): δ 7.27-7.23 (m, 1H), 7.04-6.97 (m, 1H), 6.90 (dd, J = 5.1, 3.6 Hz, 1H), 6.38 (t, J = 2.2 Hz, 1H), 6.29 (d, J = 2.0 Hz, 2H), 6.14 (s, 1H), 6.09 (bs, 1H), 3.68 (s, 6H), 1.75 (s, 3H), 1.36 (s, 9H); ^{13}C NMR (100 MHz, $CDCl_3$): δ 167.1, 160.5, 154.6, 141.1, 135.7, 129.8, 128.1, 126.2, 108.1, 101.4, 91.6, 73.9, 60.5, 55.6, 51.8, 28.7, 4.2; HRMS (ESI, $[M+H]^+$) for $C_{22}H_{27}N_2O_4S^+$ calcd 415.1686; found 415.1695.

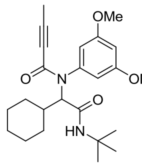

7j

***N*-(2-(*tert*-Butylamino)-1-cyclohexyl-2-oxoethyl)-*N*-(3,5-dimethoxyphenyl)but-2-ynamide (7j).** Yield: 48%; yellow solid; mp 122-123°C; ^{1}H NMR (400 MHz, $CDCl_3$): δ 6.82 (bs, 1H), 6.47 (d, J = 2.1 Hz, 2H), 6.43 (t, J = 2.2 Hz, 1H), 4.12 (d, J = 9.8 Hz, 1H), 3.78 (s, 6H), 2.20-2.06 (m, 1H), 1.94-1.85 (m, 1H), 1.82-1.58 (m, 7H), 1.35 (s, 9H), 1.31-1.07 (m, 3H), 1.02-1.84 (m, 2H); ^{13}C NMR (100 MHz, $CDCl_3$): δ 169.1, 160.6, 155.7, 142.7 (bs), 107.3, 100.7, 91.6, 74.3, 70.5 (bs), 55.6, 51.2, 36.0, 30.4, 30.0, 28.8, 26.5, 25.7, 25.6, 4.2; HRMS (ESI, $[M+H]^+$) for $C_{24}H_{35}N_2O_4^+$ calcd 415.2591; found 415.2599.

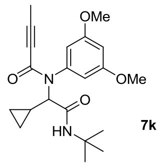

7k

***N*-(2-(*tert*-Butylamino)-1-cyclopropyl-2-oxoethyl)-*N*-(3,5-dimethoxyphenyl)but-2-ynamide (7k).** Yield: 54%; white solid; mp 163-164°C; ^{1}H NMR (400 MHz, $CDCl_3$): δ 6.52 (d, J = 2.0 Hz, 2H), 6.46 (t, J = 1.9 Hz, 1H), 6.35 (bs, 1H), 4.01 (d, J = 10.6 Hz, 1H), 3.78 (s, 6H), 1.77 (s, 3H), 1.38 (s, 9H), 1.10-0.96 (m, 1H), 0.76-0.65 (m, 1H), 0.55-0.44 (m, 1H), 0.43-0.27 (m, 2H); ^{13}C NMR (100 MHz, $CDCl_3$): δ 168.9, 160.6, 155.2, 141.0, 108.1, 101.1, 91.6, 73.9, 65.6, 55.6, 51.4, 28.8, 10.4, 5.9, 4.2; HRMS (ESI, $[M+H]^+$) for $C_{21}H_{29}N_2O_4^+$ calcd 373.2122; found 373.2134.

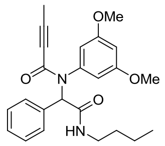

7l

***N*-(2-(Butylamino)-2-oxo-1-phenylethyl)-*N*-(3,5-dimethoxyphenyl)but-2-ynamide (7l).** Yield: 56%; white solid; mp 125-126°C; ^{1}H NMR (400 MHz, $CDCl_3$): δ 7.27-7.16 (m, 5H), 6.36 (bs, 2H), 6.33 (t, *J* = 2.2 Hz, 1H), 5.89 (s, 1H), 5.75 (bt, *J* = 5.3 Hz, 1H), 3.66 (s, 6H), 3.32-3.24 (m, 2H), 1.74 (s, 3H), 1.51-1.40 (m, 2H), 1.35-1.22 (m, 2H), 0.88 (t, *J* = 7.3 Hz, 3H); ^{13}C NMR (100 MHz, $CDCl_3$): δ 168.9, 160.3, 154.9, 141.6, 134.3, 130.2, 128.7, 128.6, 108.6, 101.2, 91.1, 74.1, 65.7, 55.6, 39.8, 31.6, 20.1, 13.8, 4.2; HRMS (ESI, $[M+H]^+$) for $C_{24}H_{29}N_2O_4^+$ calcd 409.2122; found 409.2132.

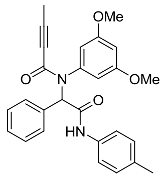

7m

***N*-(3,5-Dimethoxyphenyl)-*N*-(2-oxo-1-phenyl-2-(*p*-tolylamino)ethyl)but-2-ynamide (7m).** Yield: 77%; yellow solid; mp 202-203°C; ^{1}H NMR (400 MHz, $CDCl_3$): δ 7.67 (bs, 1H), 7.36 (d, *J* = 8.3 Hz, 2H), 7.30-7.24 (m, 5H), 7.10 (d, *J* = 8.3 Hz, 2H), 6.40-6.31 (m, 3H), 6.10 (s, 1H), 3.65 (s, 6H), 2.30 (s, 3H), 1.76 (s, 3H); ^{13}C NMR (100 MHz, $CDCl_3$): δ 167.2, 160.4, 155.2, 141.2, 135.3, 134.1, 133.7, 130.3, 129.5, 128.9, 128.7, 120.2, 108.8, 101.3, 91.6, 74.0, 66.1, 55.6, 21.0, 4.2; HRMS (ESI, $[M+H]^+$) for $C_{27}H_{27}N_2O_4^+$ calcd 443.1965; found 443.1974.

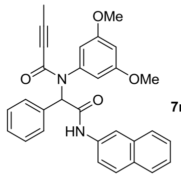

7n

***N*-(3,5-Dimethoxyphenyl)-*N*-(2-(naphthalen-2-ylamino)-2-oxo-1-phenylethyl)but-2-ynamide (7n).** Yield: 57%; yellow solid; mp 96-97°C; ^{1}H NMR (400 MHz, $CDCl_3$): δ 8.25-8.20 (m, 1H), 8.02 (bs, 1H), 7.80-7.71 (m, 3H), 7.47-7.35 (m, 3H), 7.34-7.26 (m, 5H), 6.38 (bs, 2H), 6.35 (t, *J* = 2.1 Hz, 1H), 6.17 (s, 1H), 3.66 (s, 6H), 1.77 (s, 3H); ^{13}C NMR (100 MHz, $CDCl_3$): δ 167.6, 160.4, 155.3, 141.1, 135.4, 133.9, 133.5, 130.7, 130.4, 128.9, 128.7, 128.6, 127.8, 127.6, 126.4, 125.0, 120.1, 116.8, 108.9, 101.3, 91.8, 74.1, 66.2, 55.6, 4.2; HRMS (ESI, $[M+H]^+$) for $C_{30}H_{27}N_2O_4^+$ calcd 479.1965; found 479.1959.

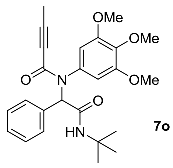

7o

***N*-(2-(*tert*-Butylamino)-2-oxo-1-phenylethyl)-*N*-(3,4,5-trimethoxyphenyl)but-2-ynamide (7o).** Yield: 96%; white solid; mp 149-150°C; ^{1}H NMR (400 MHz, $CDCl_3$): δ 7.26-7.18 (m, 3H), 7.18-7.11 (m, 2H), 6.35 (bs, 2H), 5.95 (s, 1H), 5.57 (bs, 1H), 3.79 (s, 3H), 3.66 (s, 6H), 1.74 (s, 3H), 1.33 (s, 9H);

^{13}C NMR (100 MHz, CDCl$_3$) δ 168.0, 155.0, 152.5, 137.9, 135.1, 134.5, 130.5, 128.6, 128.5, 108.4, 91.1, 74.1, 65.3, 61.0, 56.2, 51.7, 28.7, 4.1; HRMS (ESI, [M+H]$^+$) for $C_{25}H_{31}N_2O_5^+$ calcd 439.2227; found 439.2238.

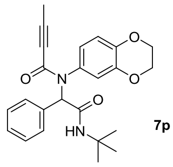

7p

***N*-(2-(*tert*-Butylamino)-2-oxo-1-phenylethyl)-*N*-(2,3-dihydrobenzo[*b*][1,4]dioxin-6-yl)but-2-ynamide (7p).** Yield: 92%; white solid; mp 170-172°C; ^{1}H NMR (400 MHz, CDCl$_3$): δ 7.26-7.12 (m, 5H), 6.78-6.54 (m, 3H), 5.83 (s, 1H), 5.66 (bs, 1H), 4.26-4.13 (m, 4H), 1.74 (s, 3H), 1.33 (s, 9H); ^{13}C NMR (100 MHz, CDCl$_3$): δ 168.1, 155.3, 143.4, 142.9, 134.4, 133.2, 130.3, 128.50, 128.46, 123.9, 119.5, 116.6, 91.3, 74.2, 65.8, 64.4, 64.2, 51.7, 28.7, 4.2; HRMS (ESI, [M+H]$^+$) for $C_{24}H_{27}N_2O_4^+$ calcd 407.1965; found 407.1973.

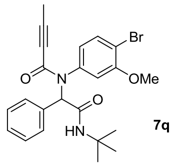

7q

***N*-(4-Bromo-3-methoxyphenyl)-*N*-(2-(*tert*-butylamino)-2-oxo-1-phenylethyl)but-2-ynamide (7q).** Yield: 66%; white solid; mp 172-173°C; ^{1}H NMR (400 MHz, CDCl$_3$): δ 7.32 (d, J = 8.4 Hz, 1H), 7.26-7.18 (m, 3H), 7.16-7.10 (m, 2H), 6.74-6.63 (m, 2H), 5.98 (s, 1H), 5.48 (bs, 1H), 3.70 (s, 3H), 1.72 (s, 3H), 1.33 (s, 9H); ^{13}C NMR (100 MHz, CDCl$_3$): δ 168.0, 155.5, 154.8, 139.9, 134.3, 132.5, 130.4, 128.8, 128.7, 124.6, 114.9, 111.5, 91.6, 74.0, 64.9, 56.4, 51.9, 28.7, 4.1; HRMS (ESI, [M+H]$^+$) for $C_{23}H_{26}BrN_2O_3^+$ calcd 457.1121; found 457.1127.

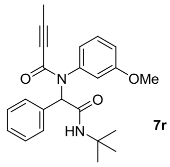

7r

***N*-(2-(*tert*-Butylamino)-2-oxo-1-phenylethyl)-*N*-(3-methoxyphenyl)but-2-ynamide (7r).** Yield: 84%; white solid; ^{1}H NMR (400 MHz, CDCl$_3$): δ 7.26-7.13 (m, 5H), 7.08 (t, J = 8.1 Hz, 1H), 6.82-6.62 (m, 3H), 5.89 (s, 1H), 5.64 (bs, 1H), 3.66 (s, 3H), 1.71 (bs, 3H), 1.34 (s, 9H); ^{13}C NMR (100 MHz, CDCl$_3$): δ 168.0, 159.4, 154.9, 140.8, 134.3, 130.2, 128.9, 128.42, 128.36, 123.0, 115.5, 114.5, 91.1, 74.1, 65.6, 55.3, 51.6, 28.6, 3.9; HRMS (ESI, [M+H]$^+$) for $C_{23}H_{27}N_2O_3^+$ calcd 379.2016; found 379.2010.

General procedure for the gold-catalyzed synthesis of 2-quinolones 8

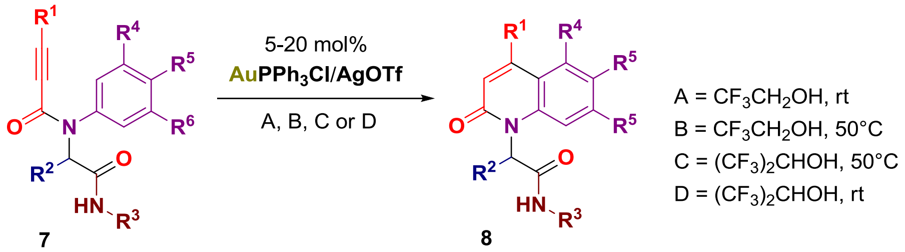

The Ugi adduct **7** (0.5 mmol) was dissolved in TFE (5 mL) followed by the addition of $AuPPh_3Cl$ (12.4 mg, 0.025 mmol) and AgOTf (6.4 mg, 0.025 mmol). The reaction mixture was sealed and stirred at room temperature for 12 h. The resulting mixture was diluted with EtOAc and concentrated with silica. Column chromatography with petroleum ether/EtOAc (the ratio was adjusted according to TLC) as eluent delivered 2-quinolone **8**. Products **8m** and **8n** were washed by pentane after the column chromatography. For the substrates **7c–e,j,o**, the reactions were conducted at 50 °C. For the substrate **7p**, the reaction was conducted on 0.3 mmol scale for 20 h at 50 °C using HFIP (3 mL) as the solvent. For the substrate **7q**, the reaction was conducted on 0.4 mmol scale for 30 h at 50 °C using $AuPPh_3Cl$ (39.7 mg, 0.08 mmol), AgOTf (20.6 mg, 0.08 mmol) and HFIP (4 mL). For the substrate **7r**, the reaction was conducted on 0.4 mmol scale for 12 h at room temperature using HFIP (4 mL) as the solvent.

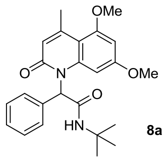

8a

***N*-(*tert*-Butyl)-2-(5,7-dimethoxy-4-methyl-2-oxoquinolin-1(2*H*)-yl)-2-phenylacetamide (8a).** Yield: 96%; white solid; mp 180-181°C; ^{1}H NMR (400 MHz, $CDCl_3$) δ 7.29-7.17 (m, 5H), 7.08 (bs, 1H), 6.59 (d, *J* = 2.0 Hz, 1H), 6.38 (s, 1H), 6.21 (d, *J* = 2.0 Hz, 1H), 6.12 (bs, 1H), 3.83 (s, 3H), 3.54 (s, 3H), 2.64 (s, 3H), 1.29 (s, 9H); ^{13}C NMR (100 MHz, $CDCl_3$): δ 167.9, 163.0, 161.4, 159.8, 150.0, 141.9, 134.4, 128.3, 127.6, 127.3, 117.5, 107.5, 94.7, 94.5, 59.7 (bs), 55.60, 55.57, 51.7, 28.7, 25.2; IR (thin film, cm^{-1}): 3282, 2962, 2924, 2851, 1686, 1636, 1606, 1577, 1545, 1457, 1362, 1264, 1205, 1173, 1152, 1101, 1033, 817, 721; HRMS (ESI, $[M+Na]^+$) for $C_{24}H_{28}N_2O_4Na^+$ calcd 431.1941; found 431.1951.

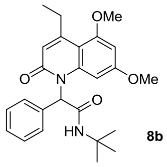

8b

***N*-(*tert*-Butyl)-2-(4-ethyl-5,7-dimethoxy-2-oxoquinolin-1(2*H*)-yl)-2-phenylacetamide (8b).** Yield: 97%; yellow solid; mp 168-169°C; ^{1}H NMR (400 MHz, $CDCl_3$): δ 7.30-7.17 (m, 5H), 7.08 (bs, 1H), 6.62 (d, *J* = 2.1 Hz, 1H), 6.43 (s, 1H), 6.23 (d, *J* = 2.1 Hz, 1H), 6.13 (bs, 1H), 3.86 (s, 3H), 3.54 (s, 3H), 3.15-2.97 (m, 2H), 1.29 (s, 9H), 1.28 (t, *J* = 7.4 Hz, 3H); ^{13}C NMR (100 MHz, $CDCl_3$): δ 167.9, 163.3, 161.2, 159.4, 155.4, 142.2, 134.5, 128.3, 127.6, 127.3, 115.9, 106.9, 94.8, 94.7, 59.7, 55.62, 55.56, 51.7,

30.3, 28.7, 14.5; IR (thin film, cm^{-1}): 3251, 3053, 2966, 2924, 2852, 1681, 1644, 1605, 1590, 1580, 1547, 1457, 1424, 1391, 1353, 1276, 1256, 1203, 1176, 1149, 1109, 826, 748, 692; HRMS (ESI, $[M+Na]^+$) for $C_{25}H_{30}N_2O_4Na^+$ calcd 445.2098; found 445.2104.

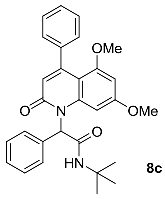

8c

***N*-(*tert*-Butyl)-2-(5,7-dimethoxy-2-oxo-4-phenylquinolin-1(2*H*)-yl)-2-phenylacetamide (8c).** Yield: 83%; white solid; mp 153-154°C; ^{1}H NMR (400 MHz, $CDCl_3$): δ 7.41-7.21 (m, 10H), 7.05 (bs, 1H), 6.68 (d, *J* = 1.9 Hz, 1H), 6.41 (s, 1H), 6.19 (bs, 1H), 6.14 (d, *J* = 2.0 Hz, 1H), 3.58 (s, 3H), 3.36 (s, 3H), 1.33 (s, 9H); ^{13}C NMR (100 MHz, $CDCl_3$): δ 167.7, 162.8, 162.0, 158.7, 151.6, 142.2, 142.0, 134.6, 128.5, 127.7, 127.5, 127.3, 127.2, 118.5, 105.9, 95.0, 94.5, 60.4 (bs), 55.6, 55.3, 51.8, 28.7; IR (thin film, cm^{-1}): 3258, 3058, 2974, 1683, 1636, 1598, 1556, 1460, 1390, 1361, 1258, 1207, 1167, 826, 696; HRMS (ESI, $[M+Na]^+$) for $C_{29}H_{30}N_2O_4Na^+$ calcd 493.2098; found 493.2107.

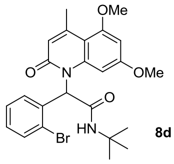

8d

2-(2-Bromophenyl)-*N*-(*tert*-butyl)-2-(5,7-dimethoxy-4-methyl-2-oxoquinolin-1(2*H*)-yl)acetamide (8d). Yield: 93%; white solid; mp 233-234°C; ^{1}H NMR (400 MHz, $CDCl_3$): δ 7.67-7.59 (m, 1H), 7.47-7.39 (m, 1H), 7.28-7.14 (m, 2H), 6.51-6.37 (m, 2H), 6.33 (s, 1H), 6.28-6.22 (m, 1H), 5.54 (s, 1H), 3.84 (s, 3H), 3.72 (s, 3H), 2.60 (s, 3H), 1.30 (s, 9H); ^{13}C NMR (100 MHz, $CDCl_3$): δ 166.0, 162.7, 161.9, 160.0, 149.6, 143.4, 134.3, 133.3, 130.0, 129.9, 128.1, 124.8, 118.5, 107.5, 94.6, 92.0, 64.7, 55.70, 55.66, 51.7, 28.6, 25.2; IR (thin film, cm^{-1}): 3326, 2965, 1685, 1622, 1590, 1542, 1472, 1457, 1394, 1341, 1201, 1154, 1043, 813, 734, 699; HRMS (ESI, $[M+Na]^+$) for $C_{24}H_{27}BrN_2O_4Na^+$ calcd 509.1046; found 509.1048.

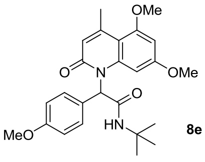

8e

***N*-*tert*-Butyl-2-(5,7-dimethoxy-4-methyl-2-oxoquinolin-1(2*H*)-yl)-2-(4-methoxyphenyl)acetamide (8e).** Yield: 96%; wlite solid; mp 166-167°C; ^{1}H NMR (400 MHz, $CDCl_3$): δ 7.23 (d, *J* = 8.8 Hz, 2H), 6.98 (bs, 1H), 6.80 (d, *J* = 8.9 Hz, 2H), 6.59 (d, J = 2.2 Hz, 1H), 6.37 (q, J = 0.7 Hz, 1H), 6.21 (d, *J* = 2.2 Hz, 1H), 6.03 (bs, 1H), 3.83 (s, 3H), 3.76 (s, 3H), 3.59 (s, 3H), 2.63 (d, J = 0.7 Hz, 3H), 1.28 (s, 9H); ^{13}C NMR (100 MHz, $CDCl_3$): δ 168.1, 162.9, 161.4, 159.8, 158.7, 149.8, 142.0, 129.0, 126.5, 117.6, 113.8, 107.5, 94.6, 94.3, 59.4, 55.6, 55.3, 51.6, 28.7, 25.2; IR (thin film, cm^{-1}): 3219, 3047, 2966, 1681, 1636, 1607, 1589, 1558, 1514, 1455, 1389, 1273, 1252, 1203, 1181, 1150, 1102, 1032, 832; HRMS (ESI, $[M+Na]^+$) for $C_{25}H_{30}N_2O_5Na^+$ calcd 461.2047; found 461.2052.

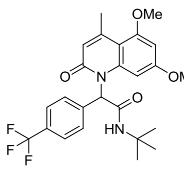

8f

***N*-(*tert*-Butyl)-2-(5,7-dimethoxy-4-methyl-2-oxoquinolin-1(2*H*)-yl)-2-(4-(trifluoromethyl)phenyl)acetamide (8f).** Yield: 93%; yellow solid; mp 147-148°C; ^{1}H NMR (400 MHz, $CDCl_3$): δ 7.51 (d, *J* = 8.3 Hz, 2H), 7.37 (d, *J* = 8.2 Hz, 2H), 7.24 (bs, 1H), 6.49 (d, *J* = 1.9 Hz, 1H), 6.39 (s, 1H), 6.24 (d, *J* = 2.0 Hz, 1H), 6.14 (bs, 1H), 3.85 (s, 3H), 3.53 (s, 3H), 2.66 (s, 3H), 1.28 (s, 9H); ^{13}C NMR (100 MHz, $CDCl_3$): δ 167.4, 162.8, 161.7, 159.9, 150.5, 141.5, 138.4, 129.4 (q, *J* = 32.5 Hz), 128.0, 125.1 (q, *J* = 3.7 Hz), 124.2 (q, *J* = 272.0 Hz), 117.2, 107.4, 95.0, 94.2, 58.6 (bs), 55.7, 55.6, 51.9, 28.6, 25.3; IR (thin film, cm^{-1}): 3309, 2963, 2933, 1678, 1641, 1609, 1586, 1543, 1456, 1392, 1321, 1210, 1172, 1144, 1128, 1117, 1101, 1069, 835; HRMS (ESI, $[M+Na]^+$) for $C_{25}H_{27}F_3N_2O_4Na^+$ calcd 499.1815; found 499.1827.

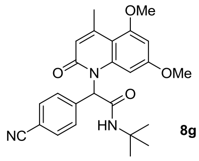

8g

***N*-(*tert*-Butyl)-2-(4-cyanophenyl)-2-(5,7-dimethoxy-4-methyl-2-oxoquinolin-1(2*H*)-yl)acetamide (8g).** Yield: 92%; white solid; mp 162-163°C; ^{1}H NMR (400 MHz, $CDCl_3$): δ 7.55 (d, *J* = 8.4 Hz, 2H), 7.36 (d, *J* = 8.2 Hz, 2H), 7.27 (bs, 1H), 6.42 (d, *J* = 1.9 Hz, 1H), 6.39 (s, 1H), 6.25 (d, *J* = 2.1 Hz, 1H), 6.13 (bs, 1H), 3.86 (s, 3H), 3.52 (s, 3H), 2.67 (s, 3H), 1.27 (s, 9H); ^{13}C NMR (100 MHz, $CDCl_3$): δ 167.1, 162.7, 161.8, 160.0, 150.7, 141.3, 139.7, 131.8, 128.5, 118.8, 117.0, 111.0, 107.4, 95.1, 94.1, 58.3 (bs), 55.7, 55.6, 51.9, 28.6, 25.3; IR (thin film, cm^{-1}): 3233, 2924, 2852, 1641, 1608, 1589, 1540, 1506, 1457, 1424, 1389, 1272, 1203, 1172, 1152, 1099, 824; HRMS (ESI, $[M+Na]^+$) for $C_{25}H_{27}N_3O_4Na^+$ calcd 456.1894; found 456.1896.

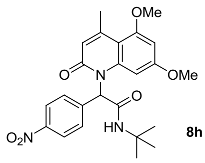

8h

***N*-(*tert*-Butyl)-2-(5,7-dimethoxy-4-methyl-2-oxoquinolin-1(2*H*)-yl)-2-(4-nitrophenyl)acetamide (8h).** Yield: 90%; white solid; mp 153-154°C; ^{1}H NMR (400 MHz, $CDCl_3$): δ 8.11 (d, *J* = 8.9 Hz, 2H), 7.43 (d, *J* = 8.9 Hz, 2H), 7.31 (bs, 1H), 6.43 (d, *J* = 2.0 Hz, 1H), 6.40 (s, 1H), 6.25 (d, *J* = 2.1 Hz, 1H), 6.15 (bs, 1H), 3.87 (s, 3H), 3.53 (s, 3H), 2.68 (d, *J* = 0.8 Hz, 3H), 1.28 (s, 9H); ^{13}C NMR (100 MHz, $CDCl_3$): δ 167.0, 162.7, 161.9, 160.1, 150.8, 146.9, 141.8, 141.3, 128.7, 123.2, 117.0, 107.4, 95.1, 94.1, 58.2 (bs), 55.7, 55.6, 52.0, 28.6, 25.3; IR (thin film, cm^{-1}): 3233, 2961, 2932, 1644, 1609, 1590, 1519, 1456, 1389, 1349, 1272, 1205, 1153, 1100, 822, 741; HRMS (ESI, $[M+H]^+$) for $C_{24}H_{28}N_3O_6^+$ calcd 454.1973; found 454.1952.

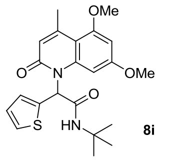

8i

***N*-(*tert*-Butyl)-2-(5,7-dimethoxy-4-methyl-2-oxoquinolin-1(2*H*)-yl)-2-(thiophen-2-yl)acetamide (8i).** Yield: 94%; yellow solid; mp 186-187°C; ^{1}H NMR (400 MHz, $CDCl_3$): δ 7.49 (bs, 1H), 7.25-7.21 (m, 1H), 6.86 (dd, *J* = 5.0, 3.7 Hz, 1H), 6.84-6.79 (m, 1H), 6.50 (d, *J* = 2.1 Hz, 1H), 6.38 (q, *J* = 0.9 Hz, 1H), 6.21 (d, *J* = 2.2 Hz, 1H), 5.77 (bs, 1H), 3.83 (s, 3H), 3.66 (s, 3H), 2.63 (d, *J* = 0.8 Hz, 3H), 1.27 (s, 9H); ^{13}C NMR (100 MHz, $CDCl_3$): δ 167.6, 162.4, 161.6, 159.8, 150.3, 141.0, 136.3, 126.7, 126.4, 126.0, 117.0, 107.5, 94.9, 93.3, 55.7, 55.6, 55.4 (bs), 51.8, 28.6, 25.3; IR (thin film, cm^{-1}): 3255, 3056, 2957, 2924, 2852, 1674, 1645, 1590, 1546, 1455, 1387, 1260, 1207, 1191, 1148, 1100, 1034, 816, 696; HRMS (ESI, $[M+Na]^+$) for $C_{22}H_{26}N_2O_4SNa^+$ calcd 437.1505; found 437.1508.

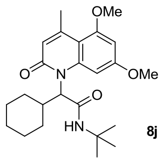

8j

***N*-(*tert*-Butyl)-2-cyclohexyl-2-(5,7-dimethoxy-4-methyl-2-oxoquinolin-1(2*H*)-yl)acetamide (8j).** Yield: 94%; yellow solid; mp 158-159°C; in $CDCl_3$ observed as ~ 1:8 mixture of rotamers; ^{1}H NMR (400 MHz, $CDCl_3$, major rotamer): δ 7.04 (d, *J* = 1.9 Hz, 1H), 6.33-6.23 (m, 2H), 5.84 (bs, 1H), 5.80 (d, *J* = 10.6 Hz, 1H), 3.86 (s, 3H), 3.83 (s, 3H), 2.61-2.48 (m, 4H), 2.23-2.11 (m, 1H), 1.77-1.66 (m, 1H), 1.63-1.43 (m, 2H), 1.40-1.24 (m, 1H), 1.21 (s, 9H), 1.16-0.93 (m, 3H), 0.88-0.63 (m, 2H); ^{13}C NMR (100 MHz, $CDCl_3$, major rotamer): δ 169.5, 163.5, 161.9, 159.8, 149.3, 142.3, 117.5, 107.0, 94.9, 92.3, 60.5, 55.9, 55.6, 51.4, 35.1, 32.6, 28.7, 27.4, 26.3, 26.0, 25.9, 25.1; IR (thin film, cm^{-1}): 3274, 2925, 2852, 1668, 1640, 1609, 1588, 1549, 1457, 1397, 1270, 1205, 1156, 1095, 826; HRMS (ESI, $[M+Na]^+$) for $C_{24}H_{34}N_2O_4Na^+$ calcd 437.2411; found 437.2405.

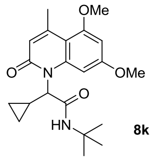

8k

***N*-(*tert*-Butyl)-2-cyclopropyl-2-(5,7-dimethoxy-4-methyl-2-oxoquinolin-1(2*H*)-yl)acetamide (8k).** Yield: 99%; yellow solid; mp 160-161°C; ^{1}H NMR (400 MHz, $CDCl_3$) δ 6.78 (bs, 1H), 6.31-6.26 (m, 2H), 5.74 (bs, 1H), 5.48 (bd, *J* = 7.3 Hz, 1H), 3.86 (s, 6H), 2.60 (d, *J* = 0.6 Hz, 3H), 1.95-1.76 (m, 1H), 1.27 (s, 9H), 0.91-080 (s, 1H), 0.63-0.53 (m, 1H), 0.41-0.19 (m, 2H); ^{13}C NMR (100 MHz, $CDCl_3$): δ 169.3, 162.9, 161.7, 160.0, 149.4, 141.6 (bs), 117.5, 107.5, 94.5, 92.9, 61.1 (bs), 55.7, 55.6, 51.5, 28.7, 25.1, 10.5 (bs), 7.4 (bs), 2.7 (bs); IR (thin film, cm^{-1}): 3337, 3005, 2970, 2933, 1686, 1640, 1607, 1580, 1529, 1457, 1389, 1207, 1171, 1148, 1095, 841, 816; HRMS (ESI, $[M+Na]^+$) for$C_{21}H_{28}N_2O_4$ Na^+ calcd 395.1941; found 395.1948.

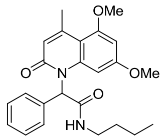

8l

***N*-Butyl-2-(5,7-dimethoxy-4-methyl-2-oxoquinolin-1(2*H*)-yl)-2-phenylacetamide (8l).** Yield: 97%; yellow solid; mp 159-160°C; ^{1}H NMR (400 MHz, $CDCl_3$): δ 7.35-7.19 (m, 5H), 7.12 (bs, 1H), 6.61 (d, *J* = 2.1 Hz, 1H), 6.45-6.34 (m, 2H), 6.20 (d, *J* = 2.1 Hz, 1H), 3.83 (s, 3H), 3.53 (s, 3H), 3.33-3.18 (m, 2H), 2.63 (d, *J* = 0.7 Hz, 3H), 1.49-1.36 (m, 2H), 1.30-1.14 (m, 2H), 0.84 (t, *J* = 7.3 Hz, 1H); ^{13}C NMR (100 MHz, $CDCl_3$): δ 168.5, 163.1, 161.4, 159.8, 150.0, 141.8, 134.6, 128.5, 127.5, 127.4, 117.5, 107.5, 94.68, 94.66, 59.2 (bs), 55.6, 55.5, 39.6, 31.5, 25.2, 20.1, 13.8; IR (thin film, cm^{-1}): 3299, 3242, 2954, 2926, 2851, 1676, 1650, 1610, 1590, 1541, 1460, 1391, 1354, 1273, 1209, 1151, 1101, 832; HRMS (ESI, $[M+Na]^+$) for $C_{24}H_{28}N_2O_4Na^+$ calcd 431.1941; found 431.1947.

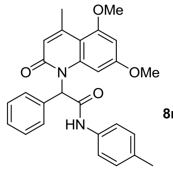

8m

2-(5,7-Dimethoxy-4-methyl-2-oxoquinolin-1(2*H*)-yl)-2-phenyl-*N*-(*p*-tolyl)acetamide (8m). Yield: 89%; white solid; ^{1}H NMR (400 MHz, $CDCl_3$): δ 8.60 (bs, 1H), 7.39 (d, *J* = 8.3 Hz, 2H), 7.36-7.20 (m, 6H), 7.09 (d, *J* = 8.2 Hz, 2H), 6.70 (d, *J* = 2.0 Hz, 1H), 6.40 (s, 1H), 6.21 (d, *J* = 2.0 Hz, 1H), 3.83 (s, 3H), 3.56 (s, 3H), 2.64 (s, 3H), 2.29 (s, 3H); ^{13}C NMR (100 MHz, $CDCl_3$): δ 166.7, 163.4, 161.6, 159.8, 150.4, 142.0, 135.3, 134.2, 129.5, 128.6, 127.6, 127.3, 120.3, 117.4, 107.7, 94.9, 94.6, 60.3 (bs), 55.7, 55.6, 25.2, 21.0; IR (thin film, cm^{-1}): 3048, 3002, 1687, 1640, 1605, 1590, 1513, 1457, 1393, 1273, 1204, 1150, 1102, 825; HRMS (ESI, $[M+Na]^+$) for $C_{27}H_{26}N_2O_4Na^+$ calcd 465.1785; found 465.1769.

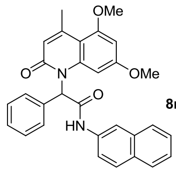

8n

2-(5,7-Dimethoxy-4-methyl-2-oxoquinolin-1(2*H*)-yl)-*N*-(naphthalen-2-yl)-2-phenylacetamide (8n). Yield: 83%; yellow solid; mp 238-239°C; ^{1}H NMR (400 MHz, $CDCl_3$): δ 8.96 (bs, 1H), 8.25 (d, *J* = 1.4 Hz, 1H), 7.79-7.72 (m, 3H), 7.48-7.24 (m, 9H), 6.73 (d, *J* = 2.0 Hz, 1H), 6.42 (s, 1H), 6.22 (d, *J* = 1.9 Hz, 1H), 3.83 (s, 3H), 3.59 (s, 3H), 2.64 (s, 3H); ^{13}C NMR (100 MHz, $CDCl_3$): δ 167.1, 163.5, 161.6, 159.9, 150.5, 142.0, 135.4, 134.2, 133.9, 130.8, 128.72, 128.66, 127.8, 127.7, 127.6, 127.3, 126.5, 125.1, 120.2, 117.4, 117.0, 107.7, 94.9, 94.5, 60.8 (bs), 55.6, 25.2; IR (thin film, cm^{-1}): 3085, 2936, 2842, 1699, 1634, 1606, 1572, 1498, 1355, 1264, 1146, 856, 815, 741; HRMS (ESI, $[M+Na]^+$) for $C_{30}H_{26}N_2O_4Na^+$ calcd 501.1785; found 501.1795.

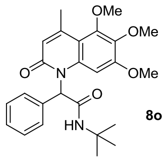

8o

***N*-(*tert*-Butyl)-2-phenyl-2-(5,6,7-trimethoxy-4-methyl-2-oxoquinolin-1(2*H*)-yl)acetamide (8o).** Yield: 89%; white solid; mp 147-148°C; ^{1}H NMR (400 MHz, $CDCl_3$): δ 7.33-7.19 (m, 5H), 7.11 (bs, 1H), 6.85 (s, 1H), 6.48-6.43 (m, 1H), 6.21 (bs, 1H), 3.94 (s, 3H), 3.81 (s, 3H), 3.56 (s, 3H), 2.66 (s, 3H), 1.30 (s, 9H); ^{13}C NMR (100 MHz, $CDCl_3$): δ 167.8, 162.8, 154.8, 152.0, 148.9, 138.3, 136.2, 134.5, 128.4, 127.5, 127.4, 119.0, 110.9, 98.3, 61.4, 60.9, 59.4 (bs), 56.0, 51.8, 28.7, 23.9; IR (thin film, cm^{-1}): 3301, 3065, 2963, 2933, 1636, 1545, 1456, 1398, 1278, 1123, 1099, 1015, 826, 699, 627; HRMS (ESI, $[M+Na]^+$) for $C_{25}H_{30}N_2O_5Na^+$ calcd 461.2047; found 461.2051.

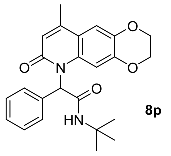

8p

***N*-(*tert*-Butyl)-2-(9-methyl-7-oxo-2,3-dihydro-[1,4]dioxino[2,3-*g*]quinolin-6(7*H*)-yl)-2-phenylacetamide (8p).** Yield: 58%; white solid; mp 202-203°C; ^{1}H NMR (400 MHz, $CDCl_3$): δ 7.35-7.23 (m, 5H), 7.15 (s, 1H), 6.92 (s, 1H), 6.66 (bs, 1H), 6.55-6.49 (m, 1H), 5.96 (bs, 1H), 4.23 (s, 4H), 2.41 (s, 3H), 1.30 (s, 9H); ^{13}C NMR (100 MHz, $CDCl_3$): δ 167.4, 162.7, 147.3, 146.0, 139.6, 134.9, 134.5, 128.8, 127.94, 127.86, 119.0, 116.8, 112.4, 105.4, 64.8, 64.3, 61.6 (bs), 51.8, 28.7, 19.4; IR (thin film, cm^{-1}): 3288, 3056, 2929, 2873, 1683, 1644, 1580, 1543, 1439, 1403, 1321, 1289, 1260, 1069, 894, 872, 726; HRMS (ESI, $[M+Na]^+$) for $C_{24}H_{26}N_2O_4Na^+$ calcd 429.1785; found 429.1792.

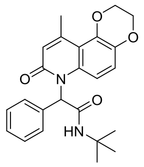

8p'

***N*-(*tert*-Butyl)-2-(10-methyl-8-oxo-2,3-dihydro-[1,4]dioxino[2,3-*f*]quinolin-7(8*H*)-yl)-2-phenylacetamide (8p').** Yield: 15%; ^{1}H NMR (400 MHz, $CDCl_3$): δ 7.33-7.20 (m, 5H), 6.90 (d, *J* = 9.4 Hz, 1H), 6.87 (d, *J* = 9.4 Hz, 1H), 6.85 (bs, 1H), 6.51 (q, *J* = 1.0 Hz, 1H), 6.01 (bs, 1H), 4.37-4.30 (m, 2H), 4.29-4.22 (m, 2H), 2.67 (d, *J* = 1.0 Hz, 3H), 1.28 (s, 9H); ^{13}C NMR (100 MHz, $CDCl_3$): δ 167.7, 162.2, 148.6, 141.6, 138.5, 134.7, 134.6, 128.6, 127.8, 127.6, 121.3, 119.9, 113.4, 110.5, 64.4, 63.7, 61.0 (bs), 51.7, 28.7, 25.0; HRMS (ESI, $[M+Na]^+$) for $C_{24}H_{26}N_2O_4Na^+$ calcd 429.1785; found 429.1766.

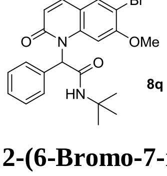

8q

2-(6-Bromo-7-methoxy-4-methyl-2-oxoquinolin-1(2*H*)-yl)-*N*-(*tert*-butyl)-2-phenylacetamide (8q). Yield: 75%; white solid; mp 166-167°C; ^{1}H NMR (400 MHz, $CDCl_3$): δ 7.81 (s, 1H), 7.31-7.20 (m, 5H), 7.14 (bs, 1H), 7.08 (m, 1H), 6.57 (q, *J* = 0.9 Hz, 1H), 6.19 (bs, 1H), 3.57 (s, 3H), 2.47 (d, *J* = 0.9 Hz, 3H), 1.31 (s, 9H); ^{13}C NMR (100 MHz, $CDCl_3$): δ 167.5, 163.1, 155.7, 147.4, 139.0, 134.3, 129.2,

128.6, 127.6, 127.5, 118.2, 117.0, 106.4, 102.5, 58.9 (bs), 56.6, 51.9, 28.7, 19.3; IR (thin film, cm^{-1}): 3327, 2964, 2922, 1687, 1609, 1582, 1541 1385, 1362, 1212, 1027, 697; HRMS (ESI, $[M+ Na]^+$) for $C_{23}H_{25}BrN_2O_3Na^+$ calcd 479.0941; found 479.0951.

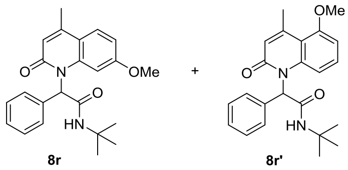

8r *major* **8r'** *minor*

***N*-*tert*-Butyl-2-(7-methoxy-4-methyl-2-oxoquinolin-1(2*H*)-yl)-2-phenylacetamide (8r).** Obtained as a mixture with *N*-*tert*-butyl-2-(5-methoxy-4-methyl-2-oxoquinolin-1(2*H*)-yl)-2-phenylacetamide (**8r'**). Combined yield: 80%; **8r**:**8r'** = 6.5:1; ^{1}H NMR (400 MHz, $CDCl_3$): δ 7.62-7.55 (m, 1H), 7.36-7.18 (m, 5H), 7.08-6.90 (m, 2H), 6.81-6.73 (m, 1H), 6.53 (s, 1H), 6.07 (bs, 1H), 3.59 (s, 3H), 2.48 (s, 3H), 1.30 (s, 9H); ^{13}C NMR (100 MHz, $CDCl_3$): δ 167.7, 163.3, 161.1, 148.2, 140.3, 134.6, 128.5, 127.7, 127.5, 126.4, 117.5, 115.9, 111.6, 101.6, 59.8, 55.6, 51.8, 28.7, 19.4; HRMS (ESI, $[M+H]^+$) for $C_{23}H_{27}N_2O_3^+$ calcd 379.2016; found 379.2011.

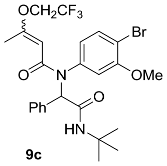

9c

***N*-(4-bromo-3-methoxyphenyl)-*N*-(2-(*tert*-butylamino)-2-oxo-1-phenylethyl)-3-(2,2,2-trifluoroethoxy)but-2-enamide (9c).** Obtained as a byproduct during the synthesis of 2-quinolone **8q**. Yield: 18%; ^{1}H NMR (400 MHz, $CDCl_3$): δ 7.32 (d, *J* = 8.4 Hz, 1H), 7.25-7.17 (m, 3H), 7.16-7.10 (m, 2H), 6.62 (bs, 2H), 5.91 (s, 1H), 5.50 (bs, 1H), 4.82 (s, 1H), 4.53 (dq, *J* = 12.8, 8.6 Hz, 1H), 4.41 (dq, *J* = 12.8, 8.5 Hz, 1H), 3.67 (bs, 3H), 1.79 (s, 3H), 1.32 (s, 9H); ^{13}C NMR (100 MHz, $CDCl_3$): δ 168.9, 165.6, 163.5, 155.8, 140.7, 134.8, 133.0, 130.42, 130.36, 128.6, 124.3, 123.4 (q, *J* = 278.4 Hz), 114.6, 111.2, 100.3, 68.8 (q, *J* = 35.2 Hz), 65.4, 56.4, 51.7, 28.6, 22.0; HRMS (ESI, $[M+H]^+$) for $C_{25}H_{29}BrF_3N_2O^{4+}$ calcd 557.1257; found 557.1193.

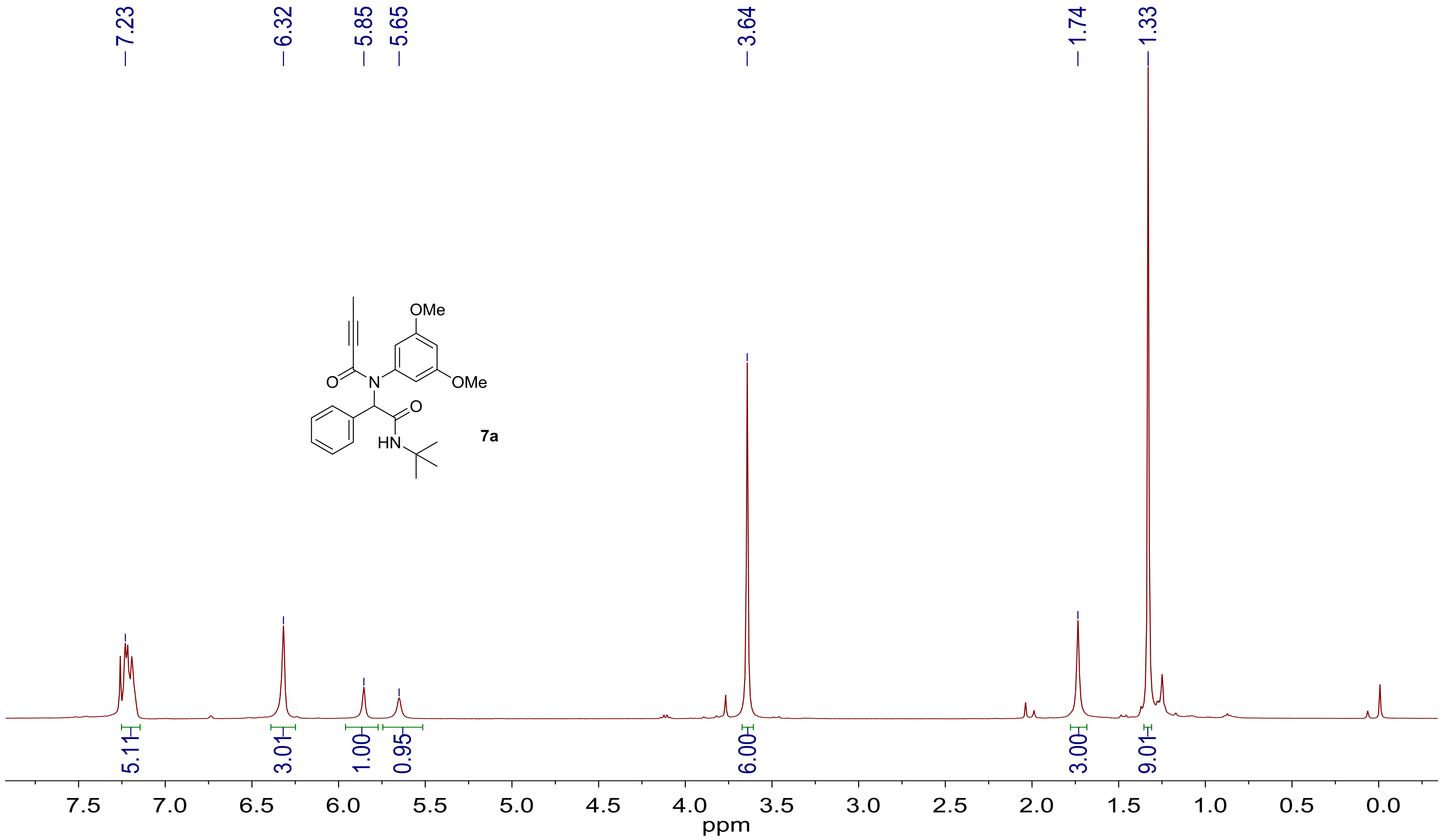
7.23
6.32
5.85
5.65
3.64
1.74
1.33
OMe
OMe
O
N
O
HN
7a
5.11
3.01
1.00
0.95
6.00
3.00
9.01
7.5 7.0 6.5 6.0 5.5 5.0 4.5 4.0 3.5 3.0 2.5 2.0 1.5 1.0 0.5 0.0
ppm

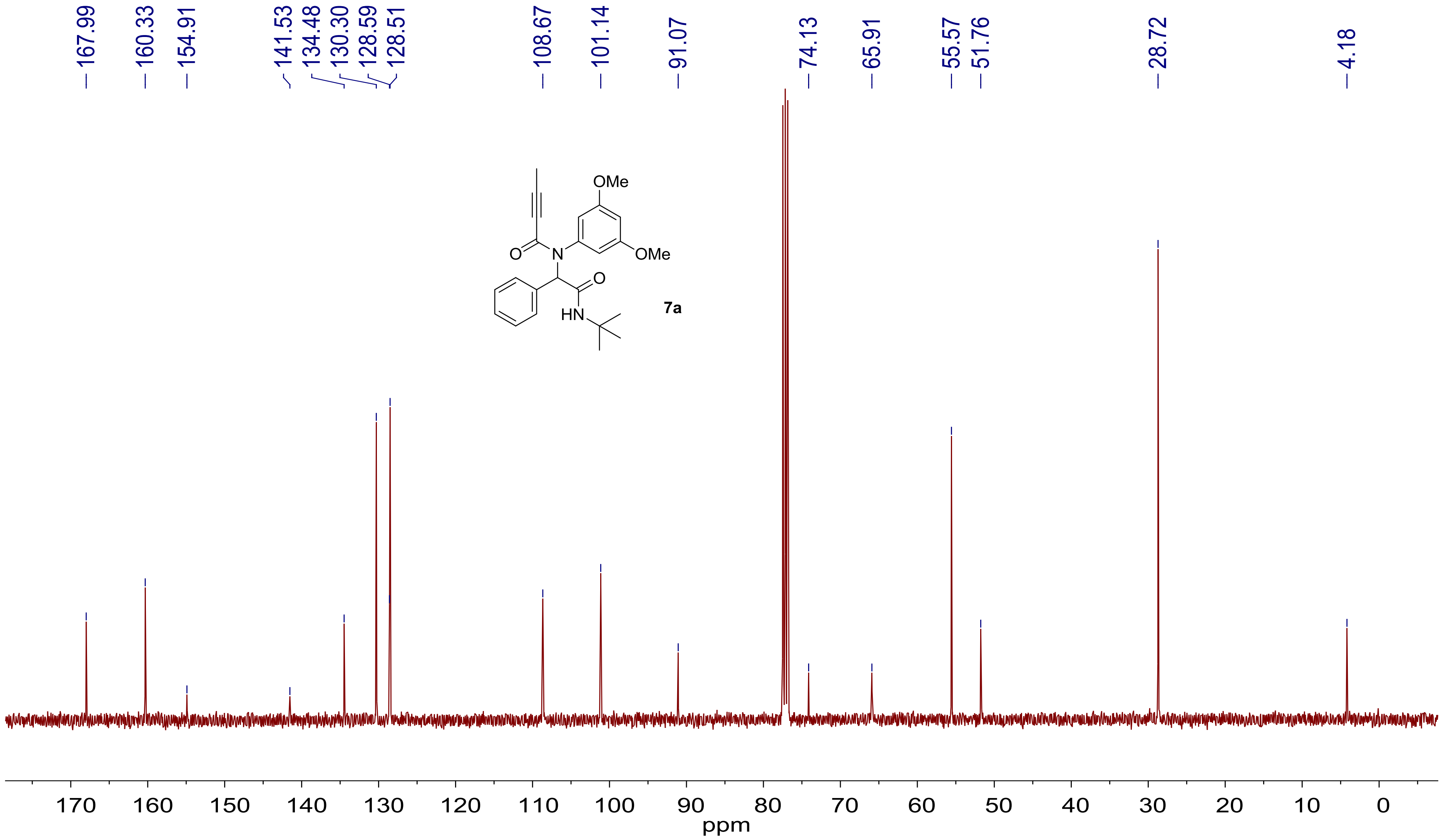
167.99
160.33
154.91
141.53
134.48
130.30
128.59
128.51
108.67
101.14
91.07
74.13
65.91
55.57
51.76
28.72
4.18
OMe
OMe
O
N
O
HN
7a
170
160
150
140
130
120
110
100
90
80
70
60
50
40
30
20
10
0
ppm

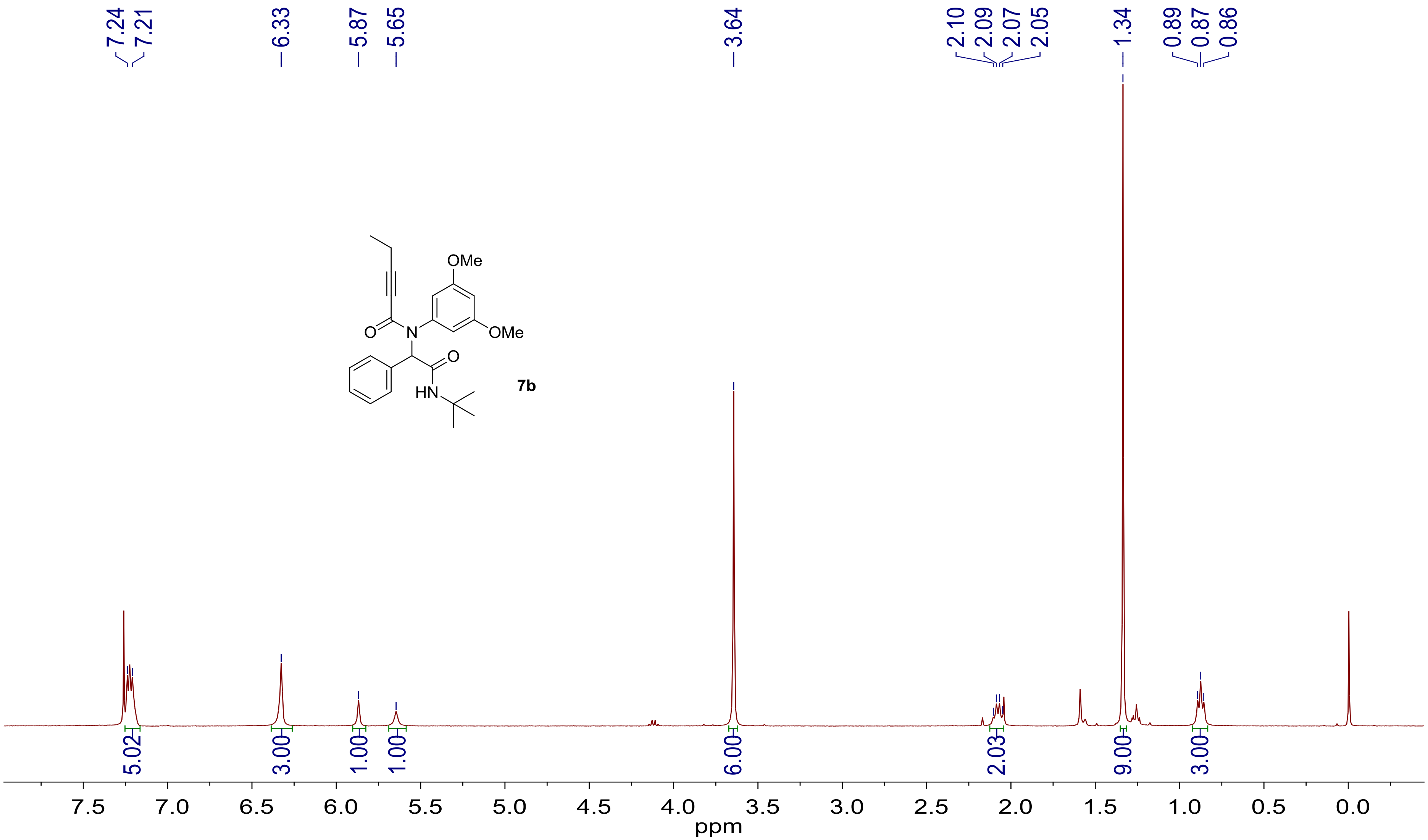
7.24
7.21
6.33
5.87
5.65
3.64
2.10
2.09
2.07
2.05
1.34
0.89
0.87
0.86
OMe
OMe
O
N
O
HN
7b
5.02
3.00
1.00
1.00
6.00
2.03
9.00
3.00
7.5 7.0 6.5 6.0 5.5 5.0 4.5 4.0 3.5 3.0 2.5 2.0 1.5 1.0 0.5 0.0
ppm

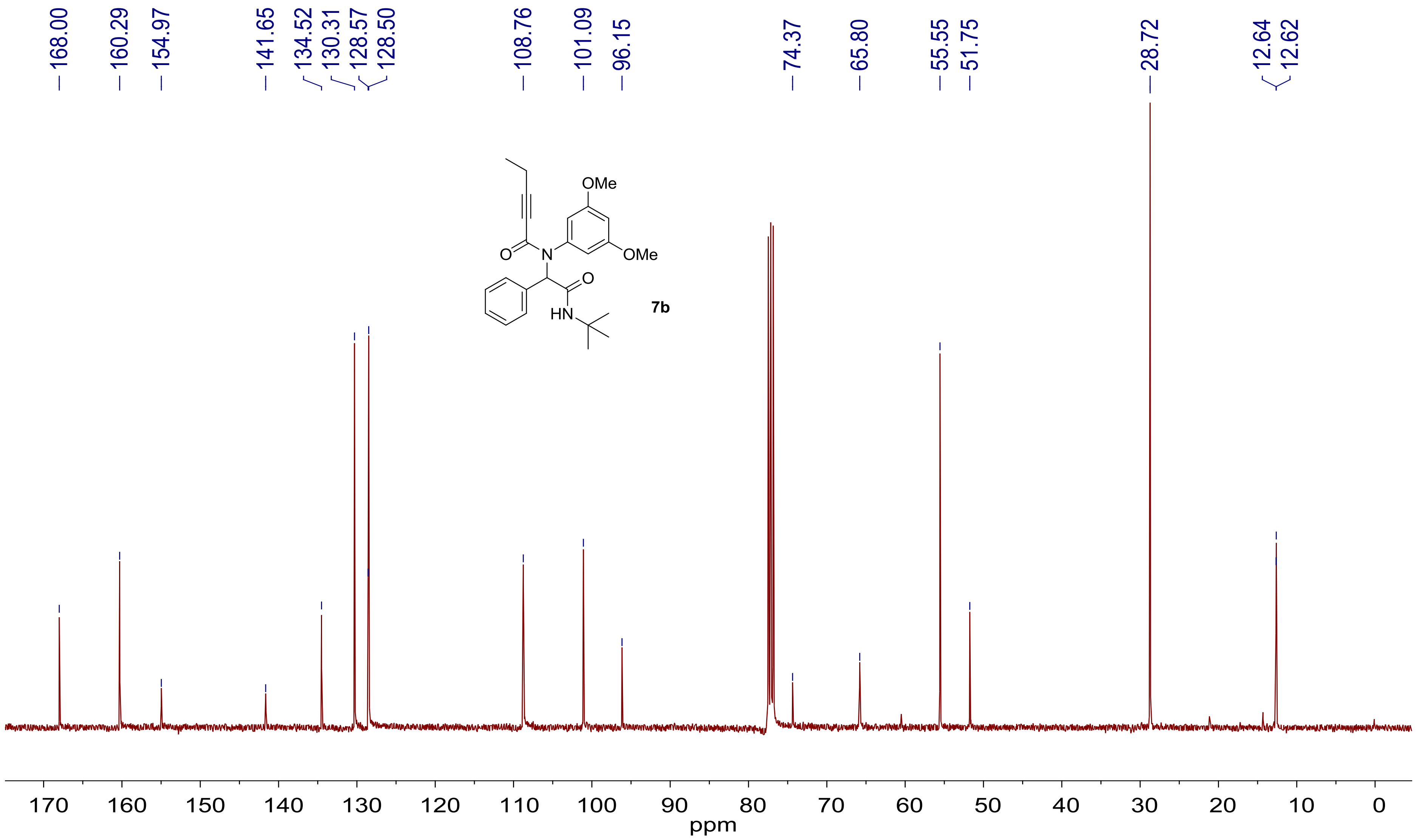

168.00
160.29
154.97
141.65
134.52
130.31
128.57
128.50
108.76
101.09
96.15
74.37
65.80
55.55
51.75
28.72
12.64
12.62
OMe
OMe
O
N
O
HN
7b
170 160 150 140 130 120 110 100 90 80 70 60 50 40 30 20 10 0
ppm

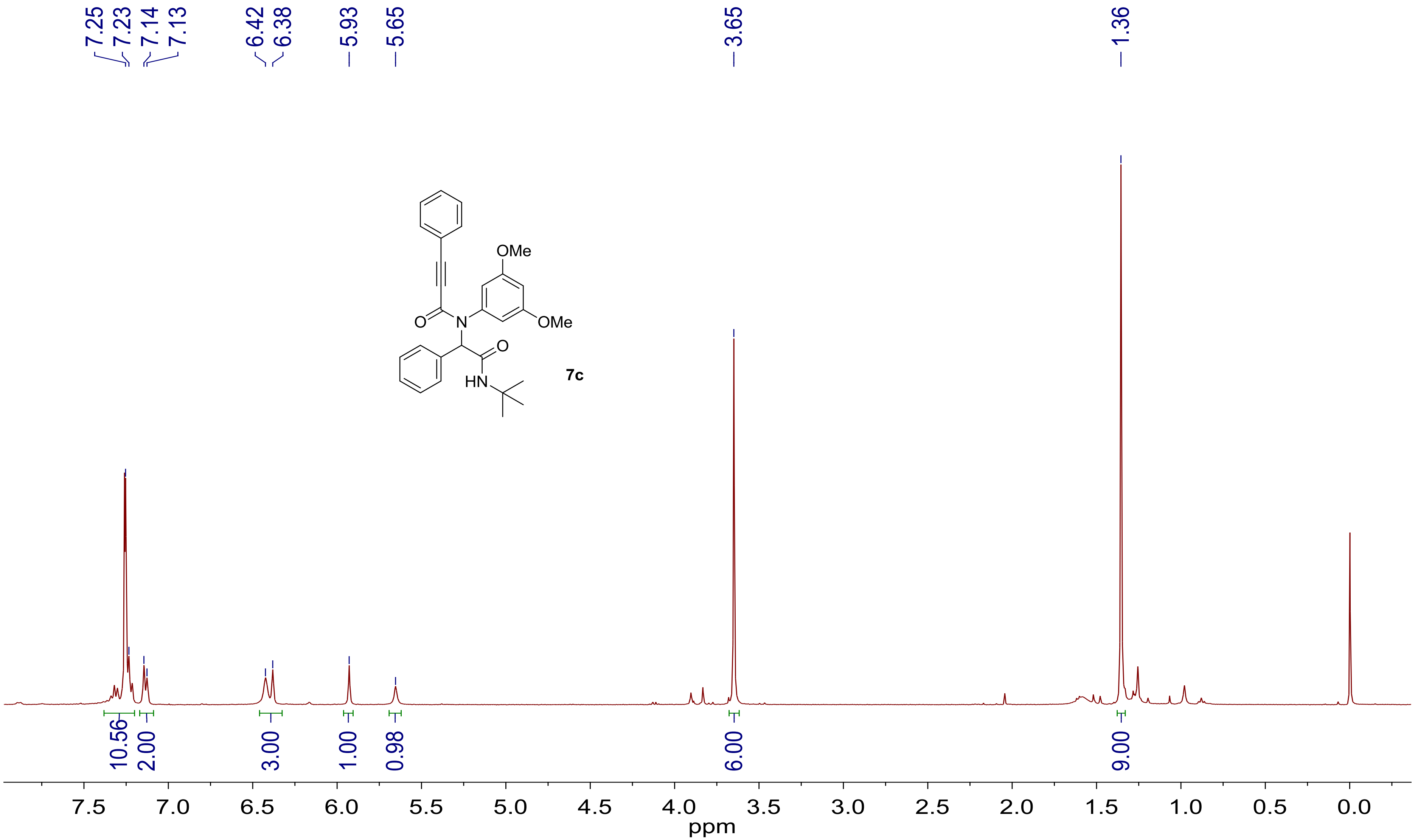
7.25
7.23
7.14
7.13
6.42
6.38
5.93
5.65
3.65
1.36
OMe
O
N
OMe
O
HN
7c
10.56
2.00
3.00
1.00
0.98
6.00
9.00
7.5
7.0
6.5
6.0
5.5
5.0
4.5
4.0
3.5
3.0
2.5
2.0
1.5
1.0
0.5
0.0
ppm

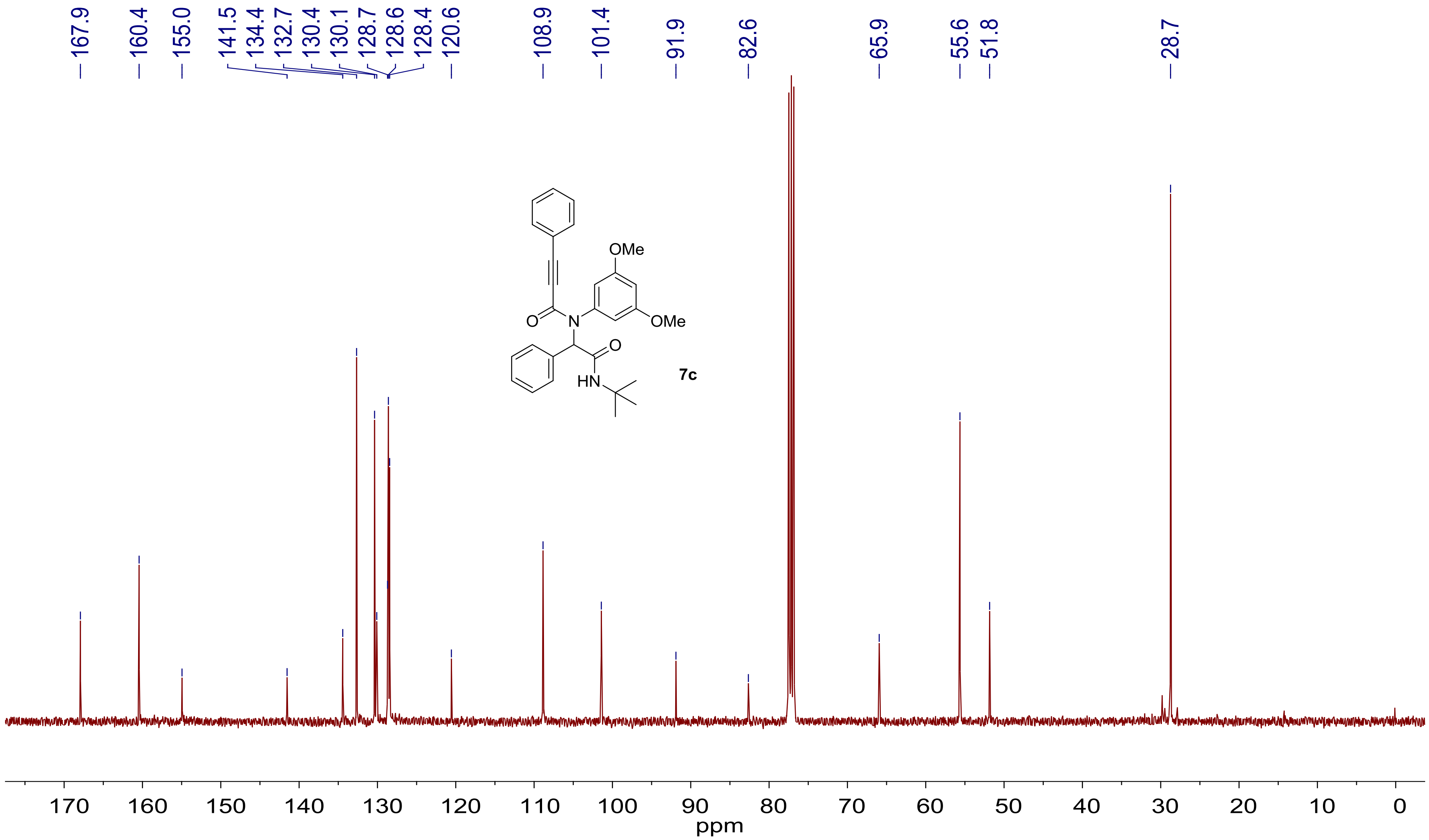
167.9
160.4
155.0
141.5
134.4
132.7
130.4
130.1
128.7
128.6
128.4
120.6
108.9
101.4
91.9
82.6
65.9
55.6
51.8
28.7
OMe
OMe
O
N
O
HN
7c
170
160
150
140
130
120
110
100
90
80
70
60
50
40
30
20
10
0
ppm

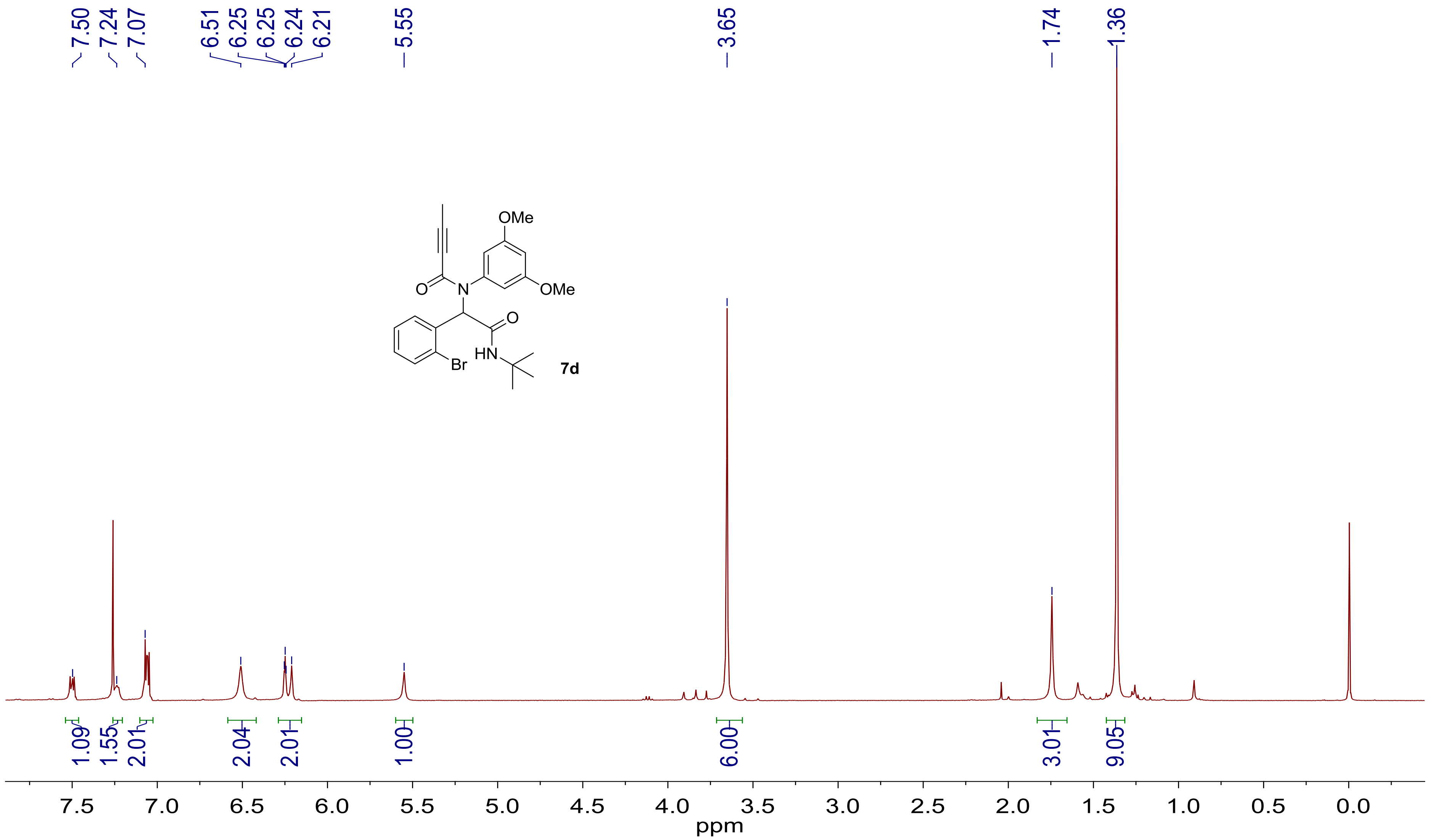

7.50
7.24
7.07
6.51
6.25
6.25
6.24
6.21
5.55
3.65
1.74
1.36
OMe
OMe
O
N
O
HN
Br
7d
1.09
1.55
2.01
2.04
2.01
1.00
6.00
3.01
9.05
7.5
7.0
6.5
6.0
5.5
5.0
4.5
4.0
3.5
3.0
2.5
2.0
1.5
1.0
0.5
0.0
ppm

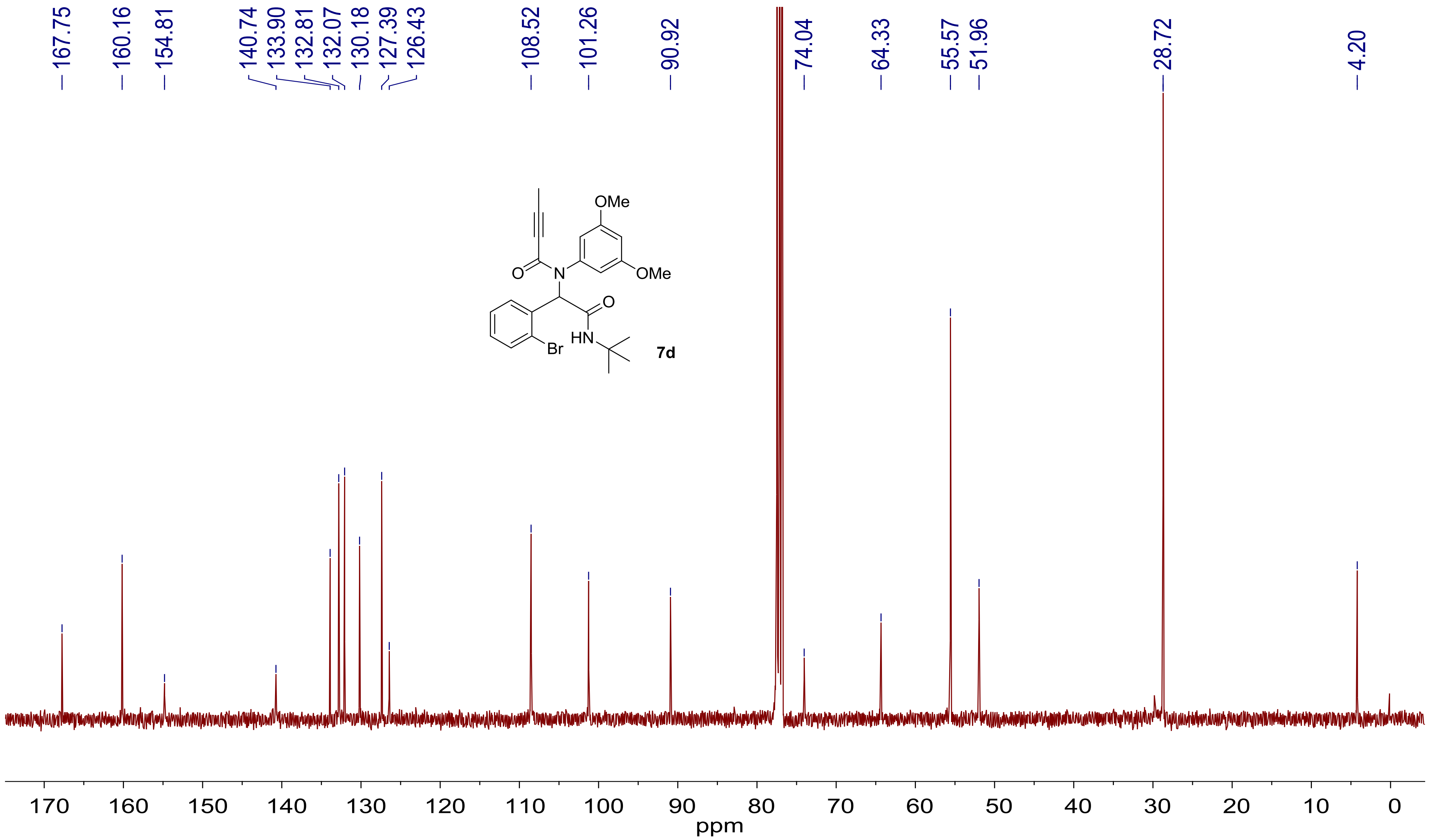
167.75
160.16
154.81
140.74
133.90
132.81
132.07
130.18
127.39
126.43
108.52
101.26
90.92
74.04
64.33
55.57
51.96
28.72
4.20
OMe
OMe
O
N
O
HN
Br
7d
170 160 150 140 130 120 110 100 90 80 70 60 50 40 30 20 10 0
ppm

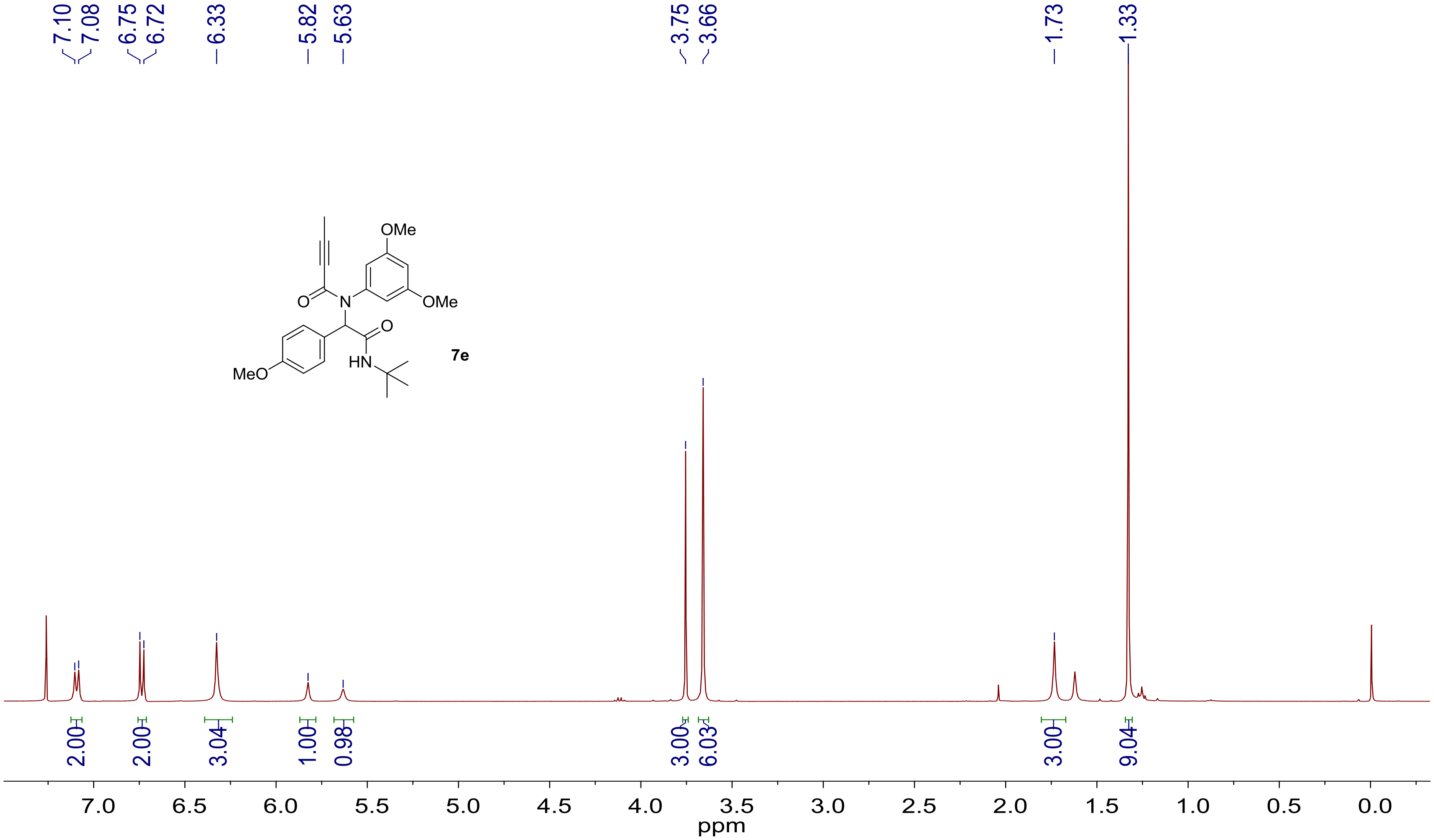
7.10
7.08
6.75
6.72
6.33
5.82
5.63
3.75
3.66
1.73
1.33
OMe
OMe
O
N
O
HN
MeO
7e
2.00
2.00
3.04
1.00
0.98
3.00
6.03
3.00
9.04
7.0
6.5
6.0
5.5
5.0
4.5
4.0
3.5
3.0
2.5
2.0
1.5
1.0
0.5
0.0
ppm

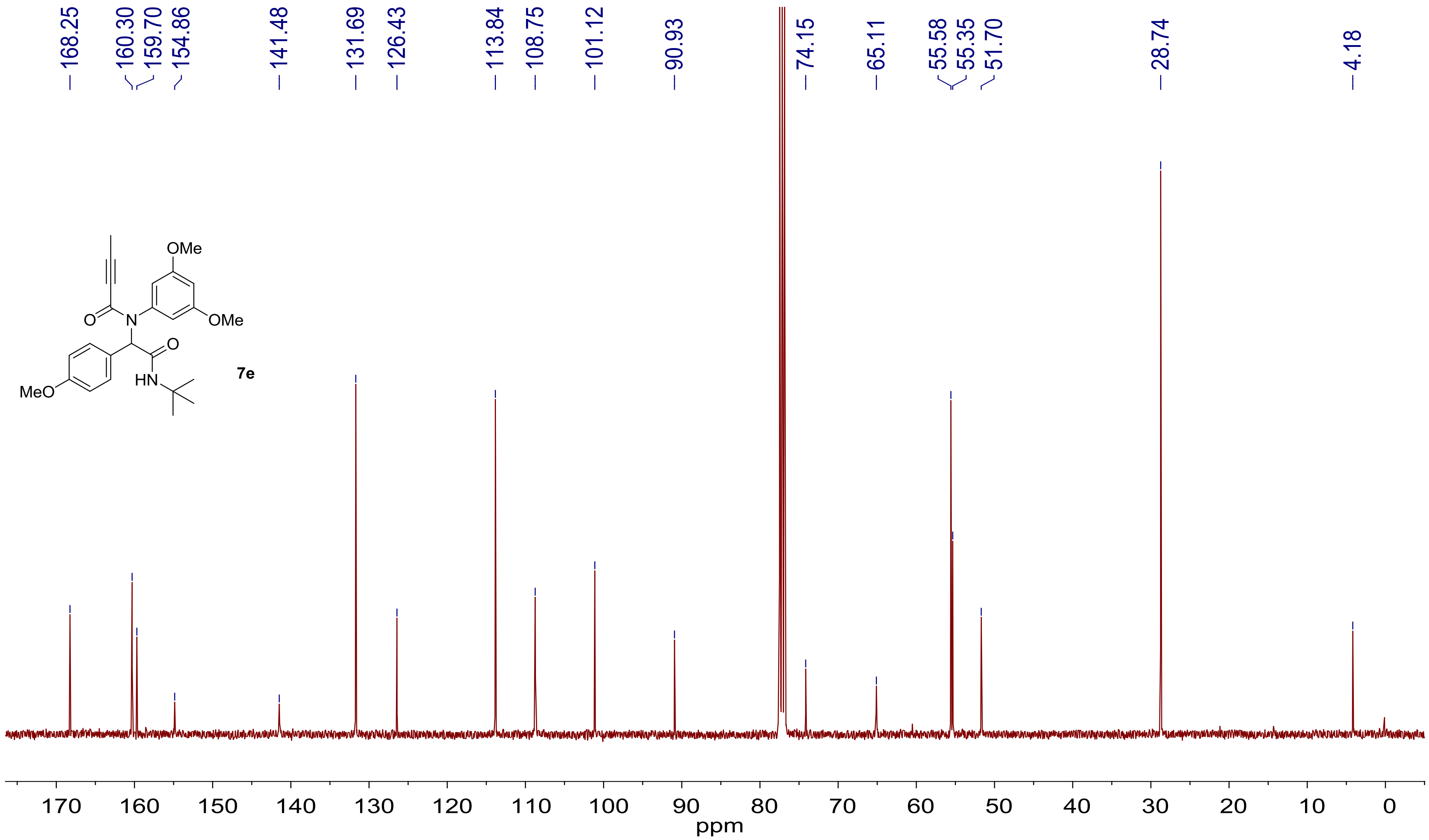
168.25
160.30
159.70
154.86
141.48
131.69
126.43
113.84
108.75
101.12
90.93
74.15
65.11
55.58
55.35
51.70
28.74
4.18
OMe
OMe
O
N
O
MeO
HN
7e
170 160 150 140 130 120 110 100 90 80 70 60 50 40 30 20 10 0
ppm

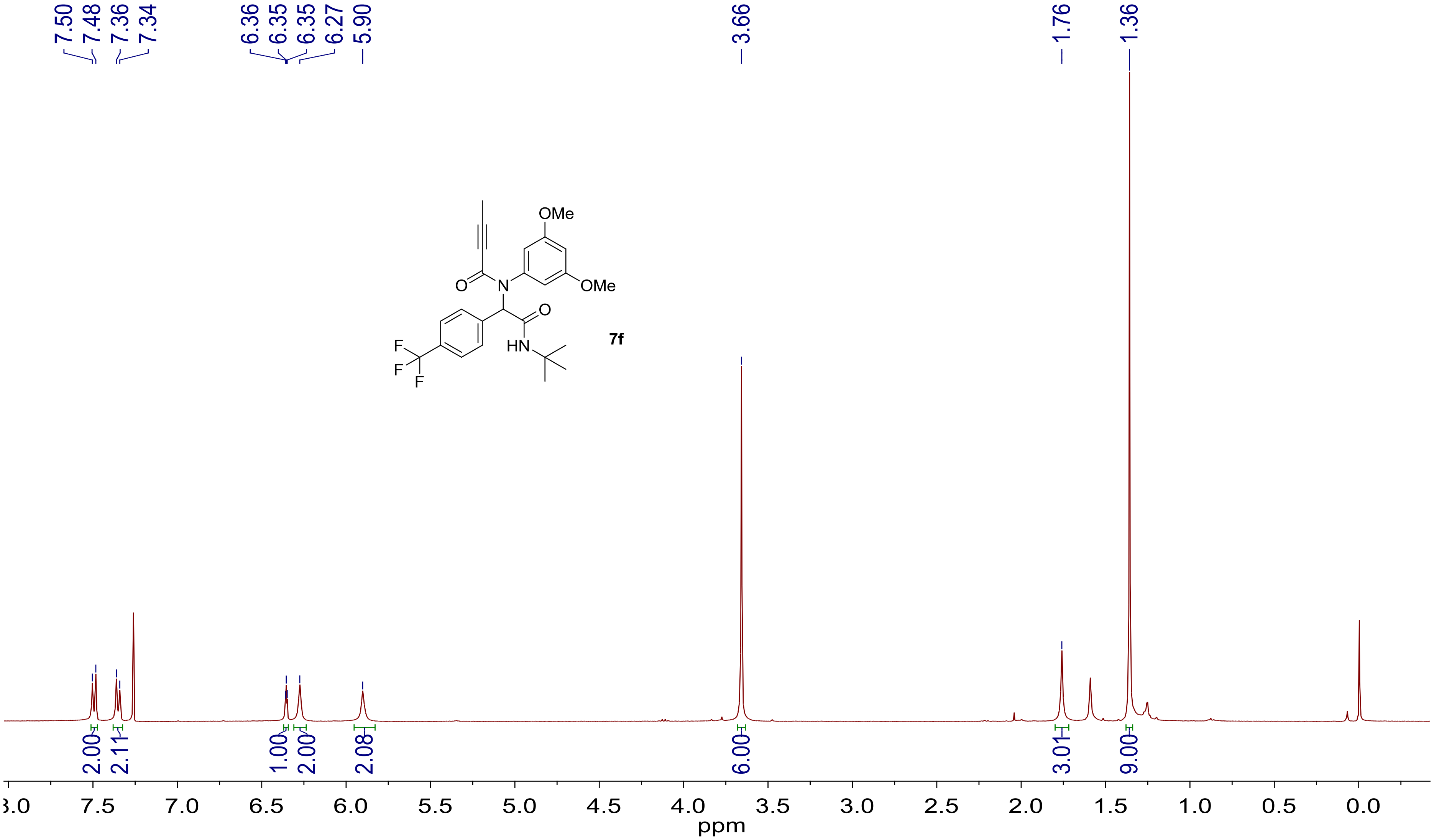
7.50
7.48
7.36
7.34
6.36
6.35
6.35
6.27
5.90
3.66
1.76
1.36
OMe
OMe
O
N
O
HN
F
F
F
7f
2.00
2.11
1.00
2.00
2.08
6.00
3.01
9.00
8.0
7.5
7.0
6.5
6.0
5.5
5.0
4.5
4.0
3.5
3.0
2.5
2.0
1.5
1.0
0.5
0.0
ppm

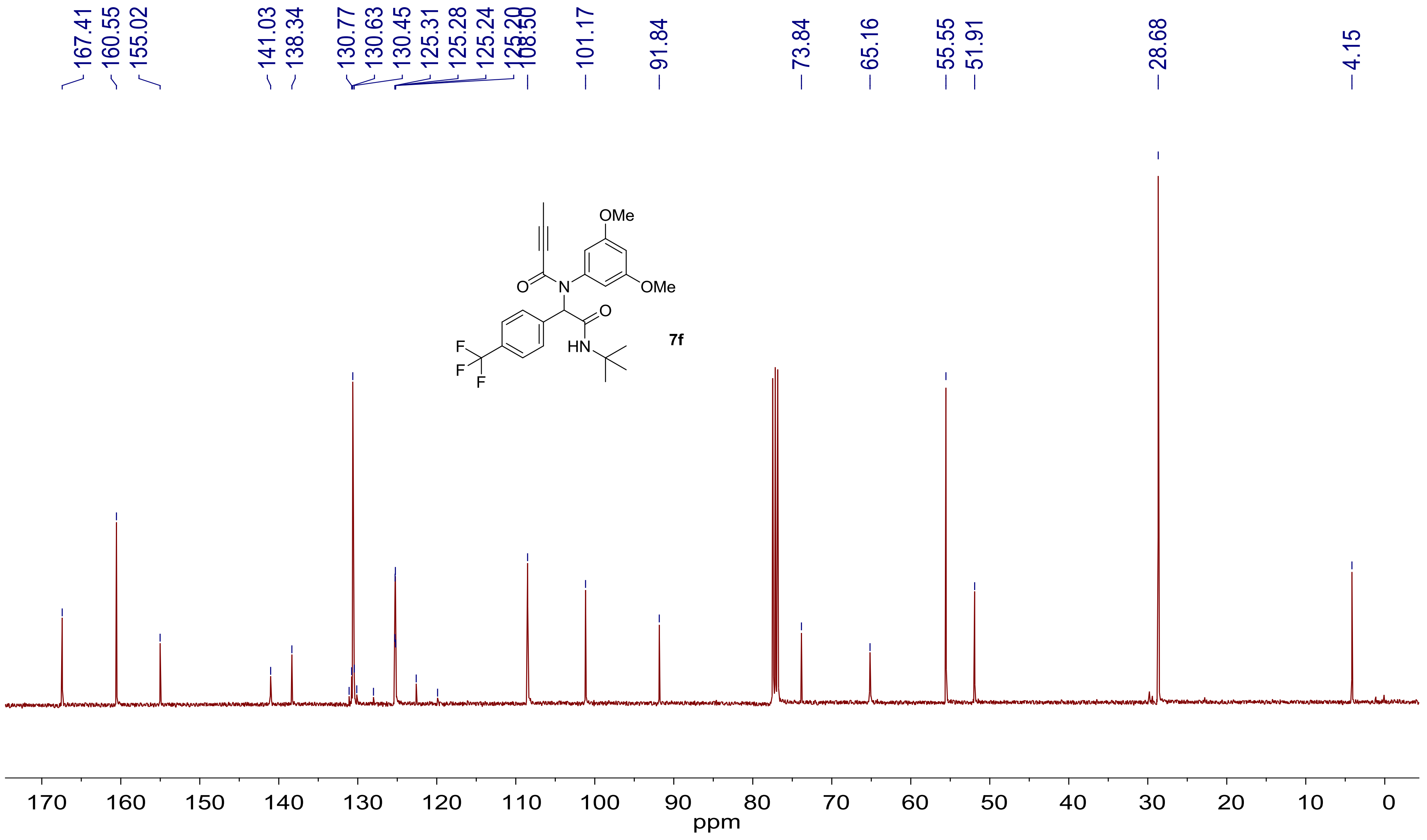
167.41
160.55
155.02
141.03
138.34
130.77
130.63
130.45
125.31
125.28
125.24
125.20
108.50
101.17
91.84
73.84
65.16
55.55
51.91
28.68
4.15
OMe
OMe
O
N
O
HN
F
F
F
7f
170
160
150
140
130
120
110
100
90
80
70
60
50
40
30
20
10
0
ppm

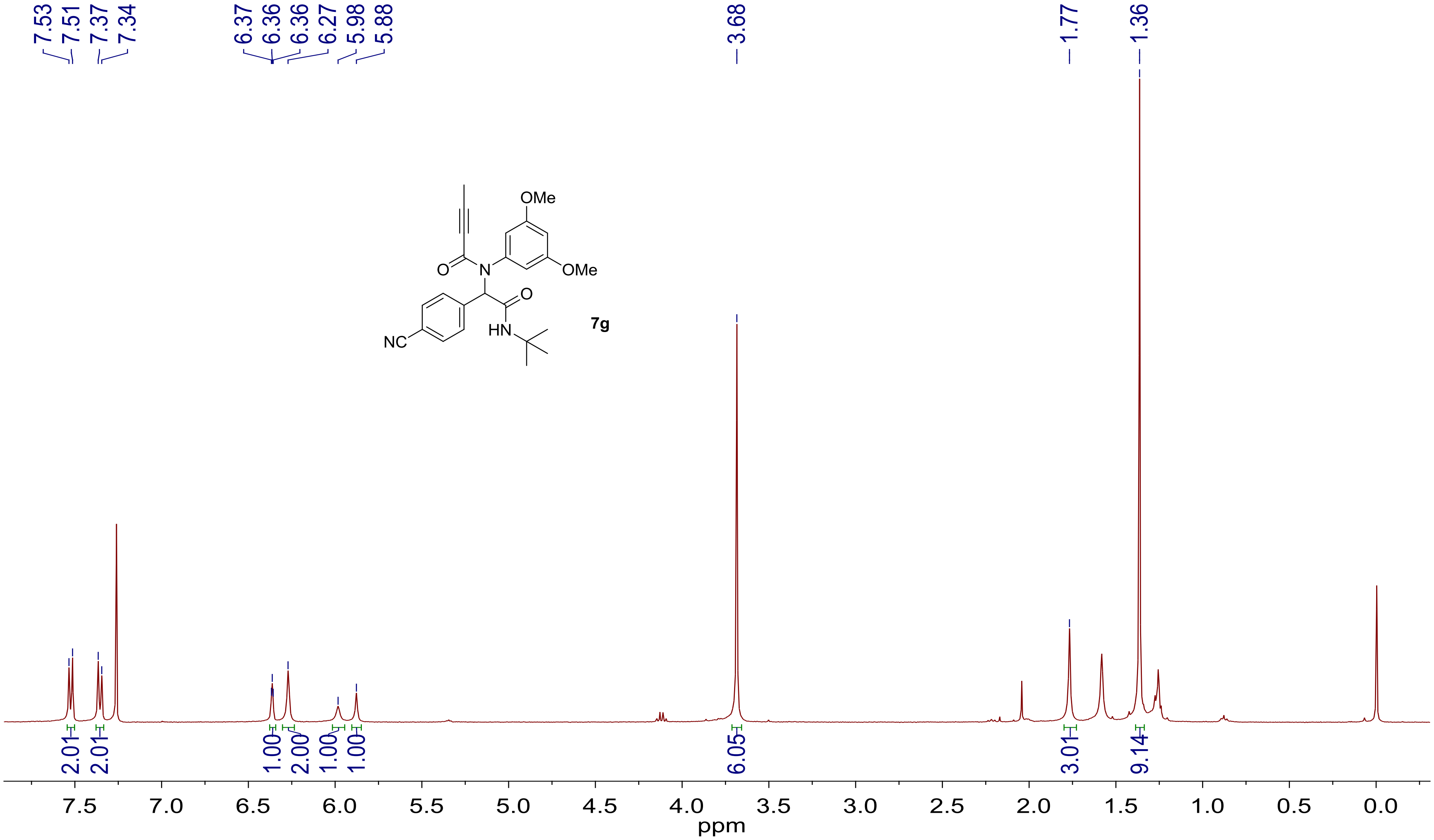
7.53
7.51
7.37
7.34
6.37
6.36
6.36
6.27
5.98
5.88
3.68
1.77
1.36
OMe
OMe
O
N
O
HN
NC
7g
2.01
2.01
1.00
2.00
1.00
1.00
6.05
3.01
9.14
7.5
7.0
6.5
6.0
5.5
5.0
4.5
4.0
3.5
3.0
2.5
2.0
1.5
1.0
0.5
0.0
ppm

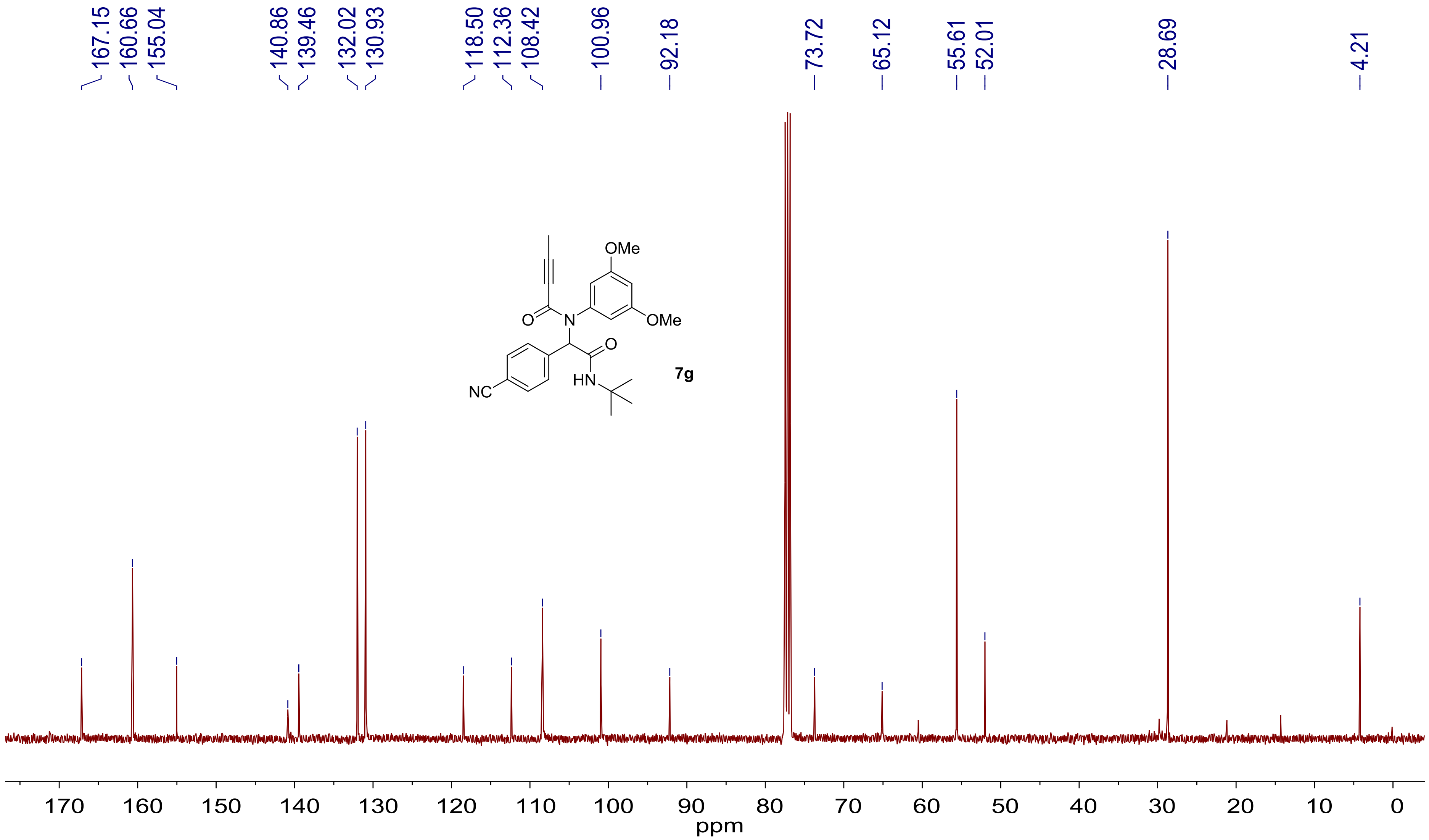
167.15
160.66
155.04
140.86
139.46
132.02
130.93
118.50
112.36
108.42
100.96
92.18
73.72
65.12
55.61
52.01
28.69
4.21
OMe
OMe
O
N
O
HN
NC
7g
170 160 150 140 130 120 110 100 90 80 70 60 50 40 30 20 10 0
ppm

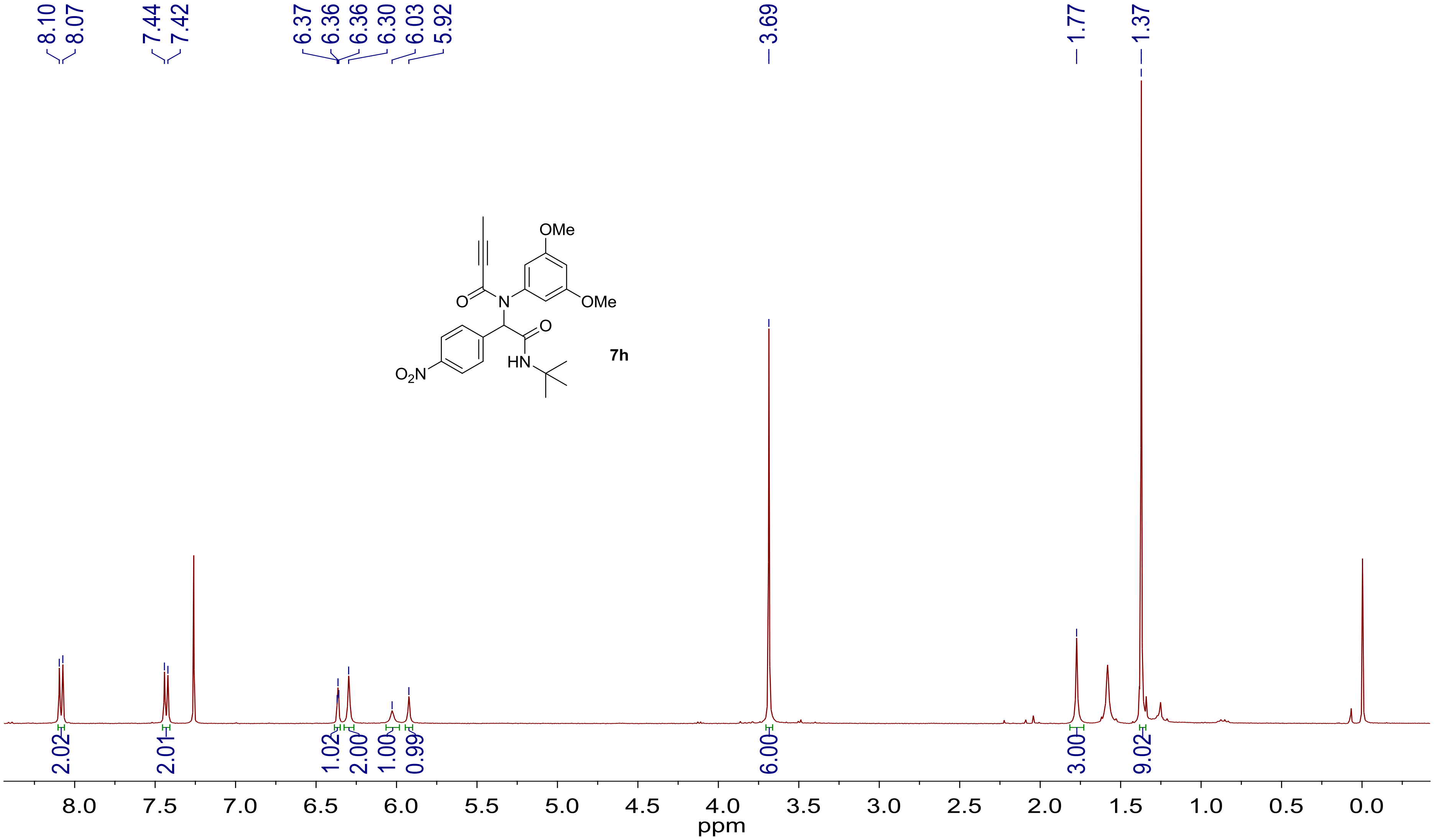
8.10
8.07
7.44
7.42
6.37
6.36
6.36
6.30
6.03
5.92
3.69
1.77
1.37
OMe
OMe
O
N
O
HN
O2N
2.02
2.01
1.02
2.00
1.00
0.99
6.00
3.00
9.02
8.0
7.5
7.0
6.5
6.0
5.5
5.0
4.5
4.0
3.5
3.0
2.5
2.0
1.5
1.0
0.5
0.0
ppm

7h

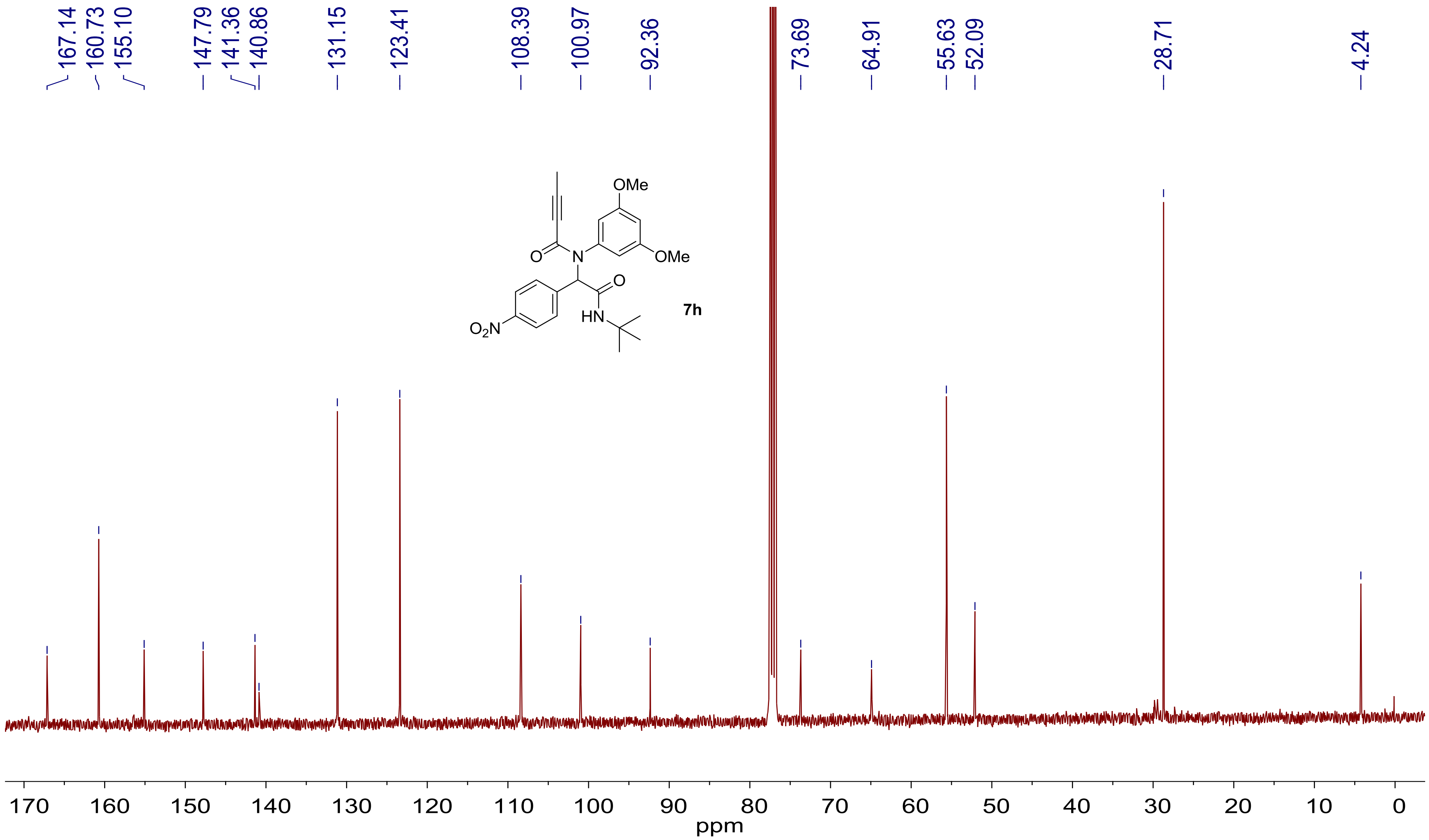
167.14
160.73
155.10
147.79
141.36
140.86
131.15
123.41
108.39
100.97
92.36
73.69
64.91
55.63
52.09
28.71
4.24
OMe
OMe
O
N
O
O_2N
HN
7h
170 160 150 140 130 120 110 100 90 80 70 60 50 40 30 20 10 0
ppm

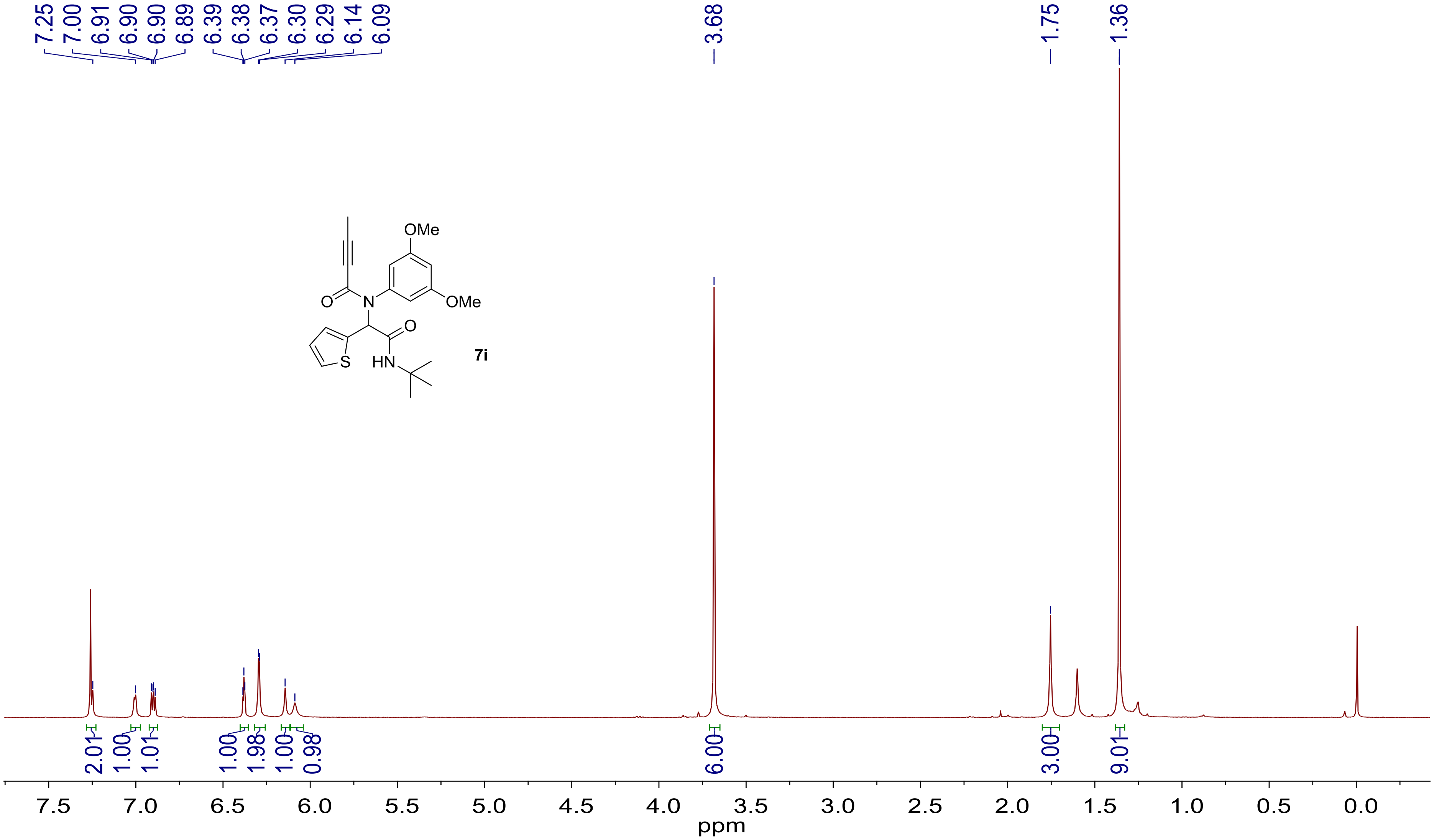

7.25
7.00
6.91
6.90
6.90
6.89
6.39
6.38
6.37
6.30
6.29
6.14
6.09
3.68
1.75
1.36
OMe
OMe
O
N
O
S
HN
2.01
1.00
1.01
1.00
1.98
1.00
0.98
6.00
3.00
9.01
7.5
7.0
6.5
6.0
5.5
5.0
4.5
4.0
3.5
3.0
2.5
2.0
1.5
1.0
0.5
0.0
ppm

7i

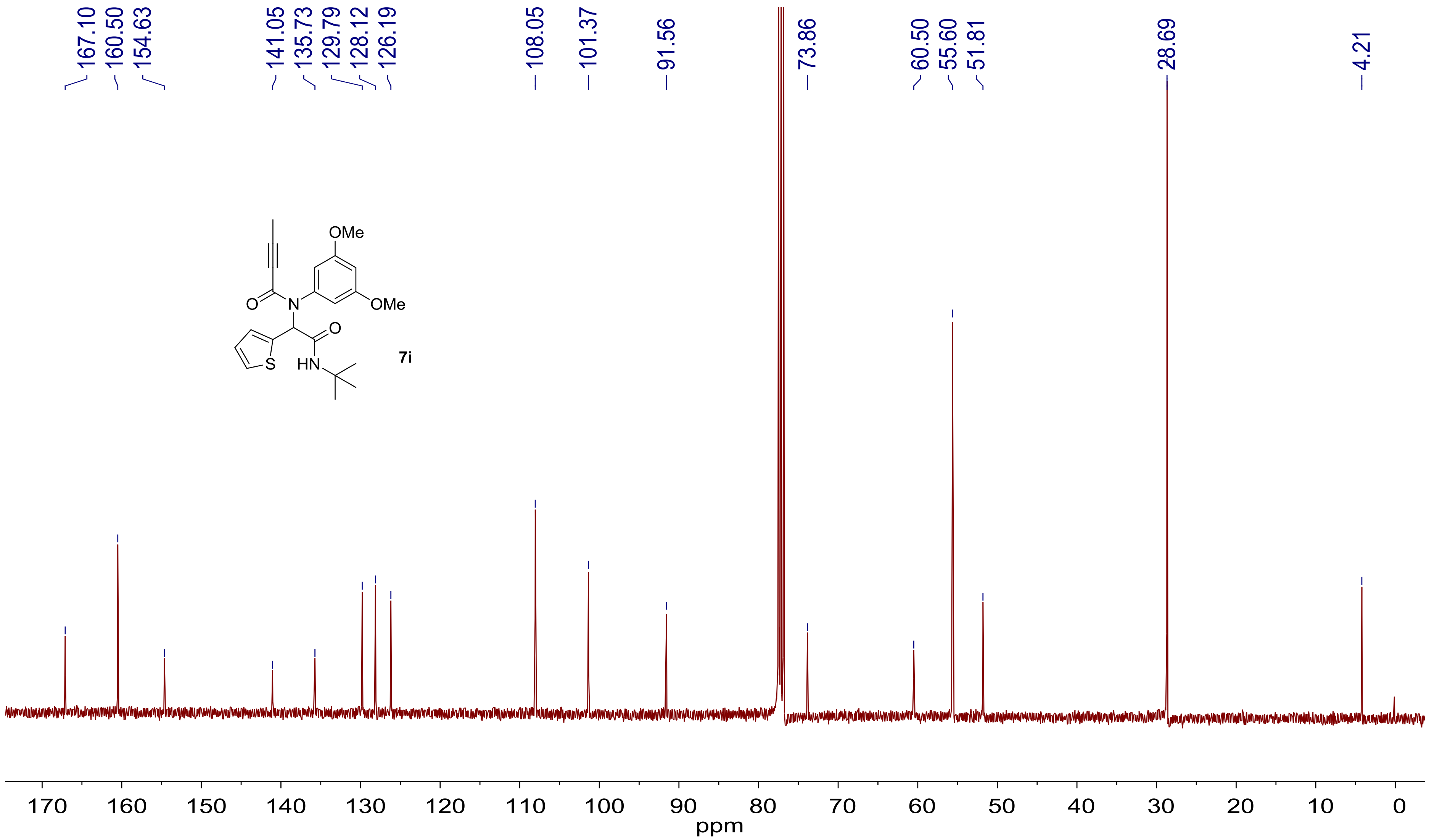
167.10
160.50
154.63
141.05
135.73
129.79
128.12
126.19
108.05
101.37
91.56
73.86
60.50
55.60
51.81
28.69
4.21
OMe
OMe
O
N
O
S
HN
7i
170 160 150 140 130 120 110 100 90 80 70 60 50 40 30 20 10 0
ppm

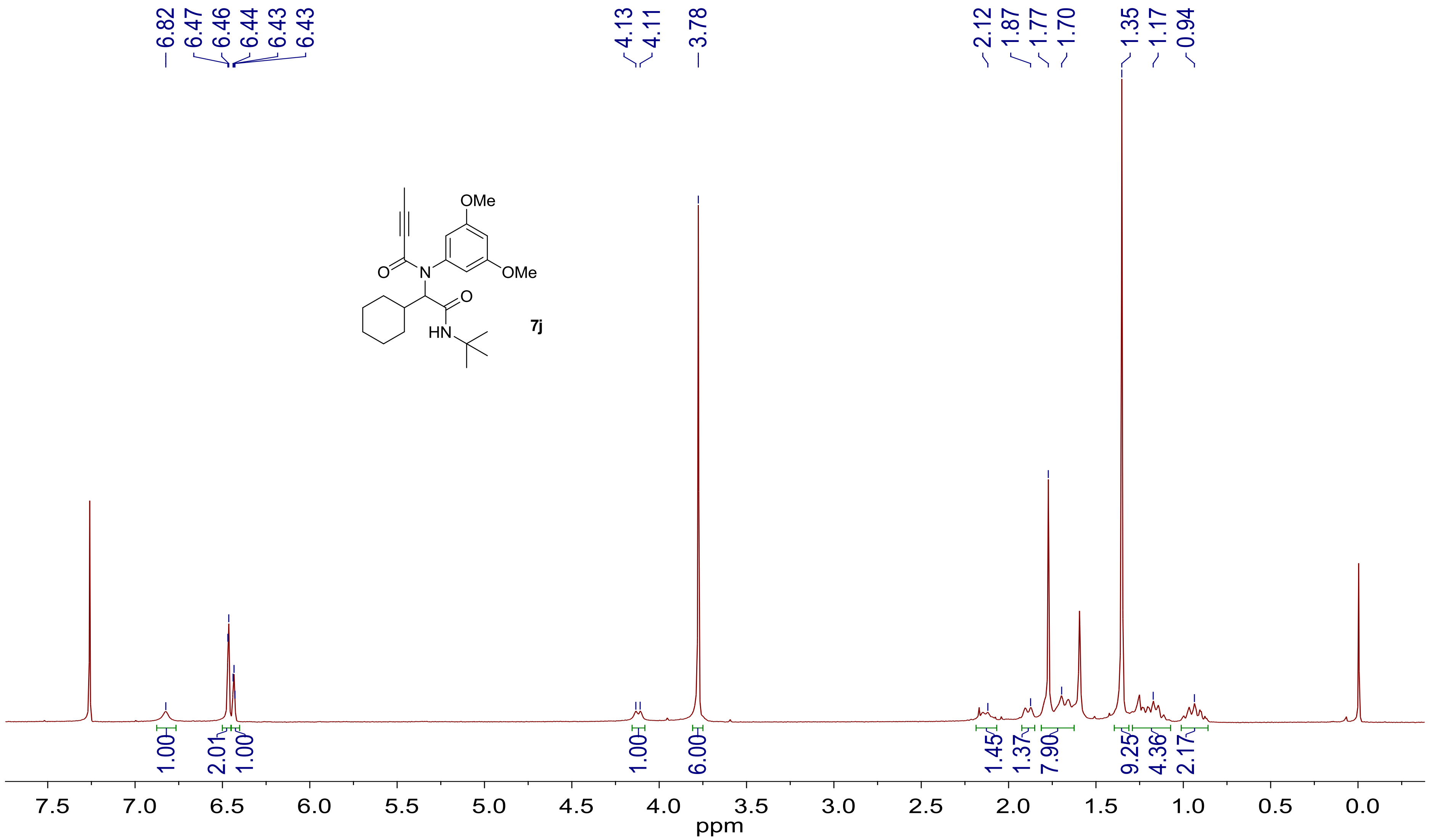

6.82
6.47
6.46
6.44
6.43
6.43
4.13
4.11
3.78
2.12
1.87
1.77
1.70
1.35
1.17
0.94
OMe
OMe
O
N
O
HN
7j
1.00
2.01
1.00
1.00
6.00
1.45
1.37
7.90
9.25
4.36
2.17
7.5
7.0
6.5
6.0
5.5
5.0
4.5
4.0
3.5
3.0
2.5
2.0
1.5
1.0
0.5
0.0
ppm

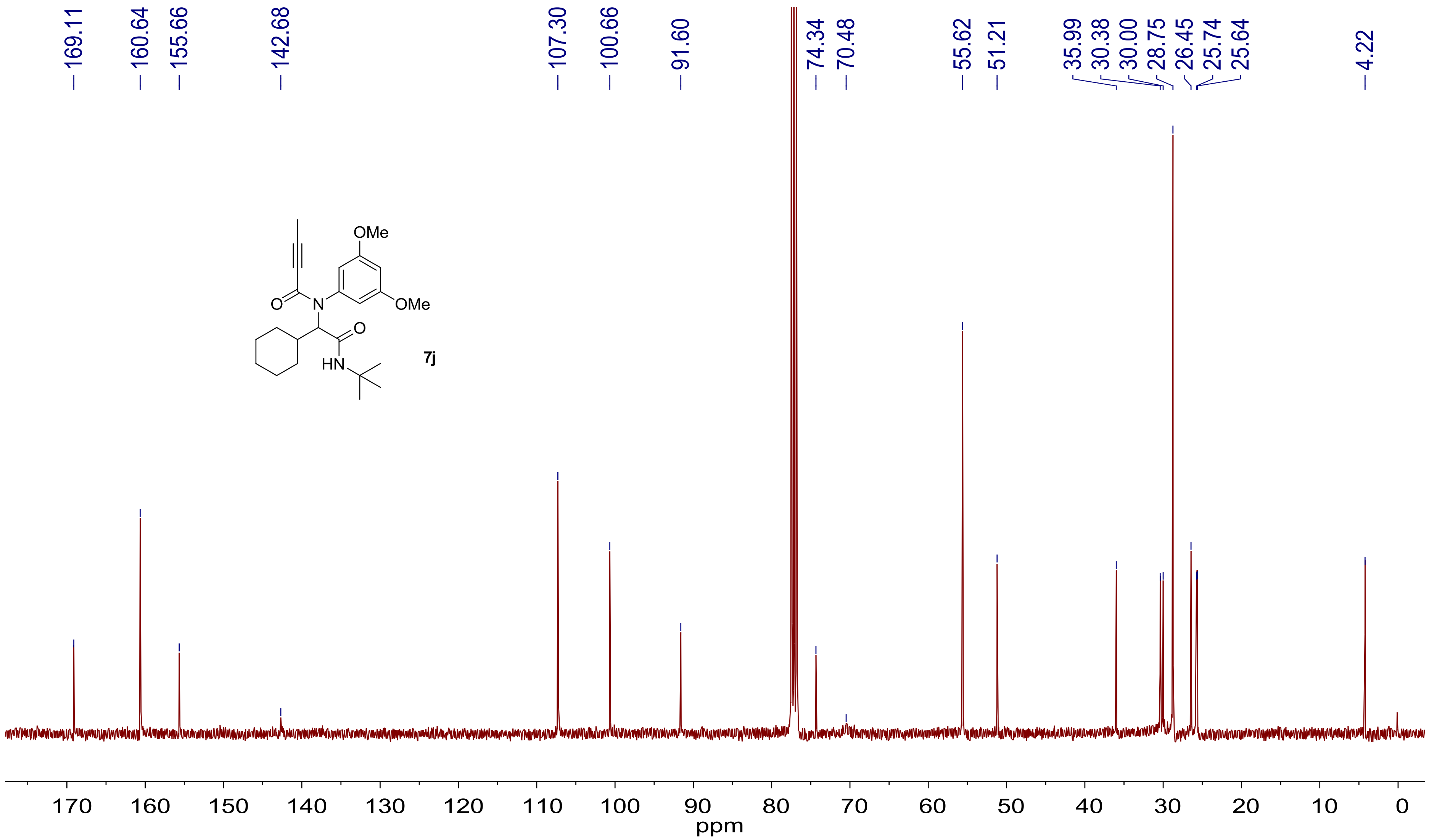
169.11
160.64
155.66
142.68
107.30
100.66
91.60
74.34
70.48
55.62
51.21
35.99
30.38
30.00
28.75
26.45
25.74
25.64
4.22
OMe
OMe
O
N
O
HN
7j
170 160 150 140 130 120 110 100 90 80 70 60 50 40 30 20 10 0
ppm

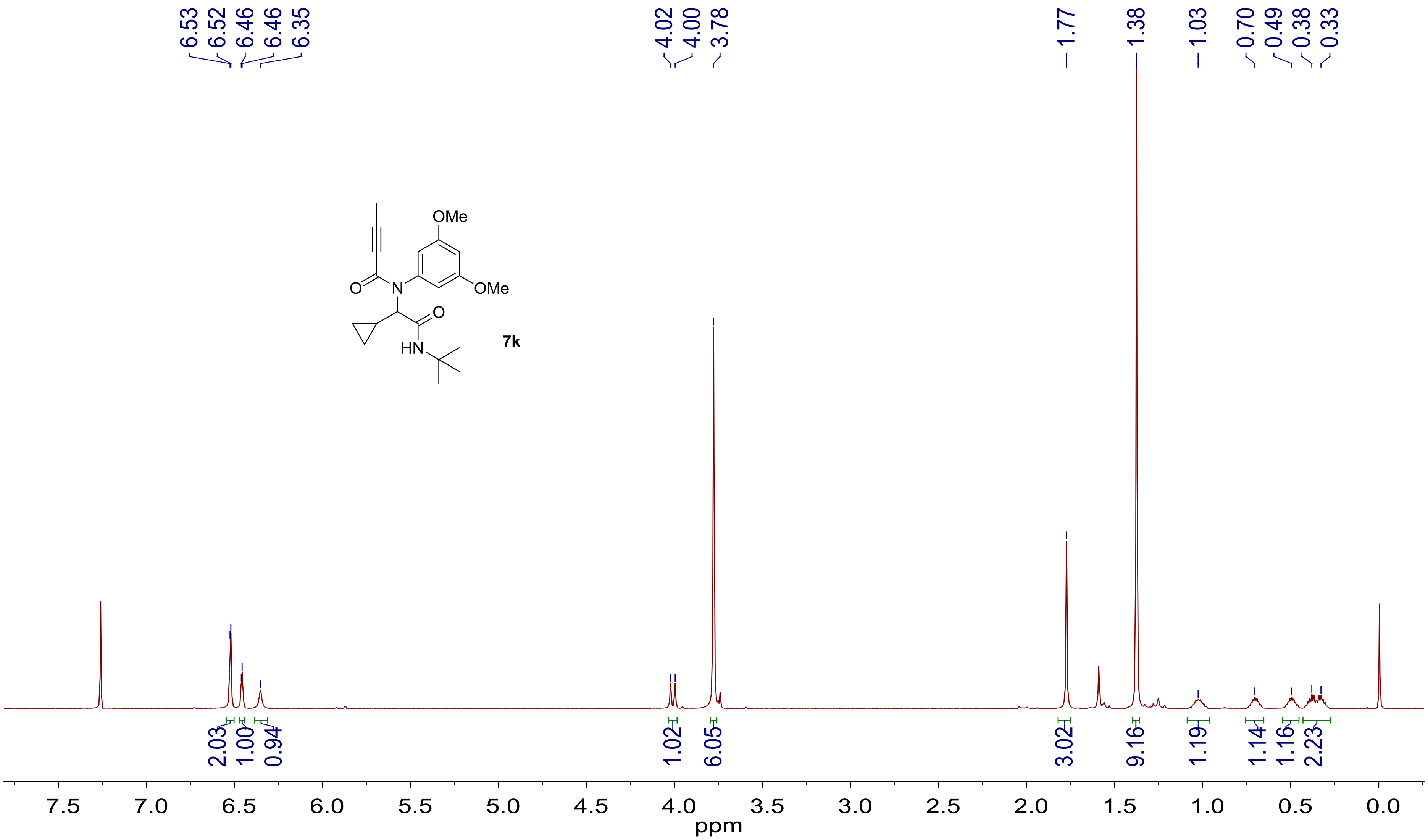
6.53
6.52
6.46
6.46
6.35
4.02
4.00
3.78
1.77
1.38
1.03
0.70
0.49
0.38
0.33
OMe
OMe
O
N
O
HN
7k
2.03
1.00
0.94
1.02
6.05
3.02
9.16
1.19
1.14
1.16
2.23
7.5
7.0
6.5
6.0
5.5
5.0
4.5
4.0
3.5
3.0
2.5
2.0
1.5
1.0
0.5
0.0
ppm

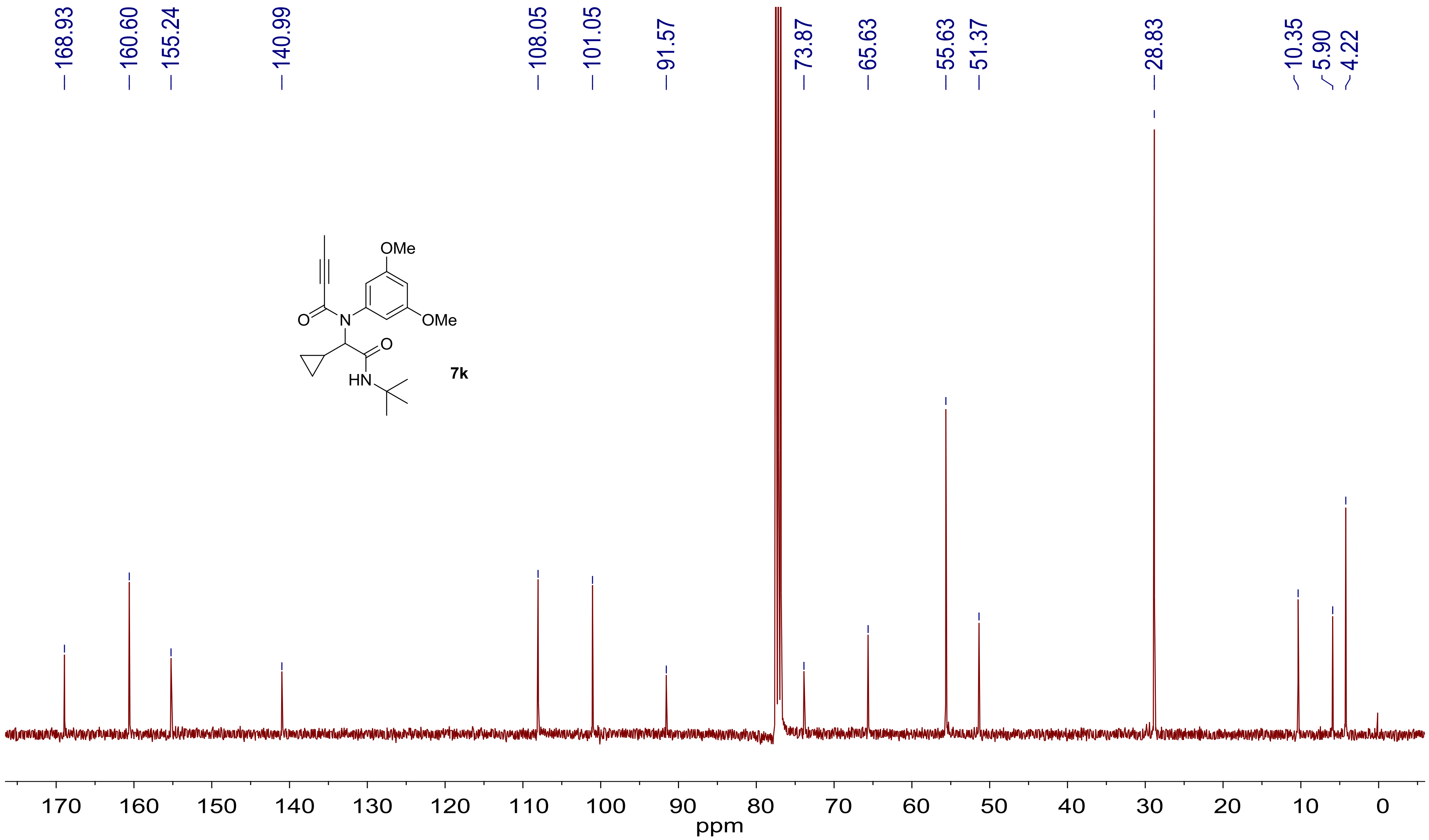
168.93
160.60
155.24
140.99
108.05
101.05
91.57
73.87
65.63
55.63
51.37
28.83
10.35
5.90
4.22
OMe
OMe
O
N
O
HN
7k
170
160
150
140
130
120
110
100
90
80
70
60
50
40
30
20
10
0
ppm

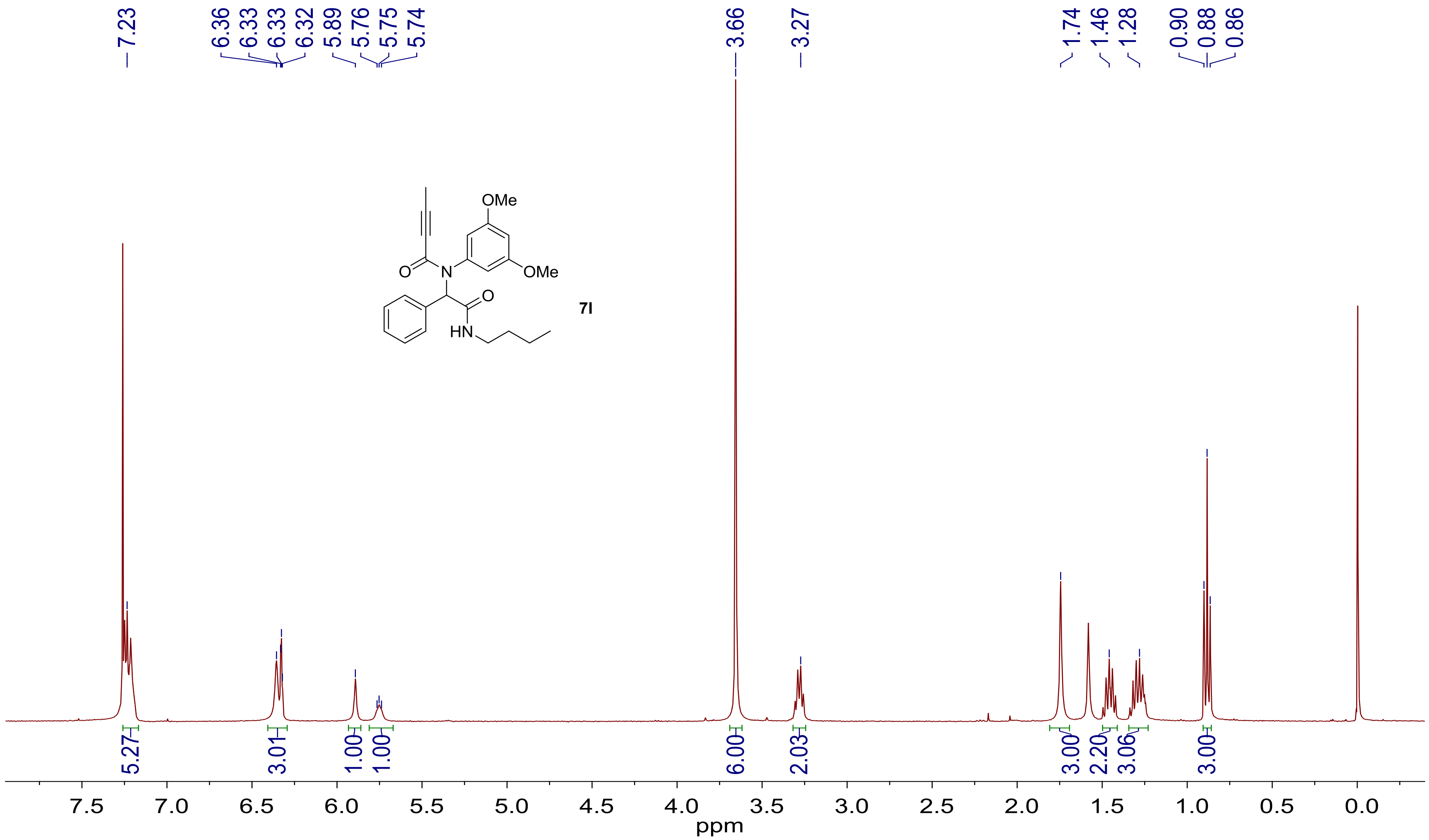
7.23
6.36
6.33
6.33
6.32
5.89
5.76
5.75
5.74
3.66
3.27
1.74
1.46
1.28
0.90
0.88
0.86
OMe
OMe
O
N
O
HN
7l
5.27
3.01
1.00
1.00
6.00
2.03
3.00
2.20
3.06
3.00
7.5
7.0
6.5
6.0
5.5
5.0
4.5
4.0
3.5
3.0
2.5
2.0
1.5
1.0
0.5
0.0
ppm

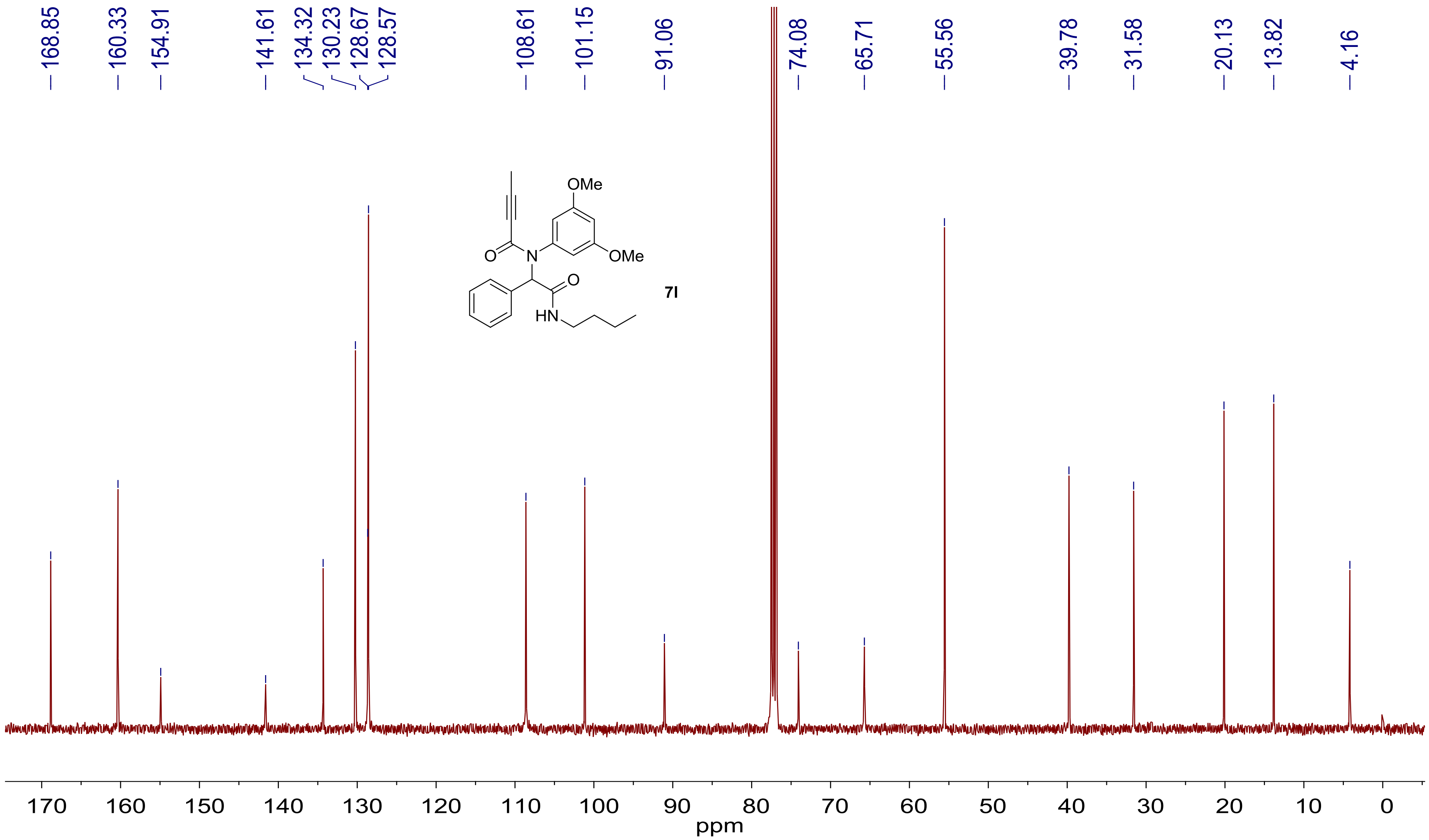
168.85
160.33
154.91
141.61
134.32
130.23
128.67
128.57
108.61
101.15
91.06
74.08
65.71
55.56
39.78
31.58
20.13
13.82
4.16
OMe
OMe
O
N
O
HN
7l
170
160
150
140
130
120
110
100
90
80
70
60
50
40
30
20
10
0
ppm

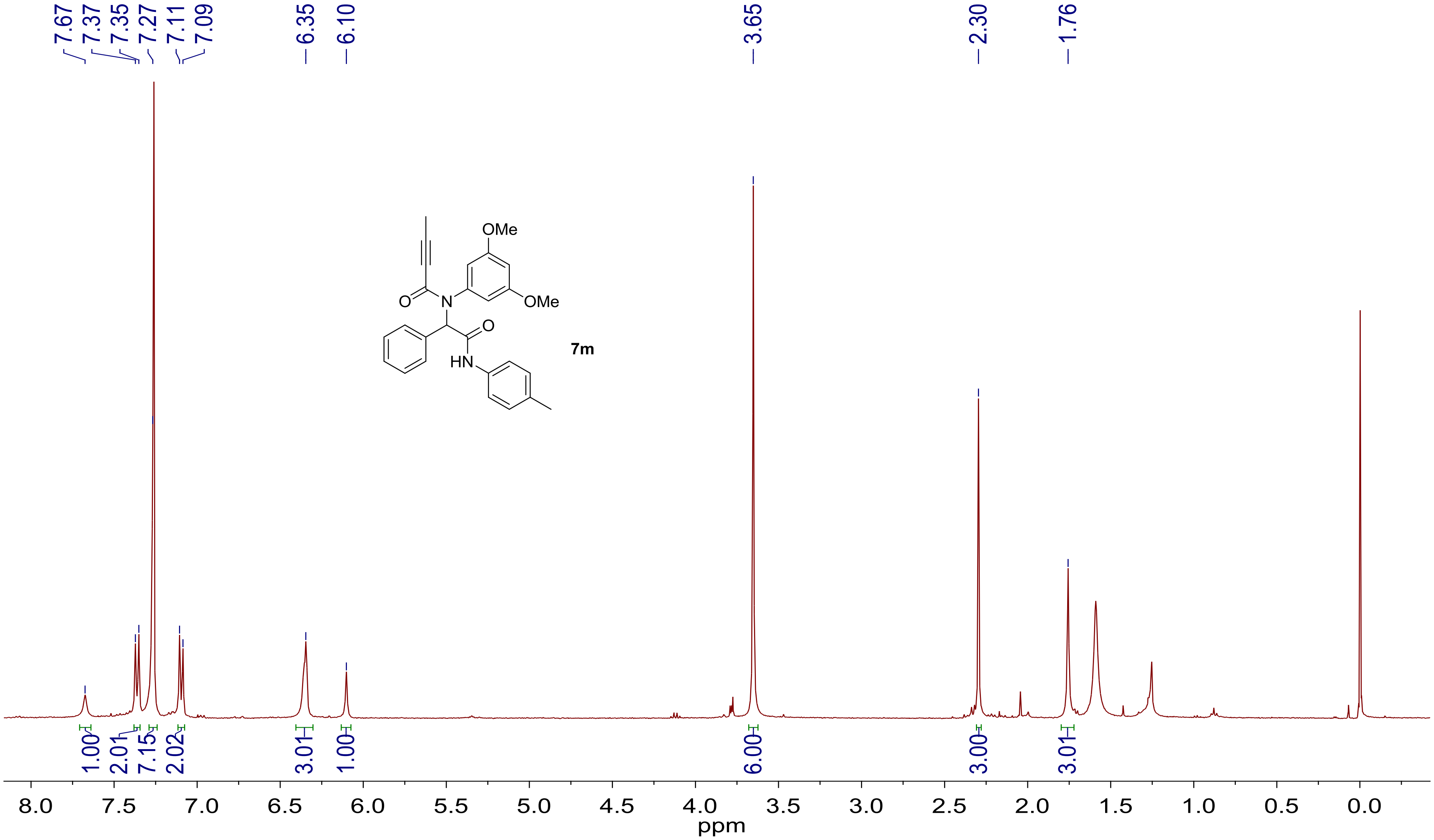
7.67
7.37
7.35
7.27
7.11
7.09
6.35
6.10
3.65
2.30
1.76
OMe
OMe
N
O
O
HN
7m
1.00
2.01
7.15
2.02
3.01
1.00
6.00
3.00
3.01
8.0
7.5
7.0
6.5
6.0
5.5
5.0
4.5
4.0
3.5
3.0
2.5
2.0
1.5
1.0
0.5
0.0
ppm

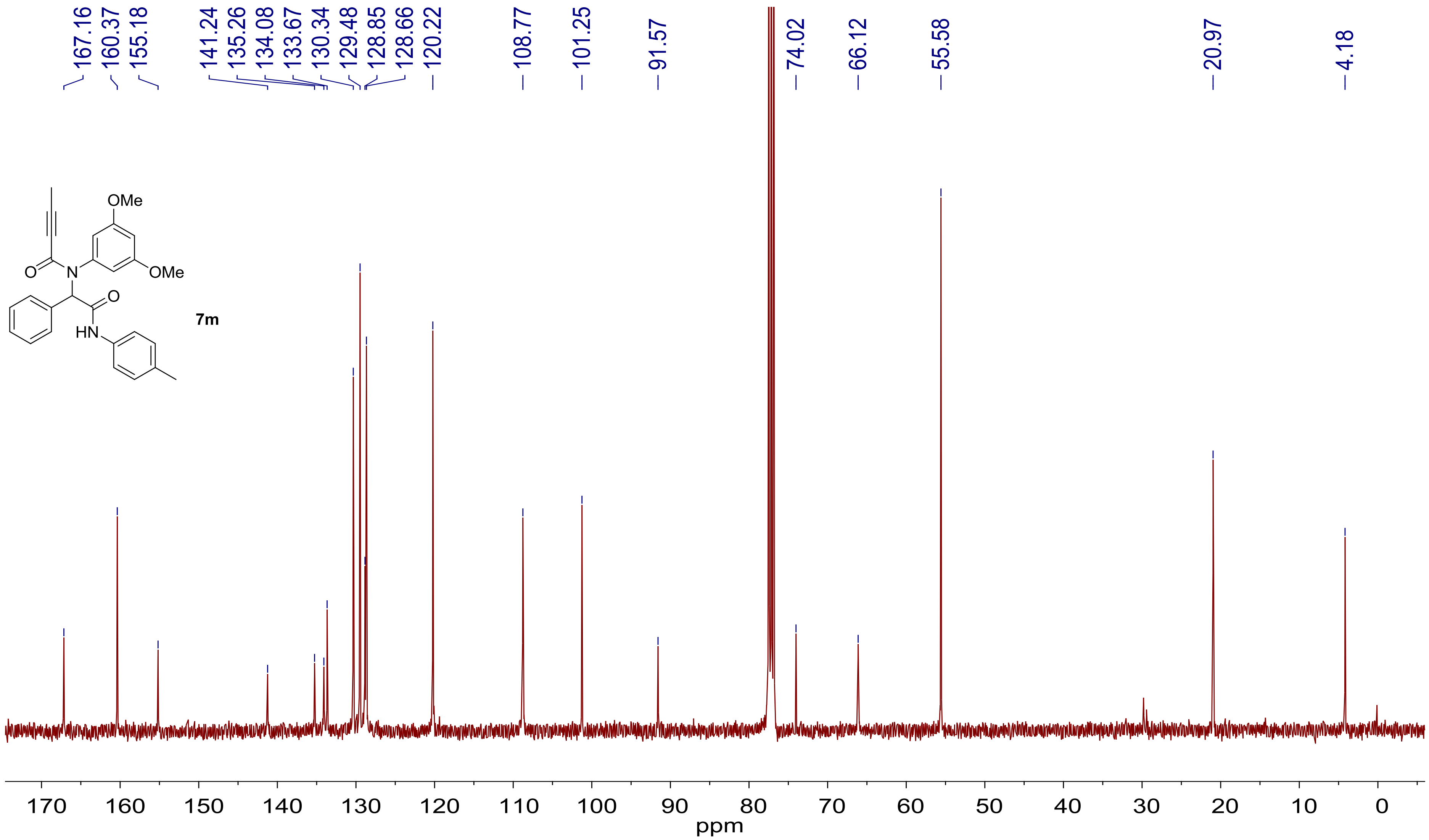
167.16
160.37
155.18
141.24
135.26
134.08
133.67
130.34
129.48
128.85
128.66
120.22
108.77
101.25
91.57
74.02
66.12
55.58
20.97
4.18
OMe
O
N
OMe
O
HN
7m
170
160
150
140
130
120
110
100
90
80
70
60
50
40
30
20
10
0
ppm

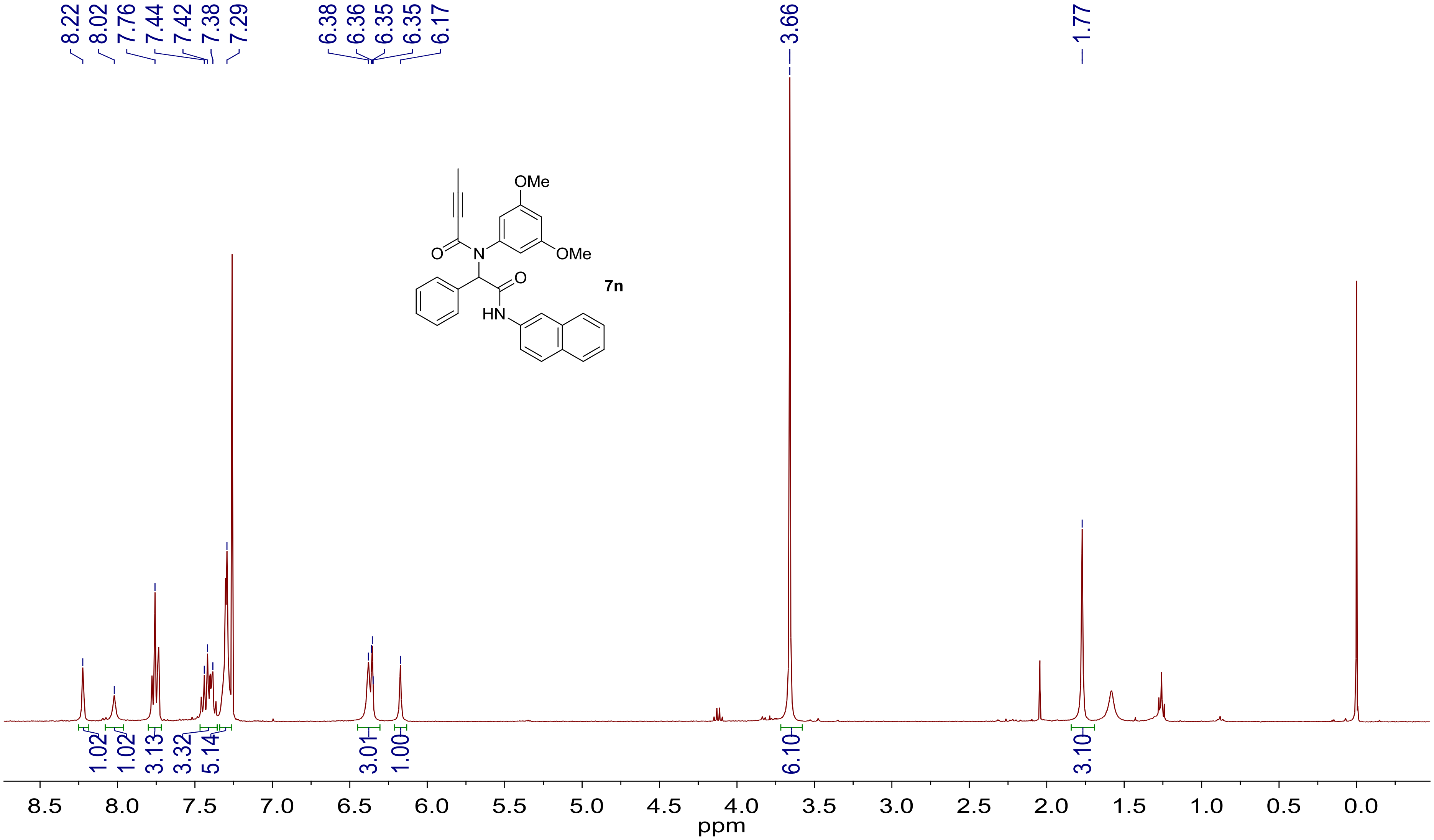
8.22
8.02
7.76
7.44
7.42
7.38
7.29
6.38
6.36
6.35
6.35
6.17
3.66
1.77
OMe
O
N
OMe
O
HN
7n
1.02
1.02
3.13
3.32
5.14
3.01
1.00
6.10
3.10
8.5 8.0 7.5 7.0 6.5 6.0 5.5 5.0 4.5 4.0 3.5 3.0 2.5 2.0 1.5 1.0 0.5 0.0
ppm

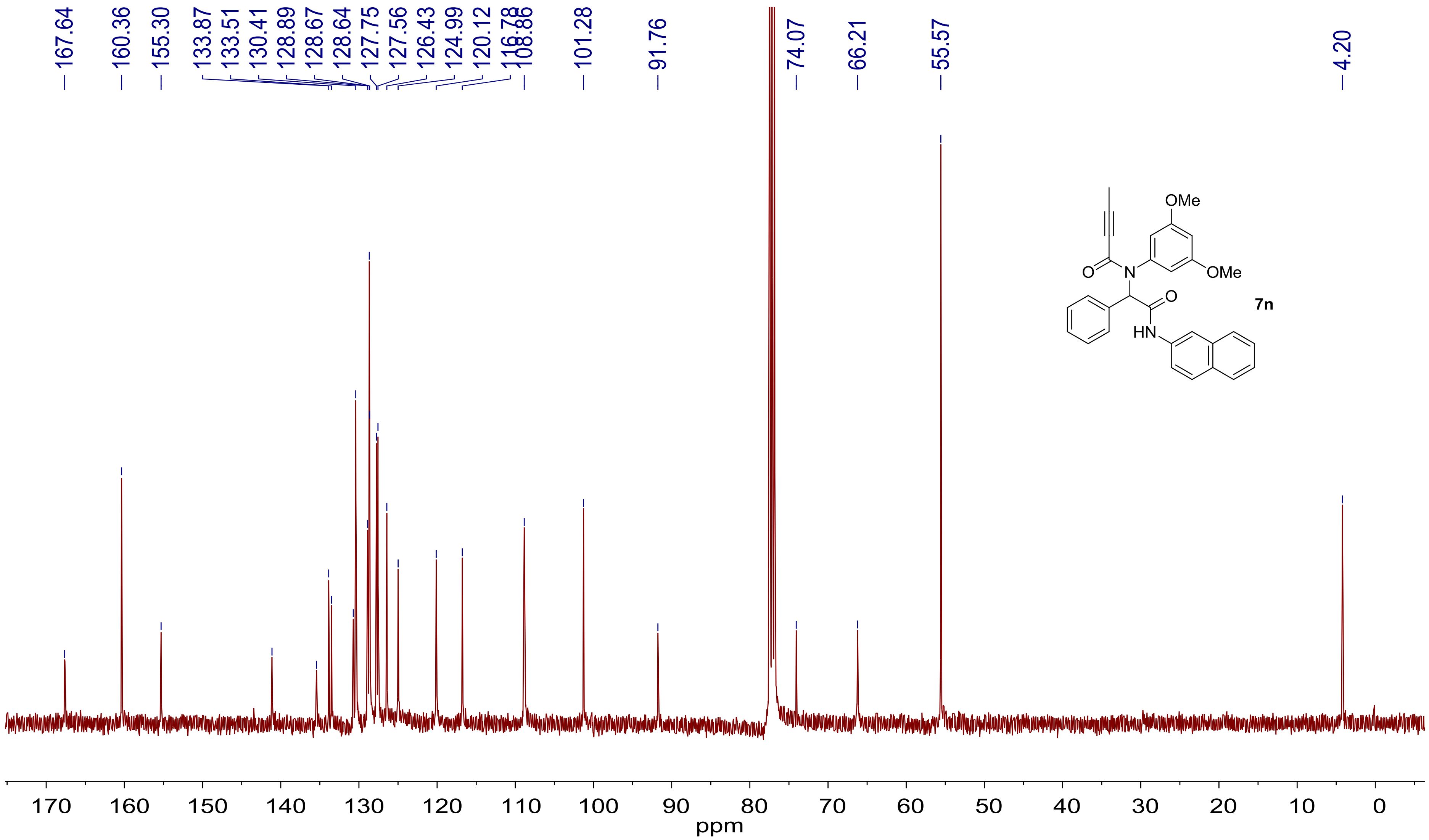

167.64
160.36
155.30
133.87
133.51
130.41
128.89
128.67
128.64
127.75
127.56
126.43
124.99
120.12
116.78
108.86
101.28
91.76
74.07
66.21
55.57
4.20
OMe
OMe
O
N
O
HN
7n
170 160 150 140 130 120 110 100 90 80 70 60 50 40 30 20 10 0
ppm

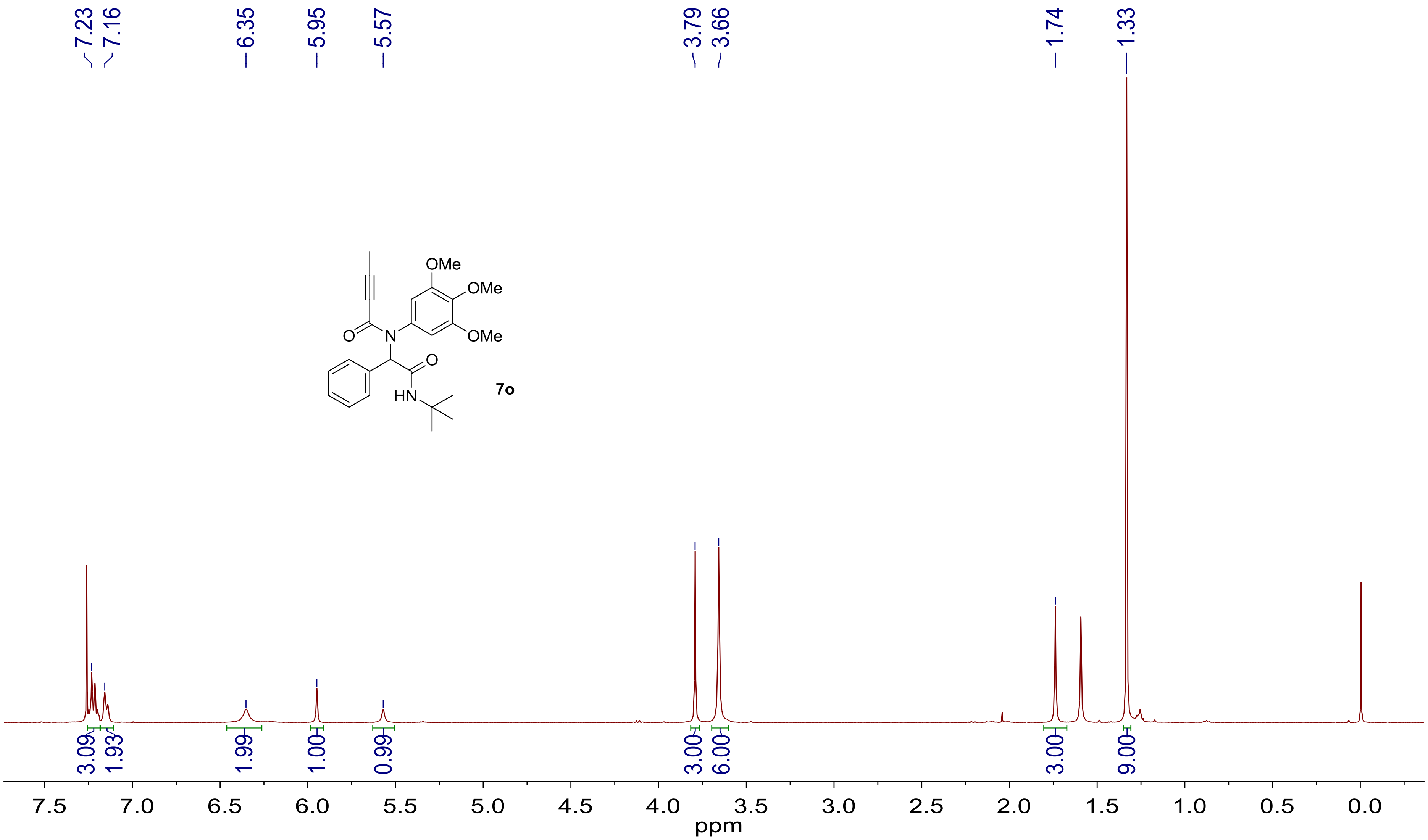
7.23
7.16
6.35
5.95
5.57
3.79
3.66
1.74
1.33
OMe
OMe
OMe
O
N
O
HN
3.09
1.93
1.99
1.00
0.99
3.00
6.00
3.00
9.00
7.5
7.0
6.5
6.0
5.5
5.0
4.5
4.0
3.5
3.0
2.5
2.0
1.5
1.0
0.5
0.0
ppm

7o

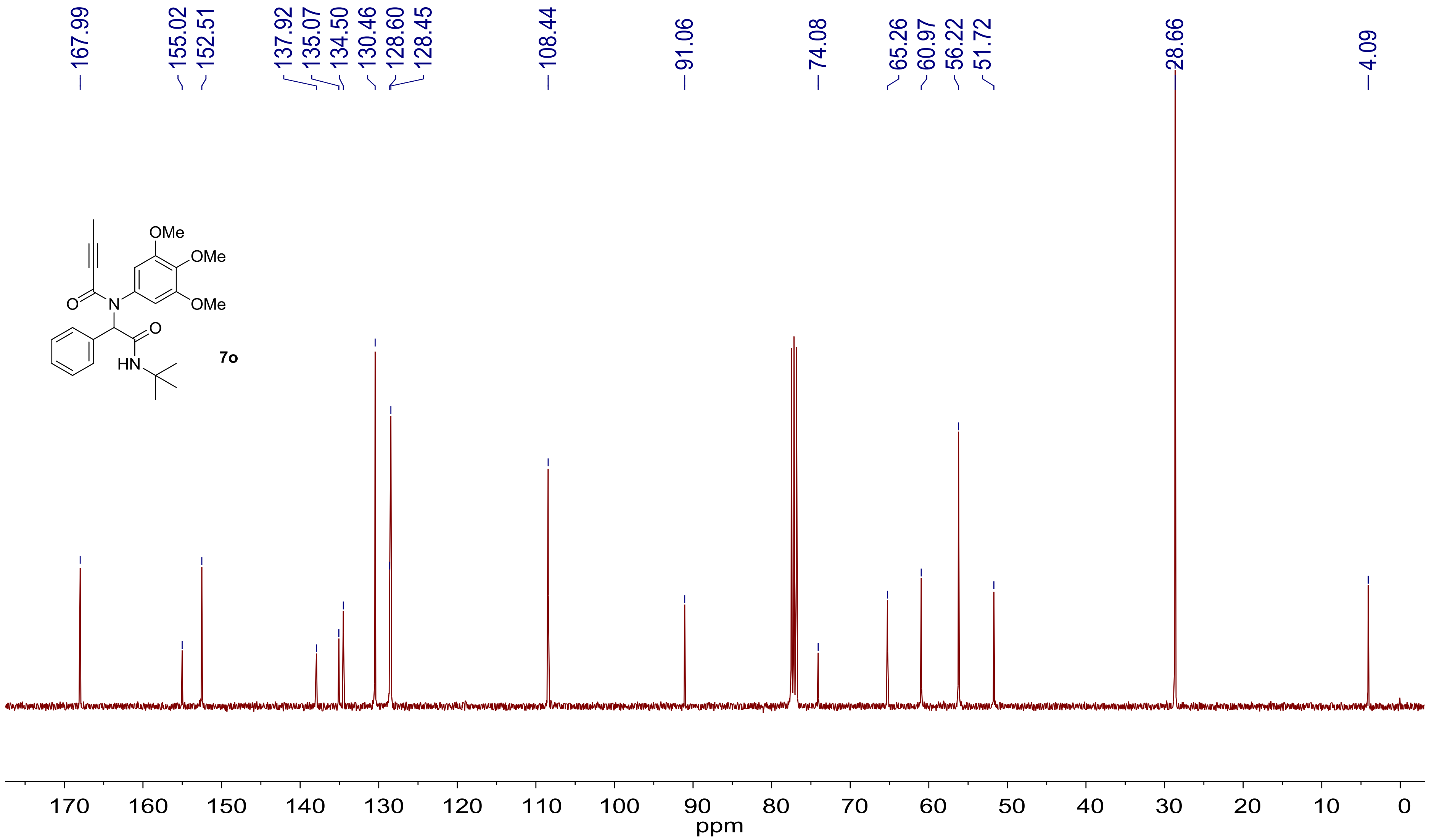
167.99
155.02
152.51
137.92
135.07
134.50
130.46
128.60
128.45
108.44
91.06
74.08
65.26
60.97
56.22
51.72
28.66
4.09
OMe
OMe
OMe
O
N
O
HN
7o
170 160 150 140 130 120 110 100 90 80 70 60 50 40 30 20 10 0
ppm

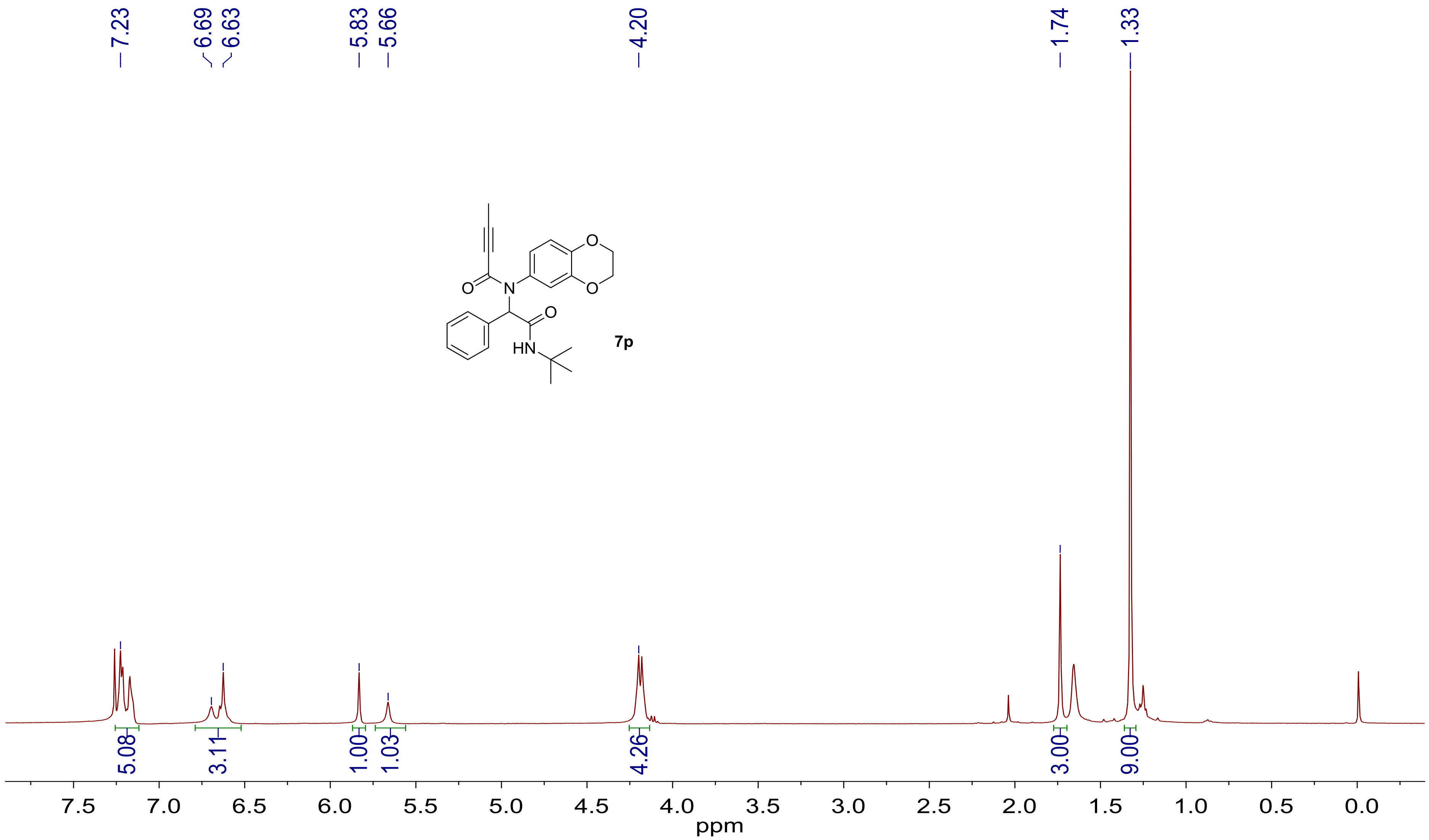
7.23
6.69
6.63
5.83
5.66
4.20
1.74
1.33
O
O
O
N
O
O
HN
7p
5.08
3.11
1.00
1.03
4.26
3.00
9.00
7.5
7.0
6.5
6.0
5.5
5.0
4.5
4.0
3.5
3.0
2.5
2.0
1.5
1.0
0.5
0.0
ppm

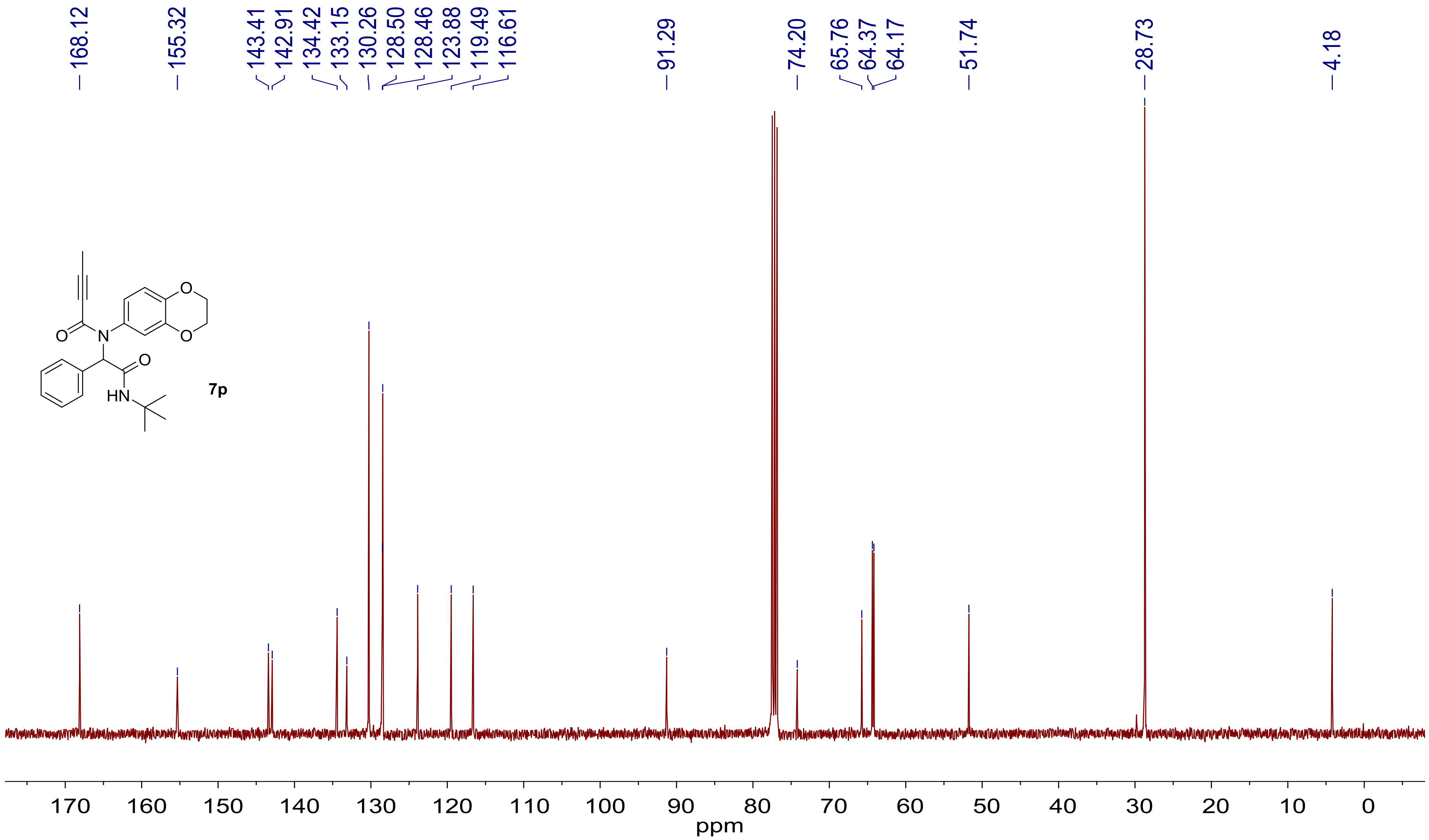
168.12
155.32
143.41
142.91
134.42
133.15
130.26
128.50
128.46
123.88
119.49
116.61
91.29
74.20
65.76
64.37
64.17
51.74
28.73
4.18
O
N
O
O
O
HN
7p
170 160 150 140 130 120 110 100 90 80 70 60 50 40 30 20 10 0
ppm

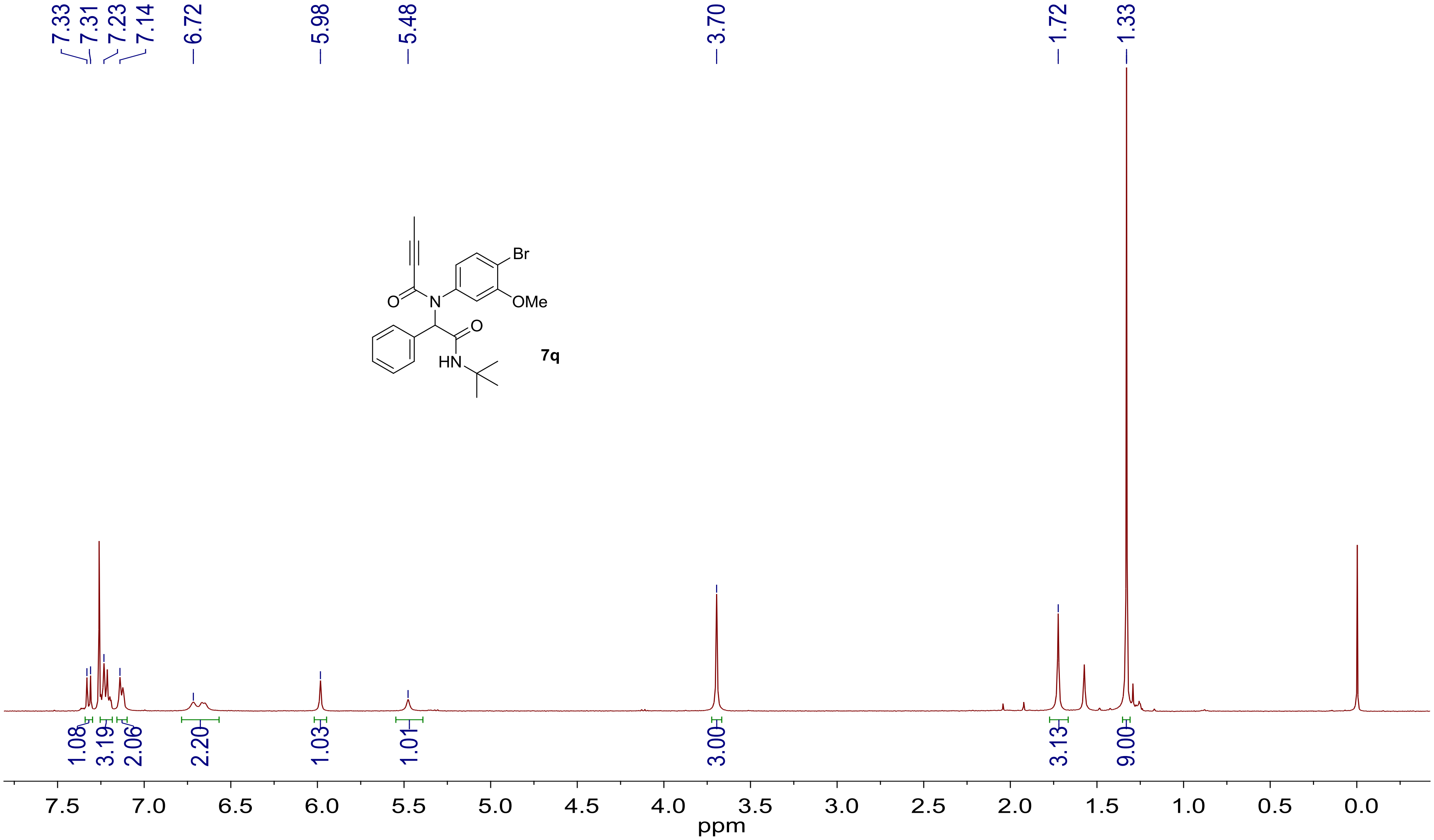
7.33
7.31
7.23
7.14
6.72
5.98
5.48
3.70
1.72
1.33
Br
O
N
OMe
O
HN
7q
1.08
3.19
2.06
2.20
1.03
1.01
3.00
3.13
9.00
7.5
7.0
6.5
6.0
5.5
5.0
4.5
4.0
3.5
3.0
2.5
2.0
1.5
1.0
0.5
0.0
ppm

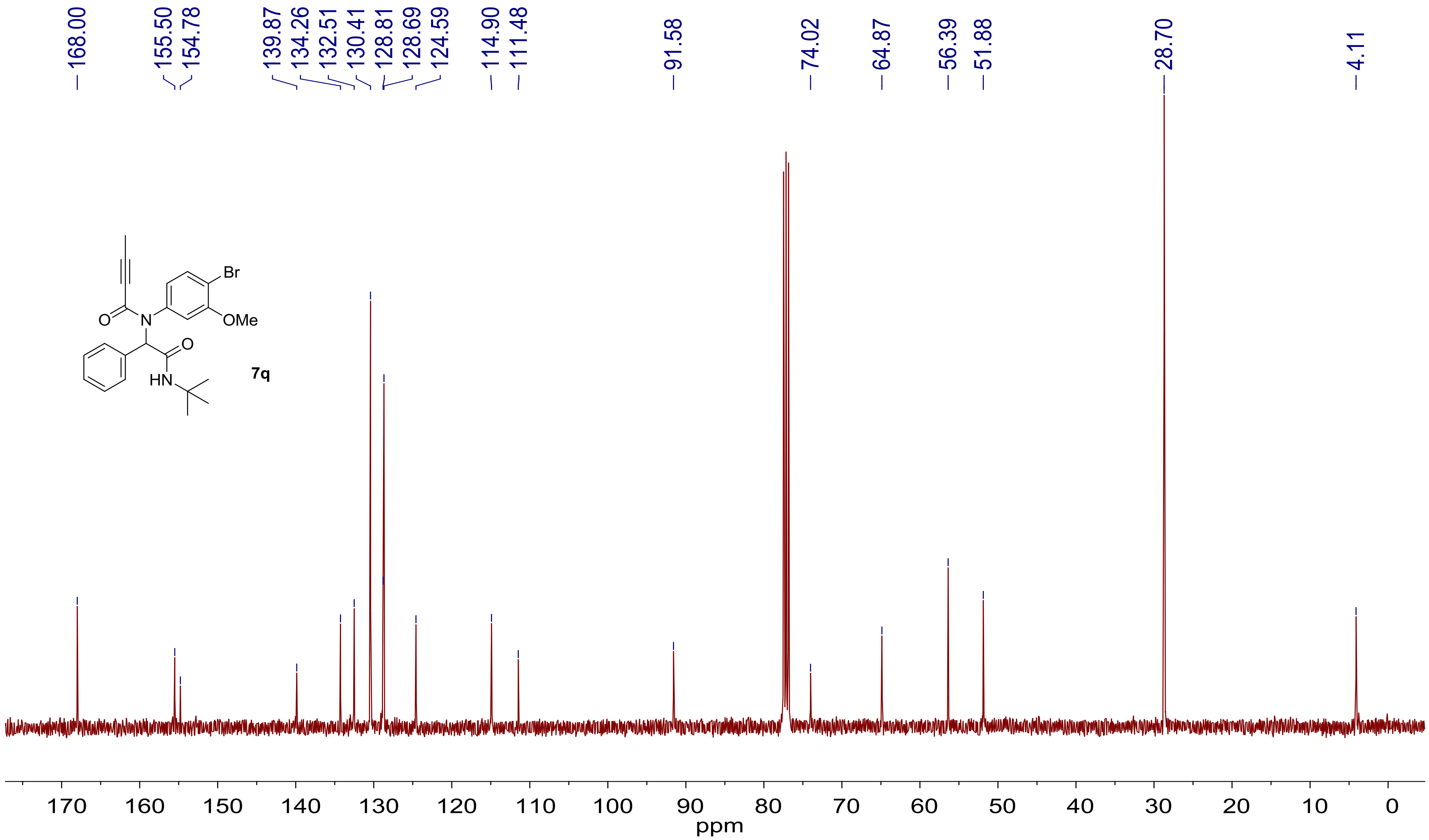
168.00
155.50
154.78
139.87
134.26
132.51
130.41
128.81
128.69
124.59
114.90
111.48
91.58
74.02
64.87
56.39
51.88
28.70
4.11
Br
O
N
OMe
O
HN
7q
170 160 150 140 130 120 110 100 90 80 70 60 50 40 30 20 10 0
ppm

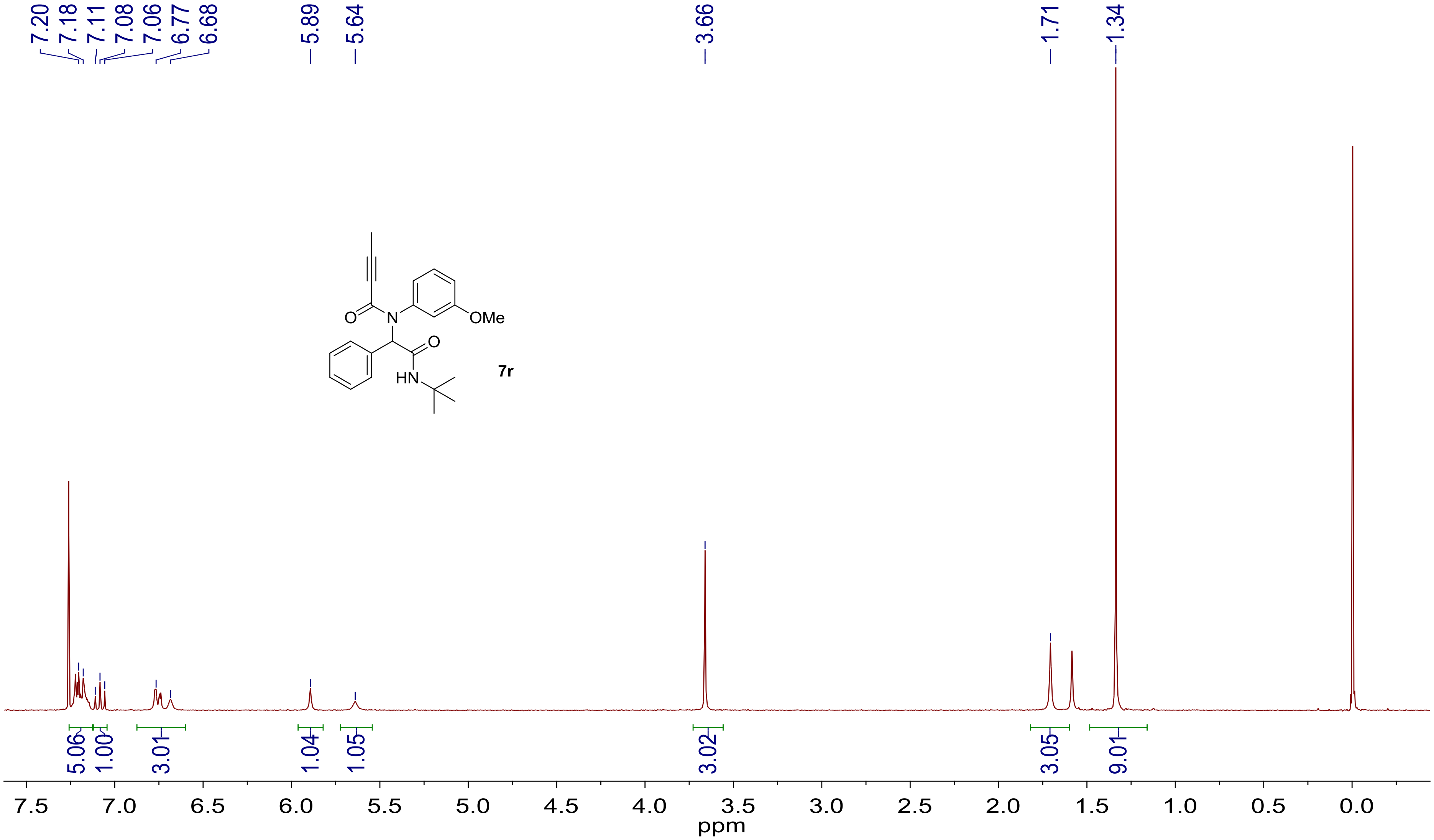

7.20
7.18
7.11
7.08
7.06
6.77
6.68
5.89
5.64
3.66
1.71
1.34
O
N
OMe
O
HN
7r
5.06
1.00
3.01
1.04
1.05
3.02
3.05
9.01
7.5
7.0
6.5
6.0
5.5
5.0
4.5
4.0
3.5
3.0
2.5
2.0
1.5
1.0
0.5
0.0
ppm

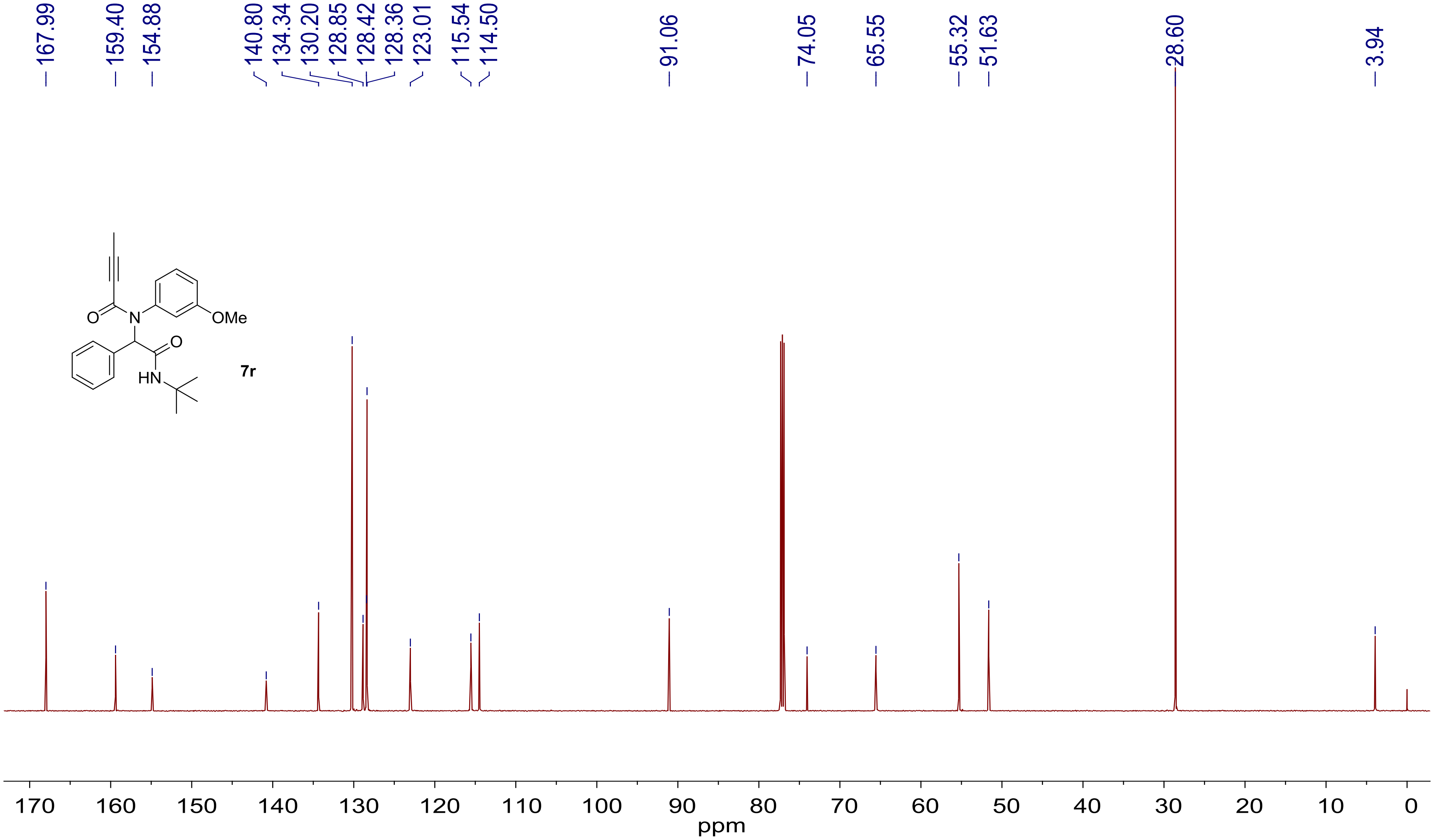
167.99
159.40
154.88
140.80
134.34
130.20
128.85
128.42
128.36
123.01
115.54
114.50
91.06
74.05
65.55
55.32
51.63
28.60
3.94
O
N
OMe
O
HN
7r
170 160 150 140 130 120 110 100 90 80 70 60 50 40 30 20 10 0
ppm

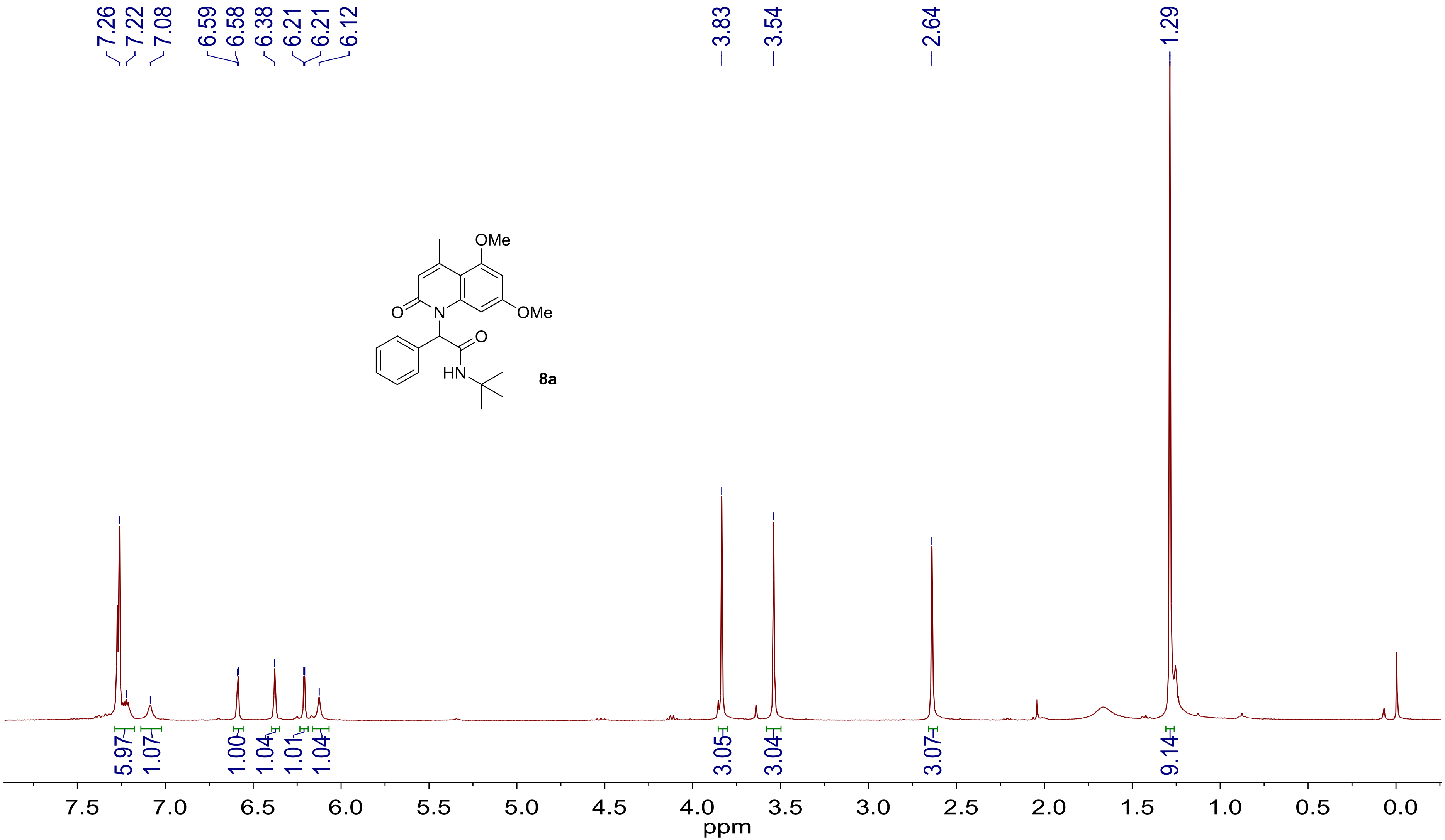
7.26
7.22
7.08
6.59
6.58
6.38
6.21
6.21
6.12
3.83
3.54
2.64
1.29
OMe
O
N
OMe
O
HN
8a
5.97
1.07
1.00
1.04
1.01
1.04
3.05
3.04
3.07
9.14
7.5
7.0
6.5
6.0
5.5
5.0
4.5
4.0
3.5
3.0
2.5
2.0
1.5
1.0
0.5
0.0
ppm

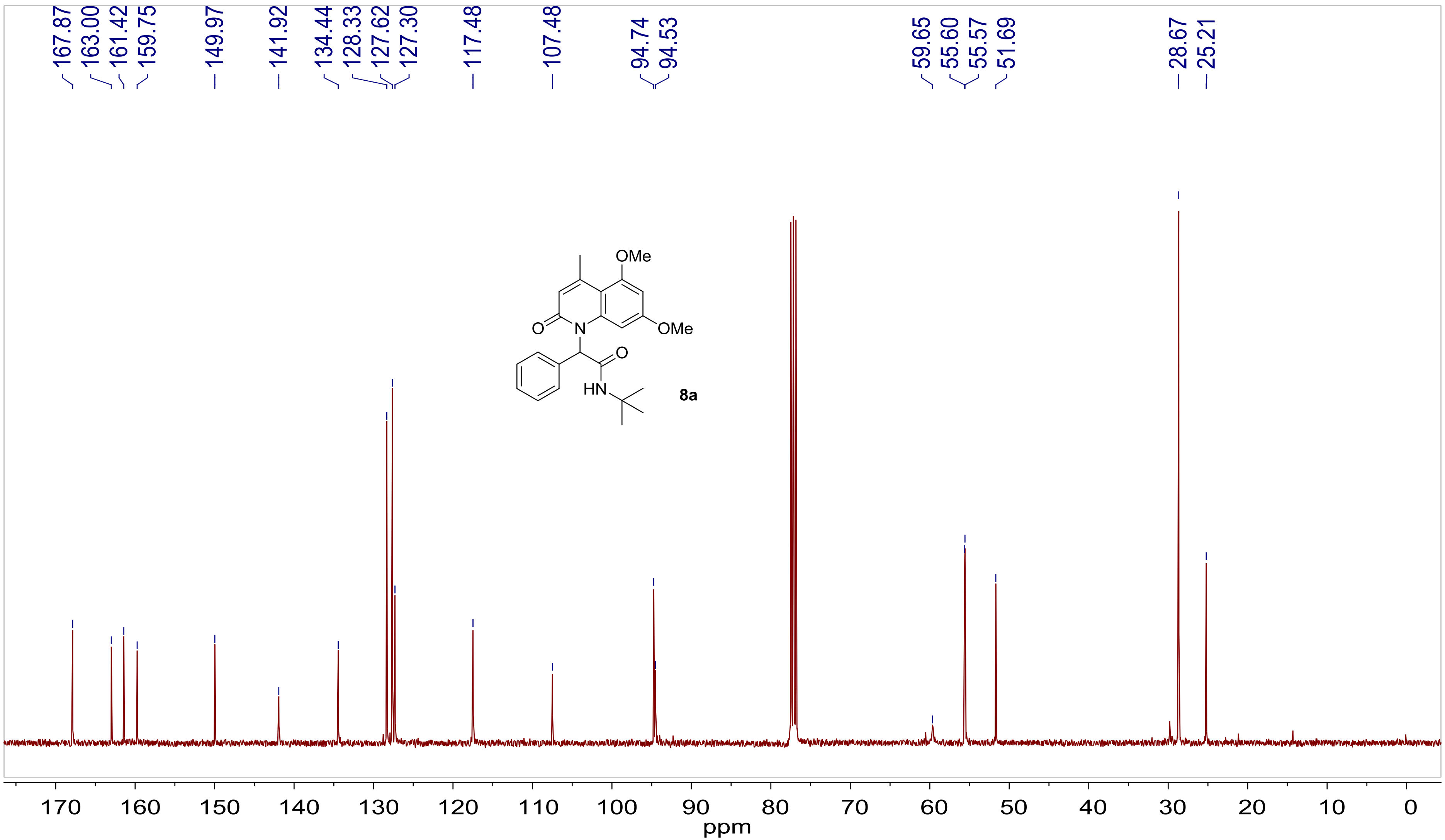
167.87
163.00
161.42
159.75
149.97
141.92
134.44
128.33
127.62
127.30
117.48
107.48
94.74
94.53
59.65
55.60
55.57
51.69
28.67
25.21
OMe
O
N
OMe
O
HN
8a
170 160 150 140 130 120 110 100 90 80 70 60 50 40 30 20 10 0
ppm

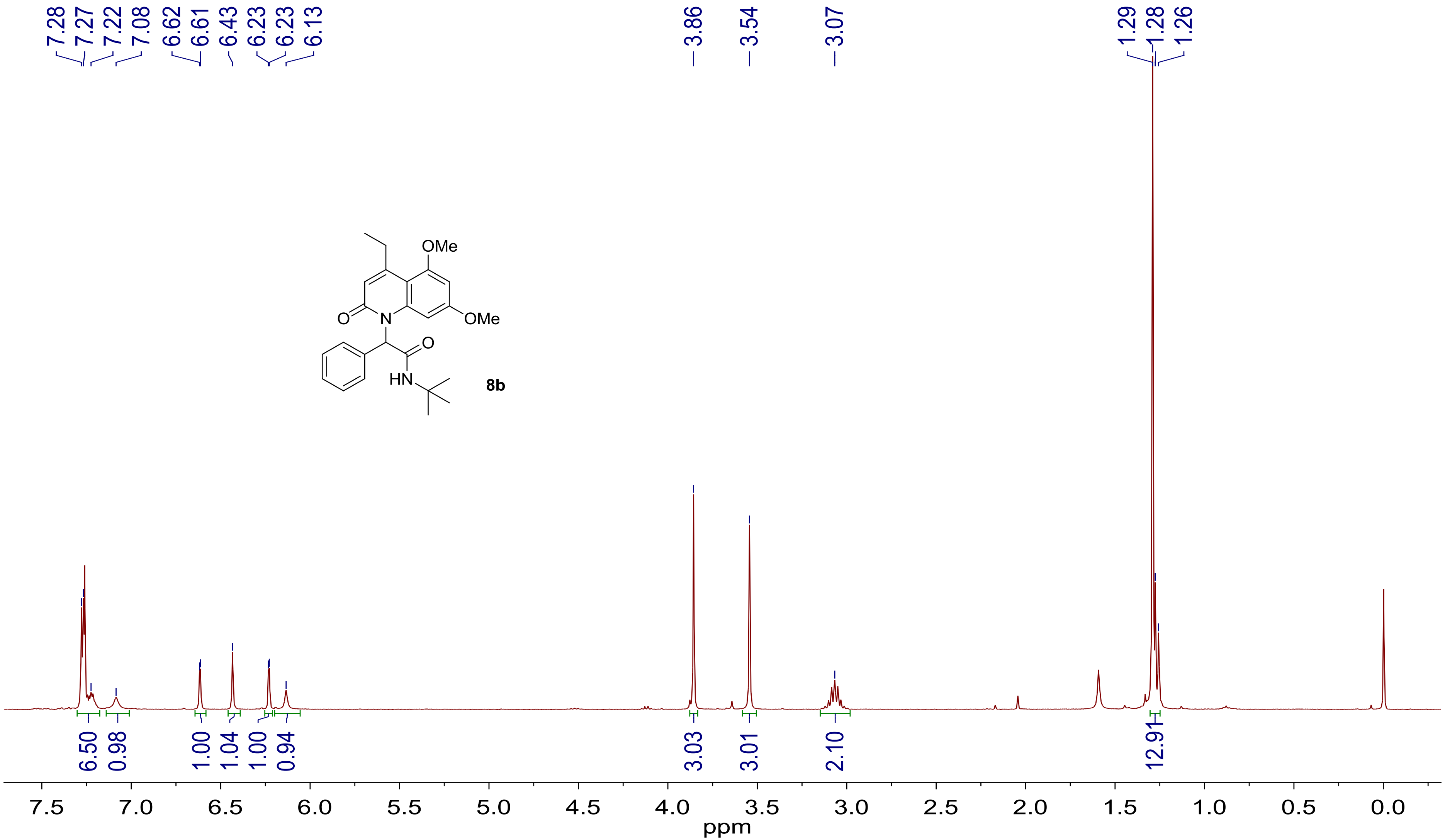
7.28 7.27 7.22 7.08 6.62 6.61 6.43 6.23 6.23 6.13
3.86
3.54
3.07
1.29 1.28 1.26
OMe
OMe
O
N
O
HN
8b
6.50 0.98 1.00 1.04 1.00 0.94
3.03 3.01 2.10
12.91
7.5 7.0 6.5 6.0 5.5 5.0 4.5 4.0 3.5 3.0 2.5 2.0 1.5 1.0 0.5 0.0
ppm

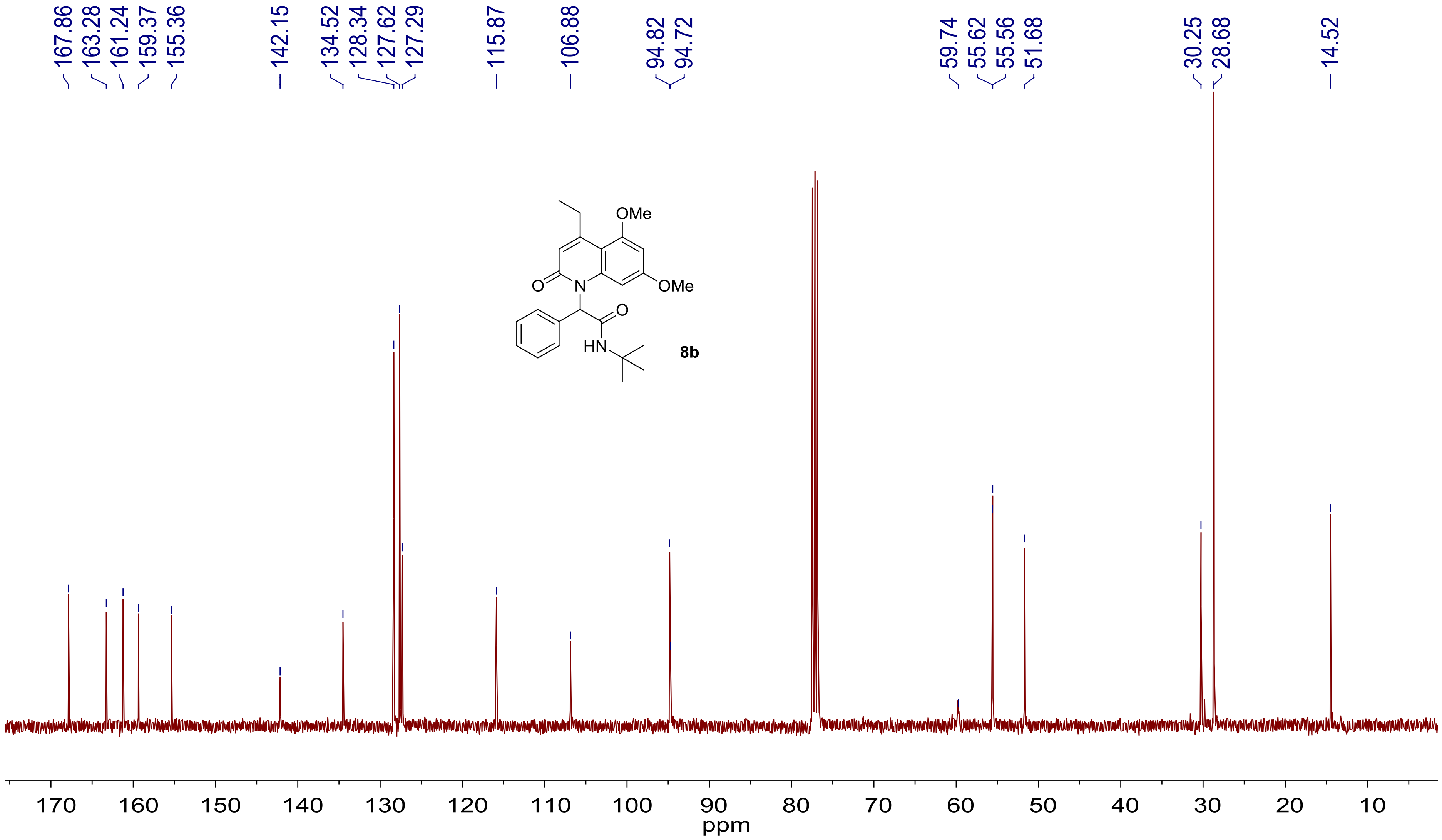
167.86
163.28
161.24
159.37
155.36
142.15
134.52
128.34
127.62
127.29
115.87
106.88
94.82
94.72
59.74
55.62
55.56
51.68
30.25
28.68
14.52
OMe
OMe
O
N
O
HN
8b
170
160
150
140
130
120
110
100
90
80
70
60
50
40
30
20
10
ppm

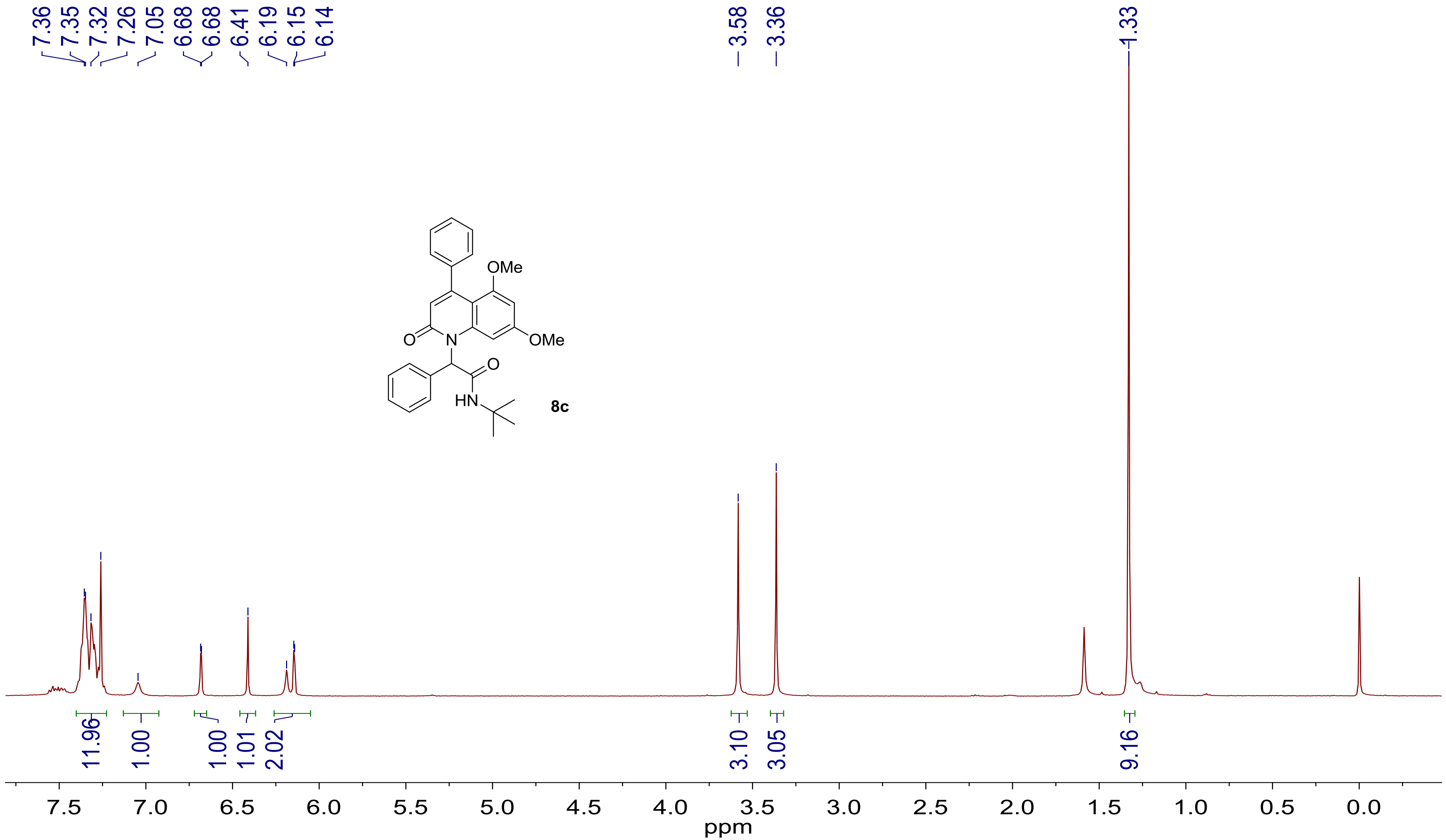
7.36
7.35
7.32
7.26
7.05
6.68
6.68
6.41
6.19
6.15
6.14
3.58
3.36
1.33
OMe
O
N
OMe
O
HN
8c
11.96
1.00
1.00
1.01
2.02
3.10
3.05
9.16
7.5
7.0
6.5
6.0
5.5
5.0
4.5
4.0
3.5
3.0
2.5
2.0
1.5
1.0
0.5
0.0
ppm

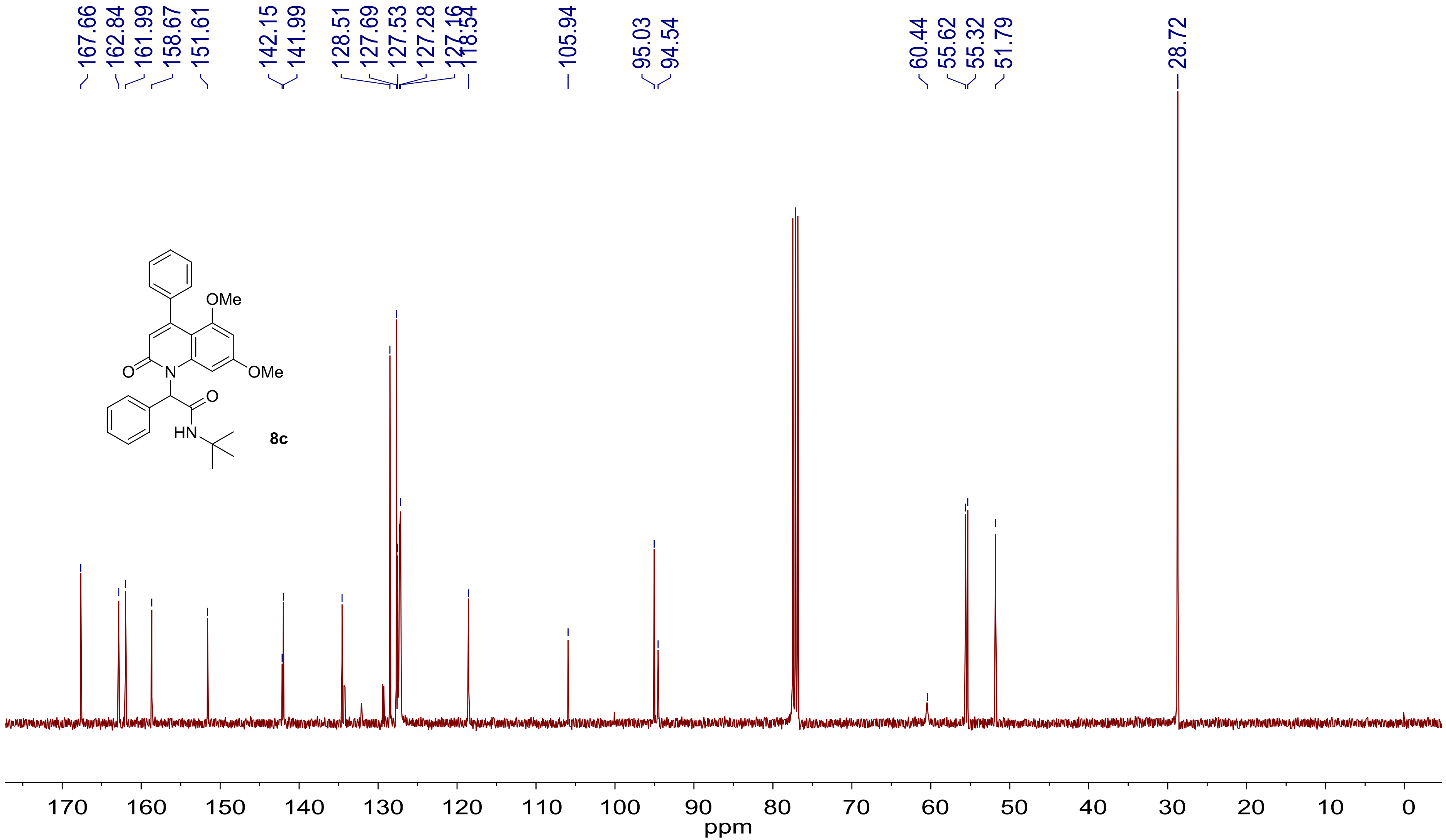
167.66
162.84
161.99
158.67
151.61
142.15
141.99
128.51
127.69
127.53
127.28
127.16
118.54
105.94
95.03
94.54
60.44
55.62
55.32
51.79
28.72
OMe
OMe
O
N
O
HN
8c
170 160 150 140 130 120 110 100 90 80 70 60 50 40 30 20 10 0
ppm

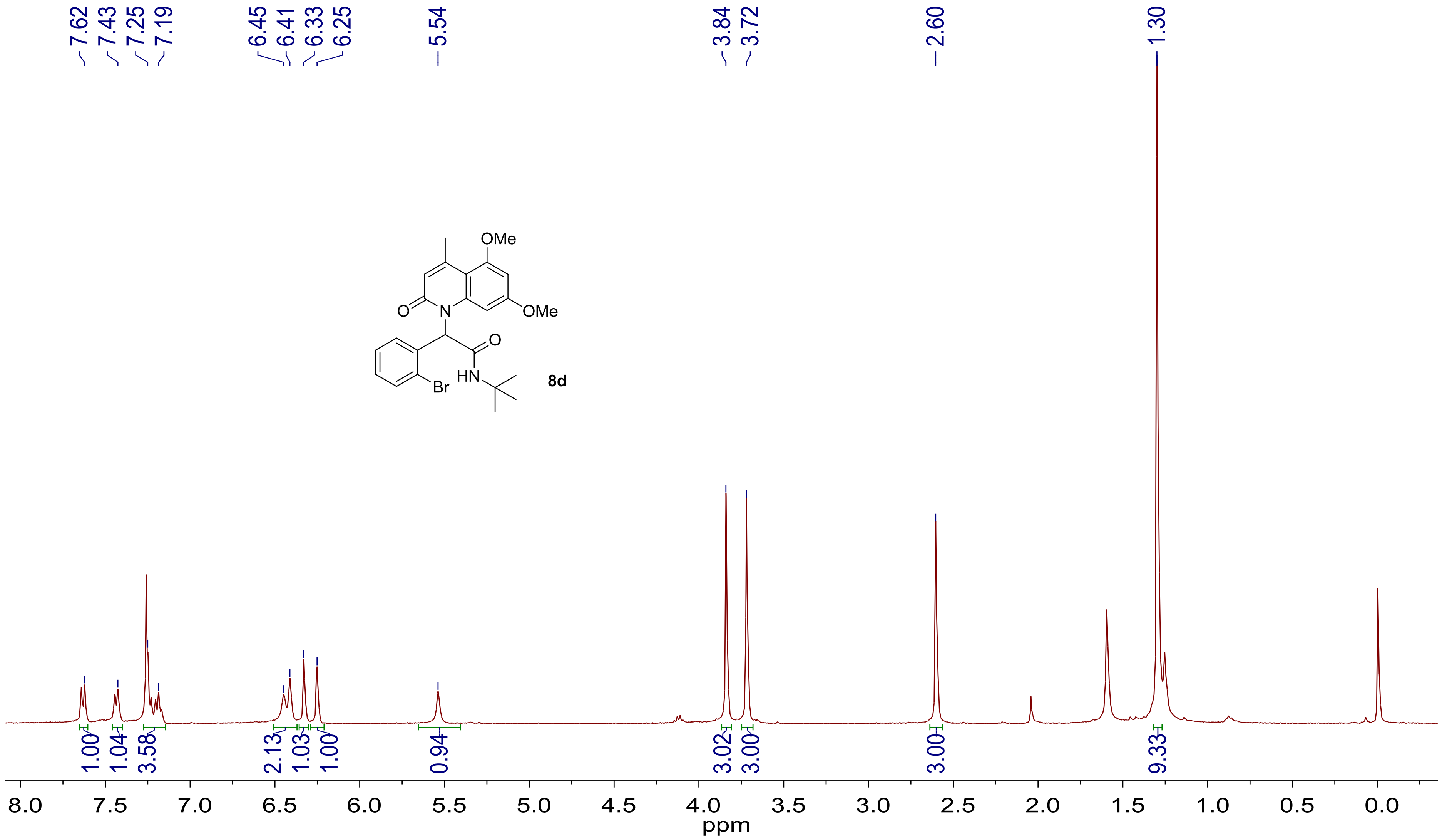
7.62
7.43
7.25
7.19
6.45
6.41
6.33
6.25
5.54
3.84
3.72
2.60
1.30
OMe
O
N
OMe
O
HN
Br
8d
1.00
1.04
3.58
2.13
1.03
1.00
0.94
3.02
3.00
3.00
9.33
8.0
7.5
7.0
6.5
6.0
5.5
5.0
4.5
4.0
3.5
3.0
2.5
2.0
1.5
1.0
0.5
0.0
ppm

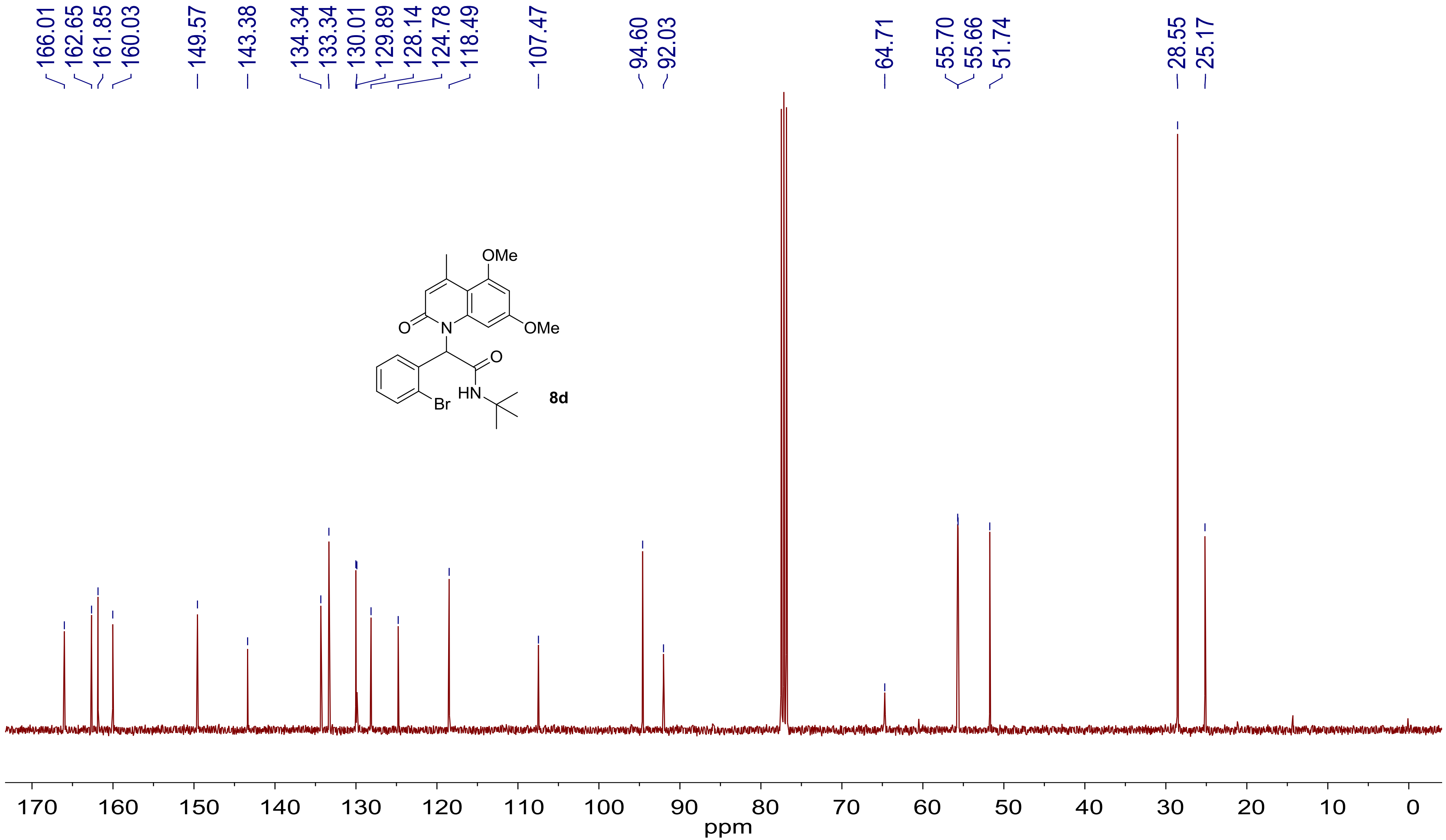
166.01
162.65
161.85
160.03
149.57
143.38
134.34
133.34
130.01
129.89
128.14
124.78
118.49
107.47
94.60
92.03
64.71
55.70
55.66
51.74
28.55
25.17
OMe
OMe
O
N
O
HN
Br
8d
170 160 150 140 130 120 110 100 90 80 70 60 50 40 30 20 10 0
ppm

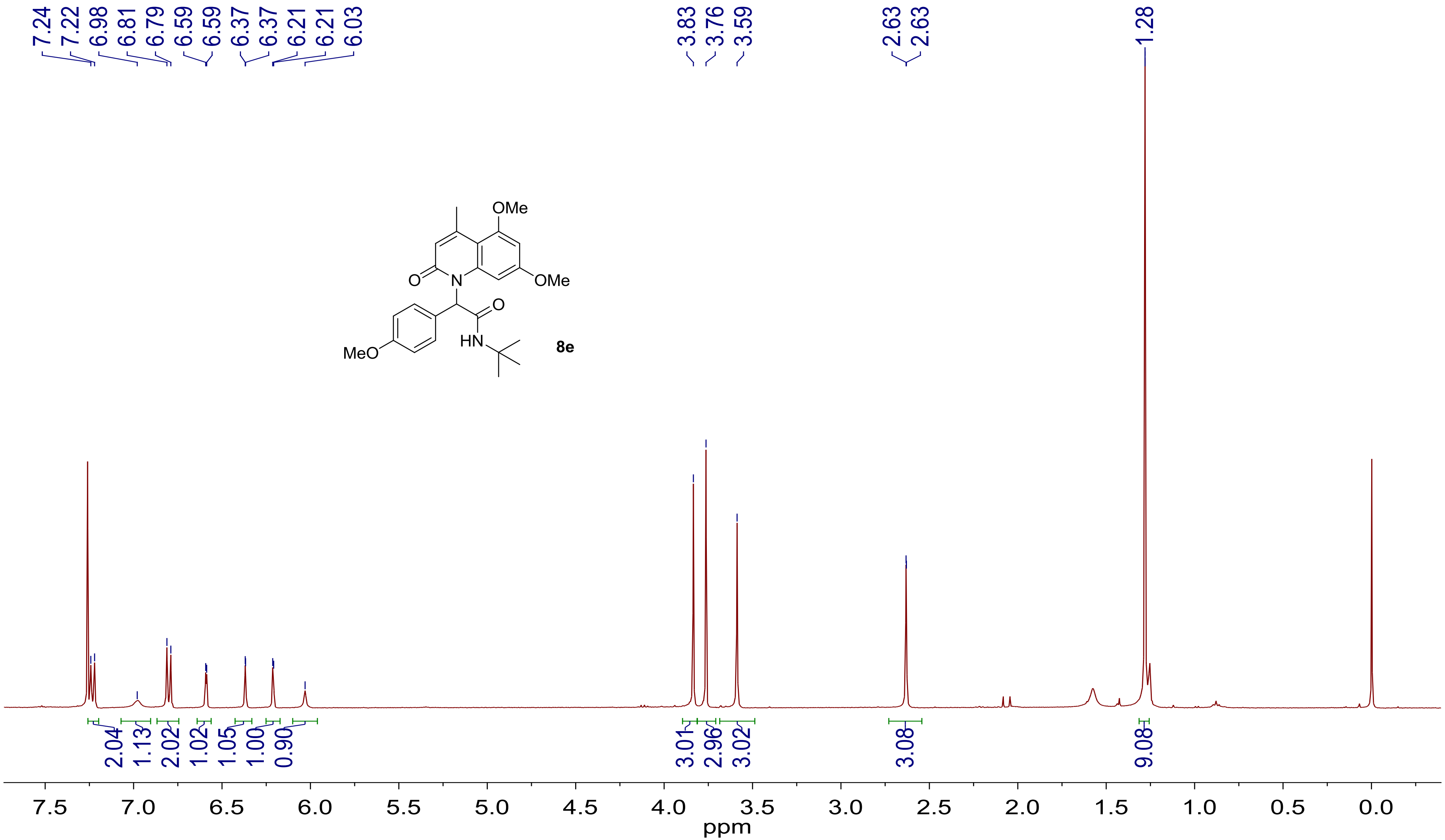
7.24
7.22
6.98
6.81
6.79
6.59
6.59
6.37
6.37
6.21
6.21
6.03
3.83
3.76
3.59
2.63
2.63
1.28
OMe
O
N
OMe
O
HN
MeO
8e
2.04
1.13
2.02
1.02
1.05
1.00
0.90
3.01
2.96
3.02
3.08
9.08
7.5
7.0
6.5
6.0
5.5
5.0
4.5
4.0
3.5
3.0
2.5
2.0
1.5
1.0
0.5
0.0
ppm

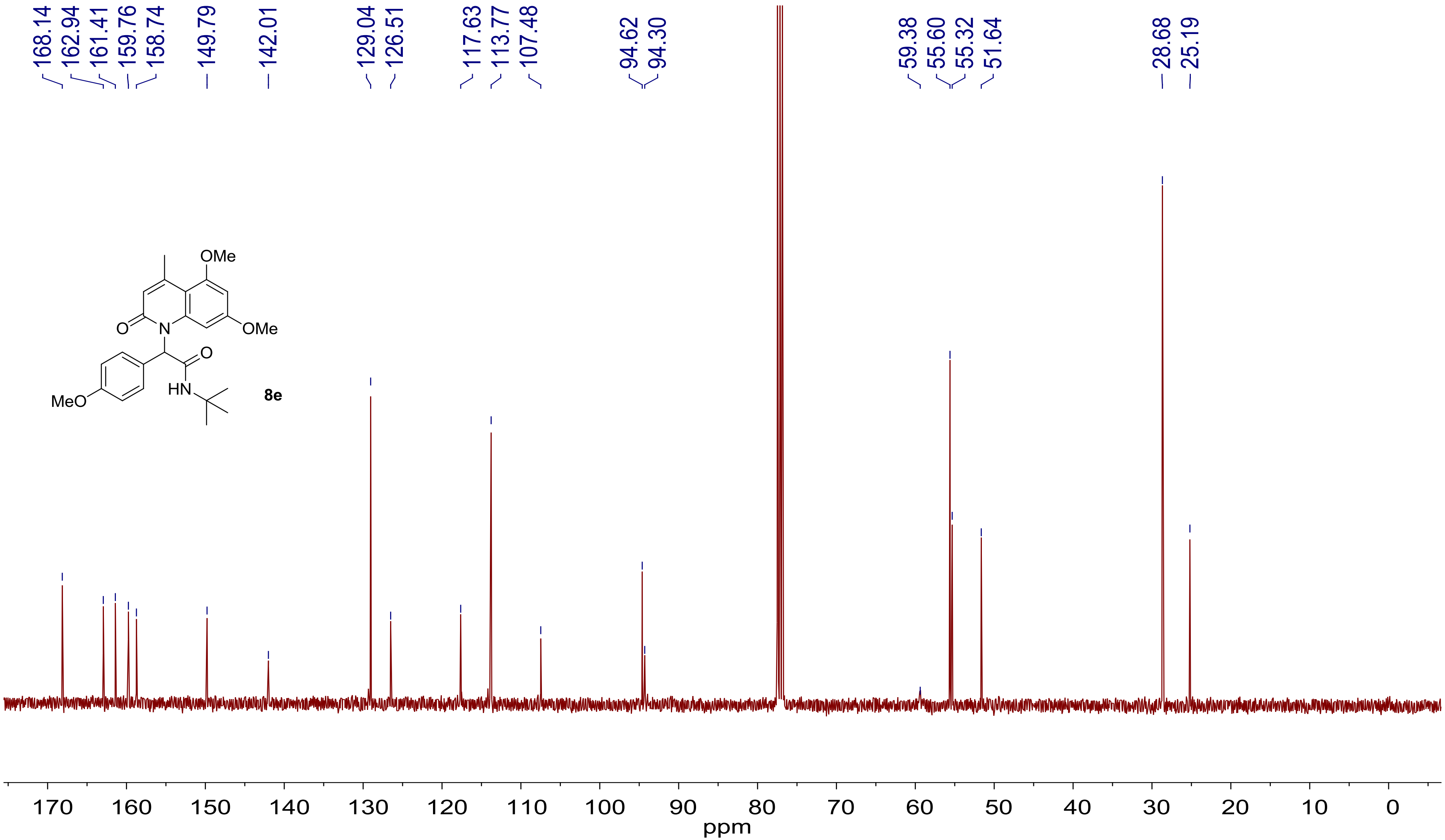
168.14
162.94
161.41
159.76
158.74
149.79
142.01
129.04
126.51
117.63
113.77
107.48
94.62
94.30
59.38
55.60
55.32
51.64
28.68
25.19
OMe
OMe
O
N
O
HN
MeO
8e
170 160 150 140 130 120 110 100 90 80 70 60 50 40 30 20 10 0
ppm

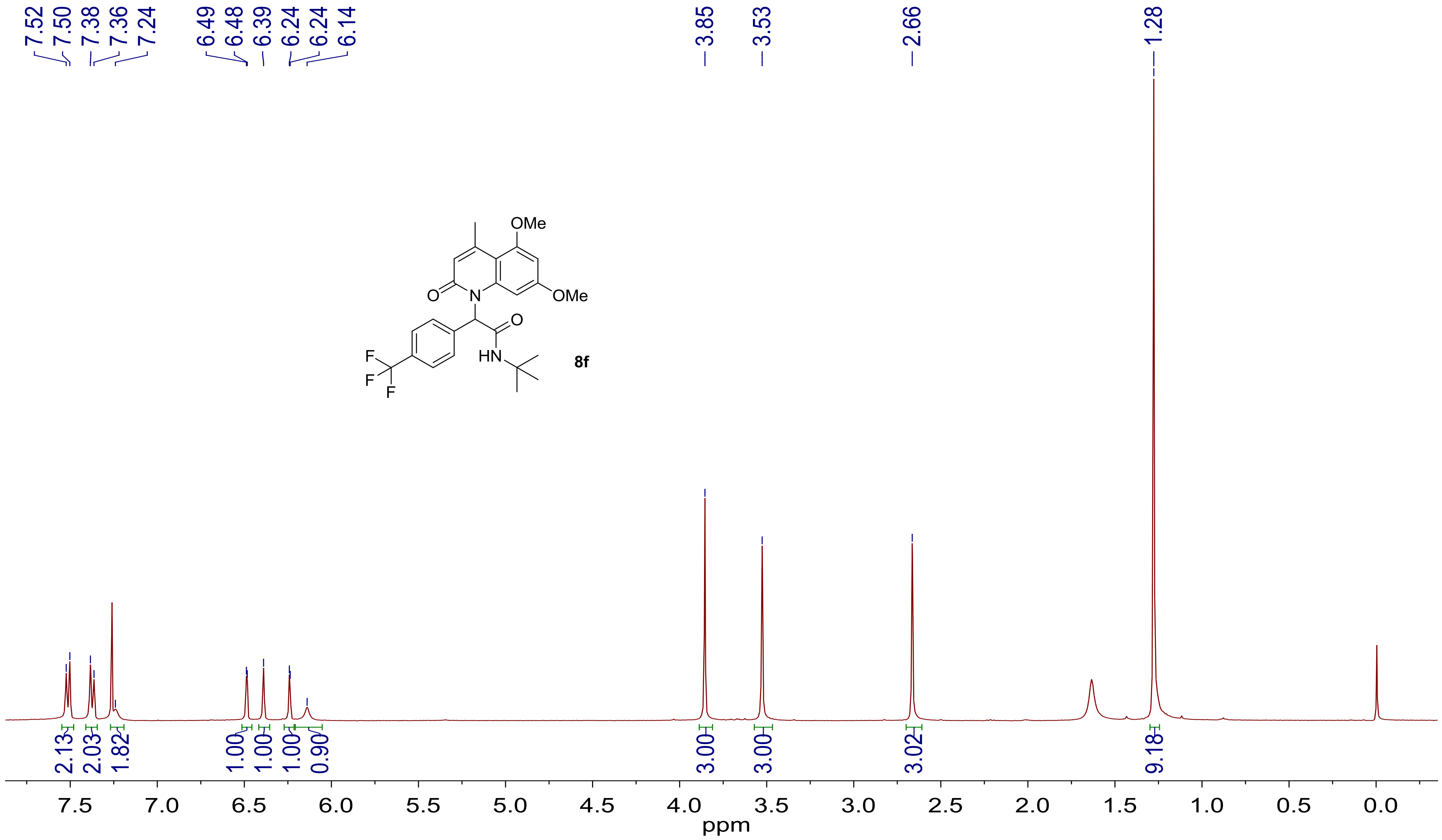

7.52
7.50
7.38
7.36
7.24
6.49
6.48
6.39
6.24
6.24
6.14
3.85
3.53
2.66
1.28
OMe
O
N
OMe
O
HN
F
F
F
8f
2.13
2.03
1.82
1.00
1.00
1.00
0.90
3.00
3.00
3.02
9.18
7.5
7.0
6.5
6.0
5.5
5.0
4.5
4.0
3.5
3.0
2.5
2.0
1.5
1.0
0.5
0.0
ppm

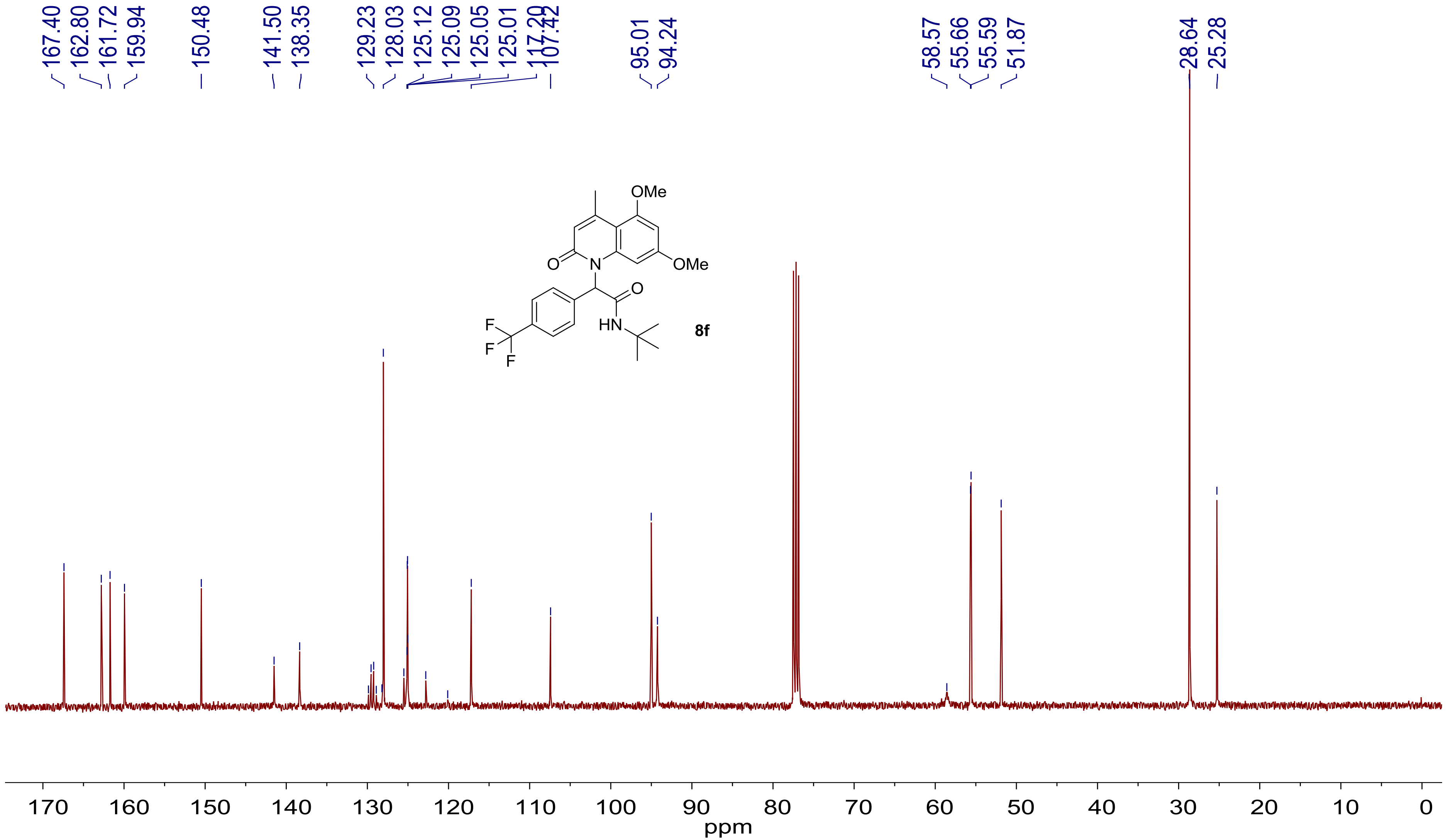
167.40
162.80
161.72
159.94
150.48
141.50
138.35
129.23
128.03
125.12
125.09
125.05
125.01
117.20
107.42
95.01
94.24
58.57
55.66
55.59
51.87
28.64
25.28
OMe
OMe
O
N
O
HN
F
F
F
8f
170 160 150 140 130 120 110 100 90 80 70 60 50 40 30 20 10 0
ppm

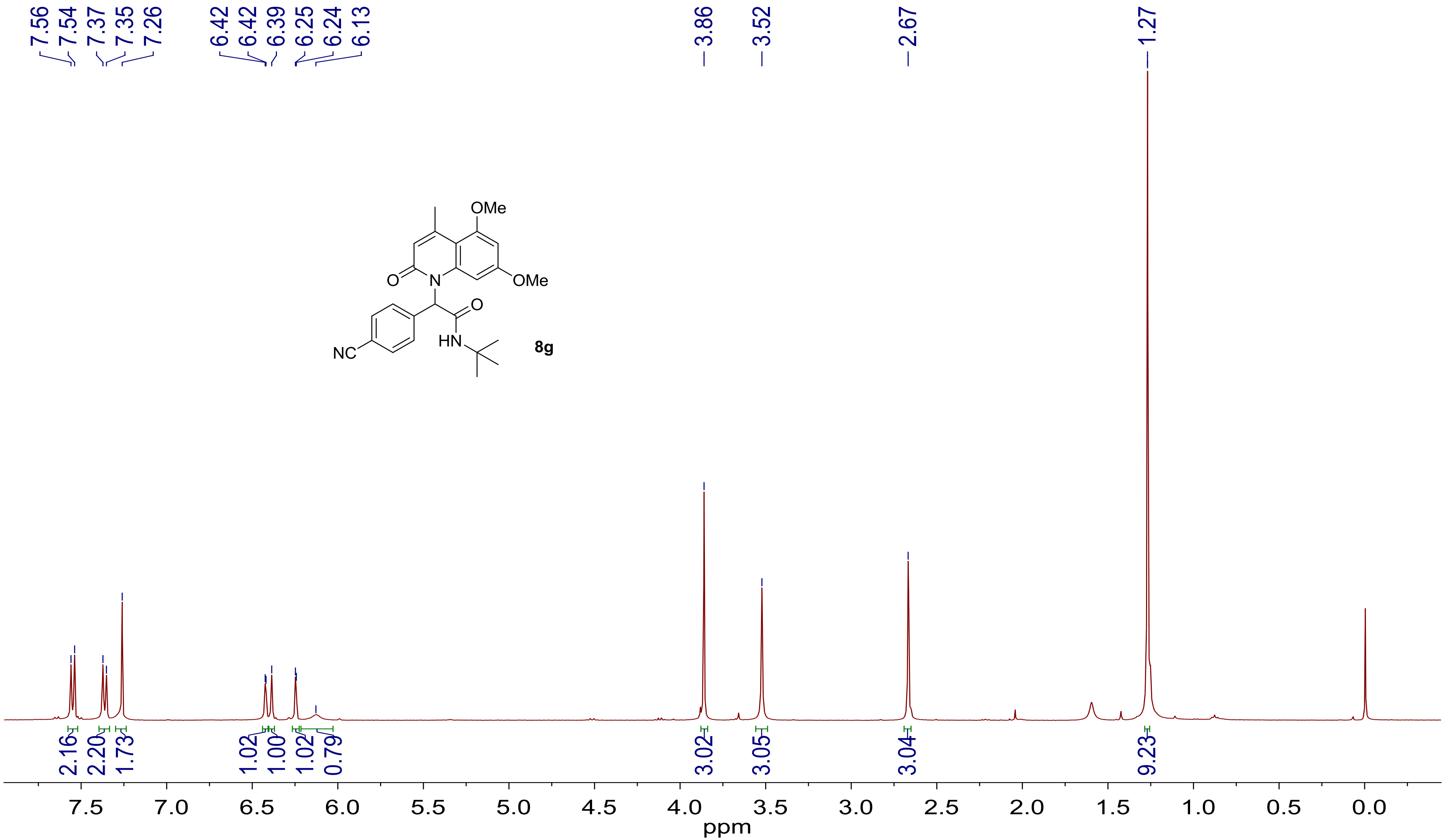
7.56
7.54
7.37
7.35
7.26
6.42
6.42
6.39
6.25
6.24
6.13
3.86
3.52
2.67
1.27
OMe
O
N
OMe
O
NC
HN
8g
2.16
2.20
1.73
1.02
1.00
1.02
0.79
3.02
3.05
3.04
9.23
7.5
7.0
6.5
6.0
5.5
5.0
4.5
4.0
3.5
3.0
2.5
2.0
1.5
1.0
0.5
0.0
ppm

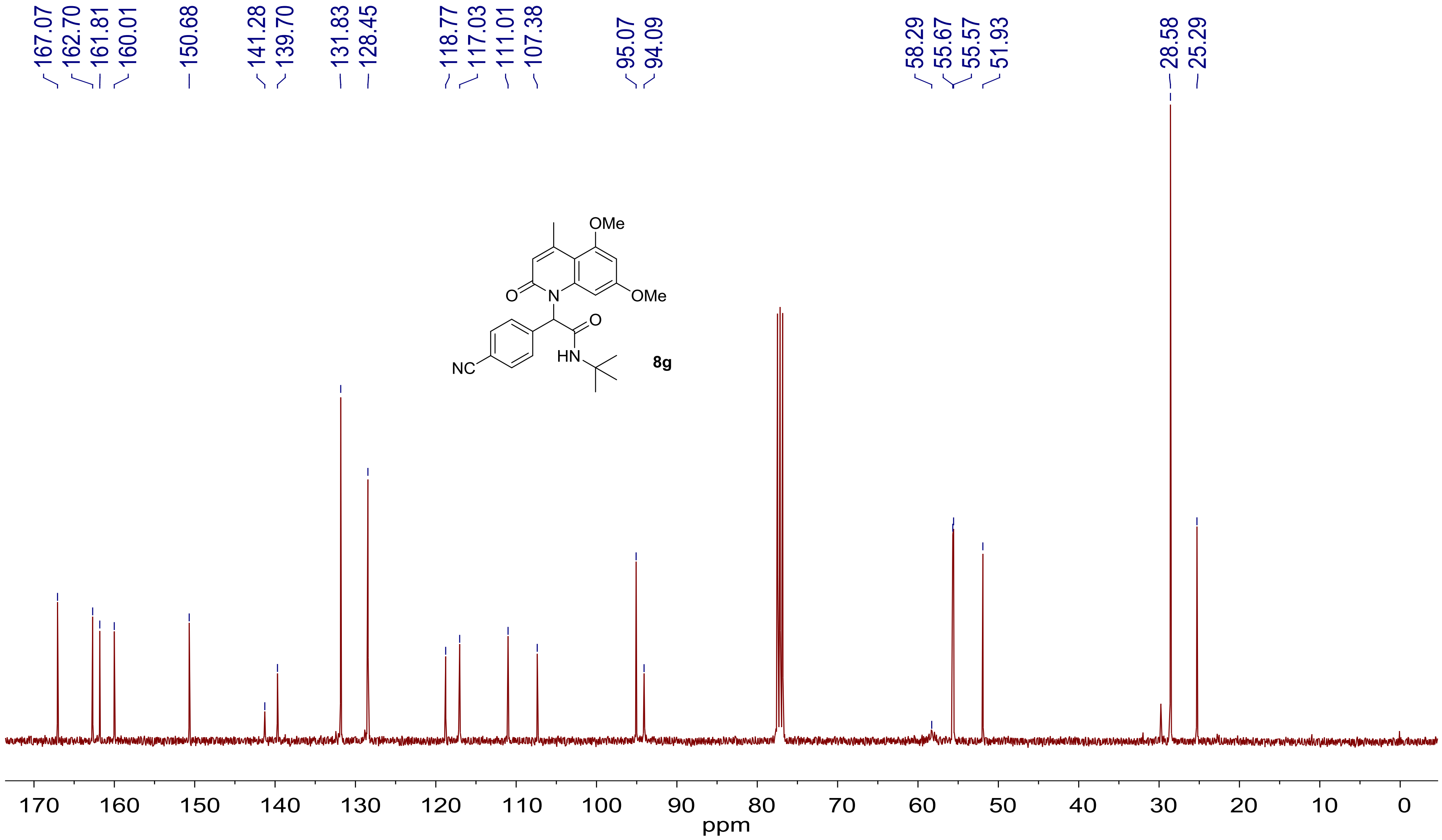

167.07
162.70
161.81
160.01
150.68
141.28
139.70
131.83
128.45
118.77
117.03
111.01
107.38
95.07
94.09
58.29
55.67
55.57
51.93
28.58
25.29
OMe
OMe
O
N
O
HN
NC
8g
170
160
150
140
130
120
110
100
90
80
70
60
50
40
30
20
10
0
ppm

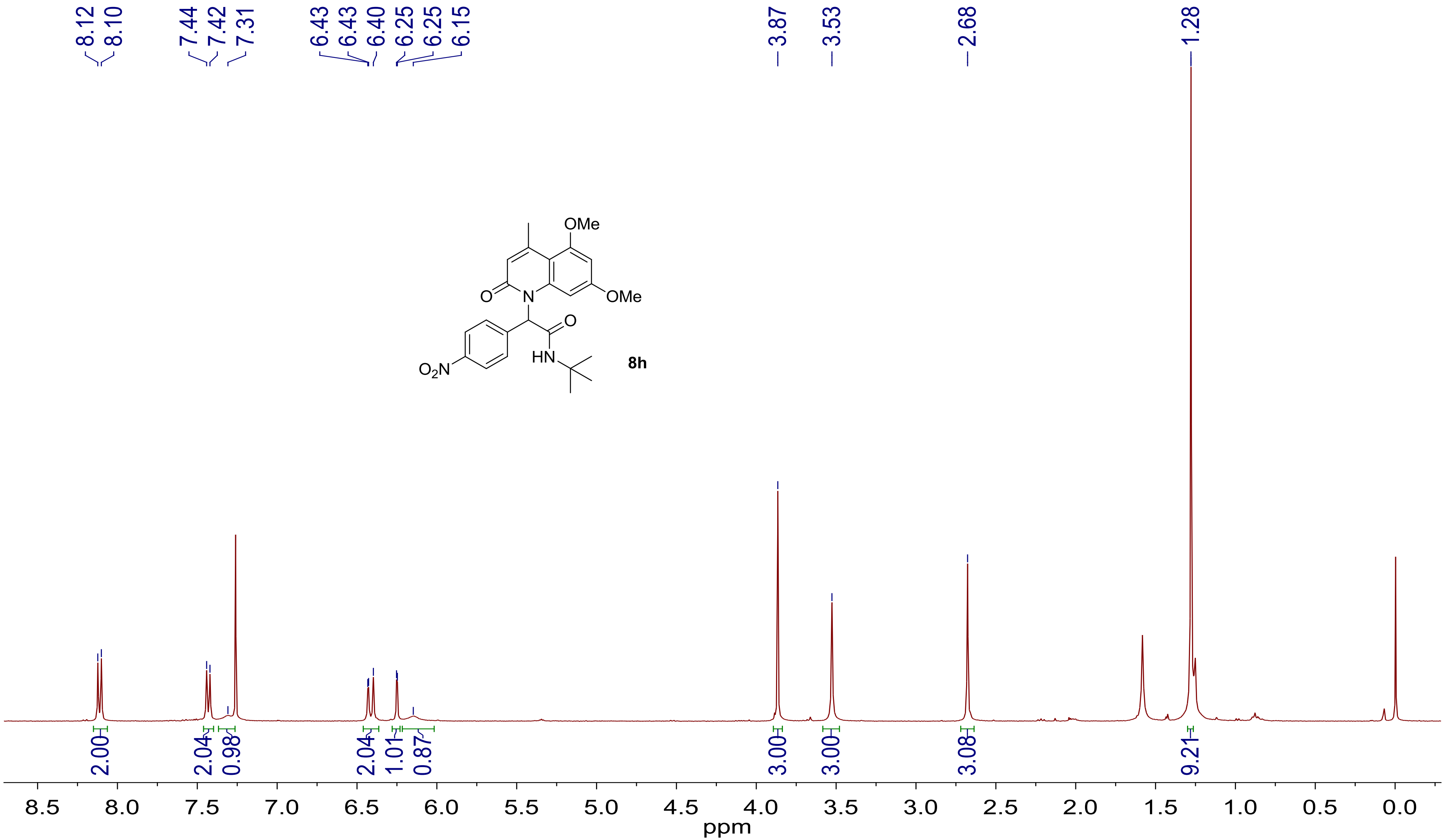
8.12
8.10
7.44
7.42
7.31
6.43
6.43
6.40
6.25
6.25
6.15
3.87
3.53
2.68
1.28
OMe
O
N
OMe
O
HN
O2N
2.00
2.04
0.98
2.04
1.01
0.87
3.00
3.00
3.08
9.21
8.5
8.0
7.5
7.0
6.5
6.0
5.5
5.0
4.5
4.0
3.5
3.0
2.5
2.0
1.5
1.0
0.5
0.0
ppm

8h

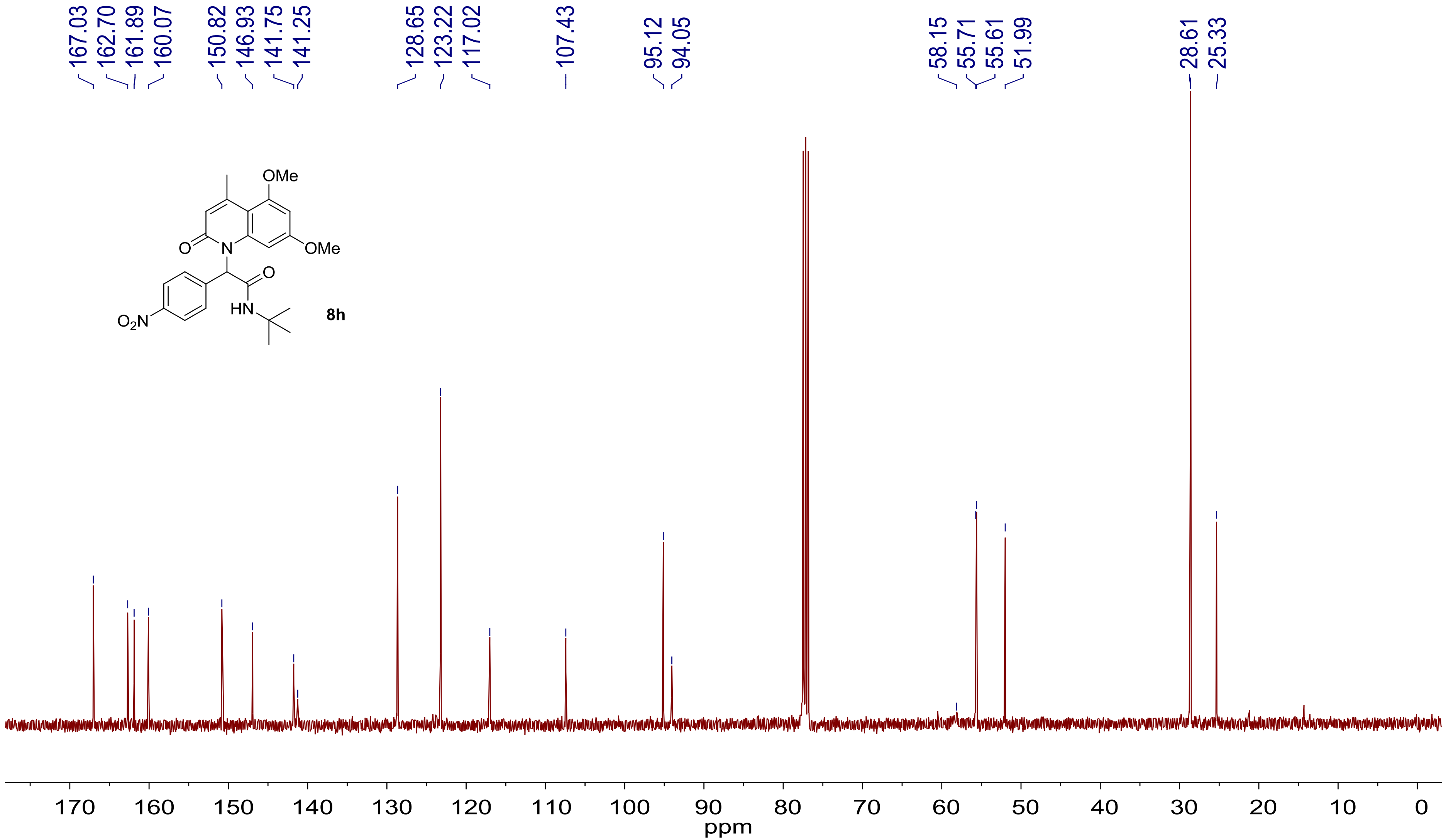
167.03
162.70
161.89
160.07
150.82
146.93
141.75
141.25
128.65
123.22
117.02
107.43
95.12
94.05
58.15
55.71
55.61
51.99
28.61
25.33
OMe
O
N
OMe
O
O_2N
HN
8h
170 160 150 140 130 120 110 100 90 80 70 60 50 40 30 20 10 0
ppm

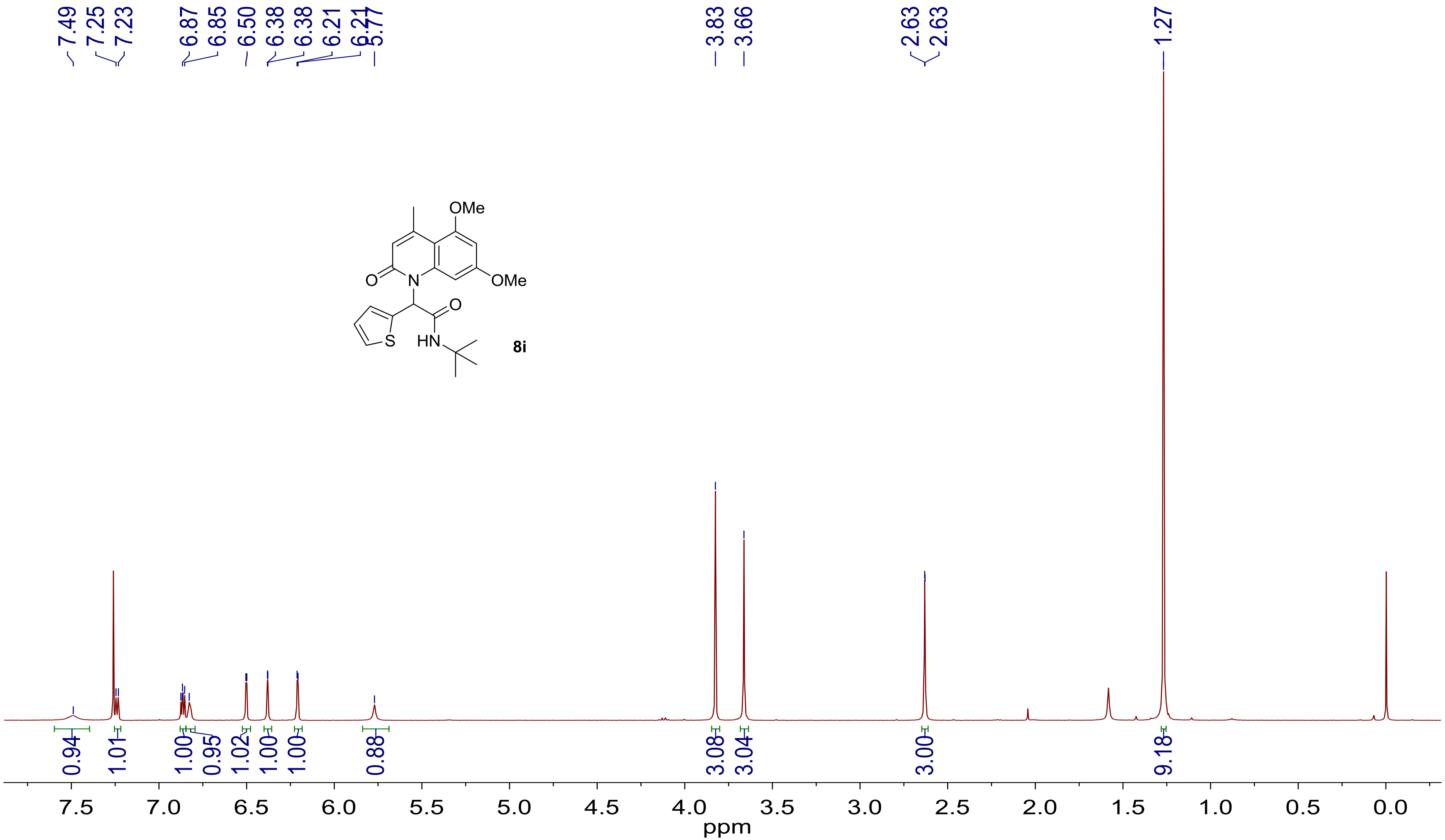
7.49
7.25
7.23
6.87
6.85
6.50
6.38
6.38
6.21
6.21
5.77
3.83
3.66
2.63
2.63
1.27
OMe
OMe
O
N
O
S
HN
8i
0.94
1.01
1.00
0.95
1.02
1.00
1.00
0.88
3.08
3.04
3.00
9.18
7.5
7.0
6.5
6.0
5.5
5.0
4.5
4.0
3.5
3.0
2.5
2.0
1.5
1.0
0.5
0.0
ppm

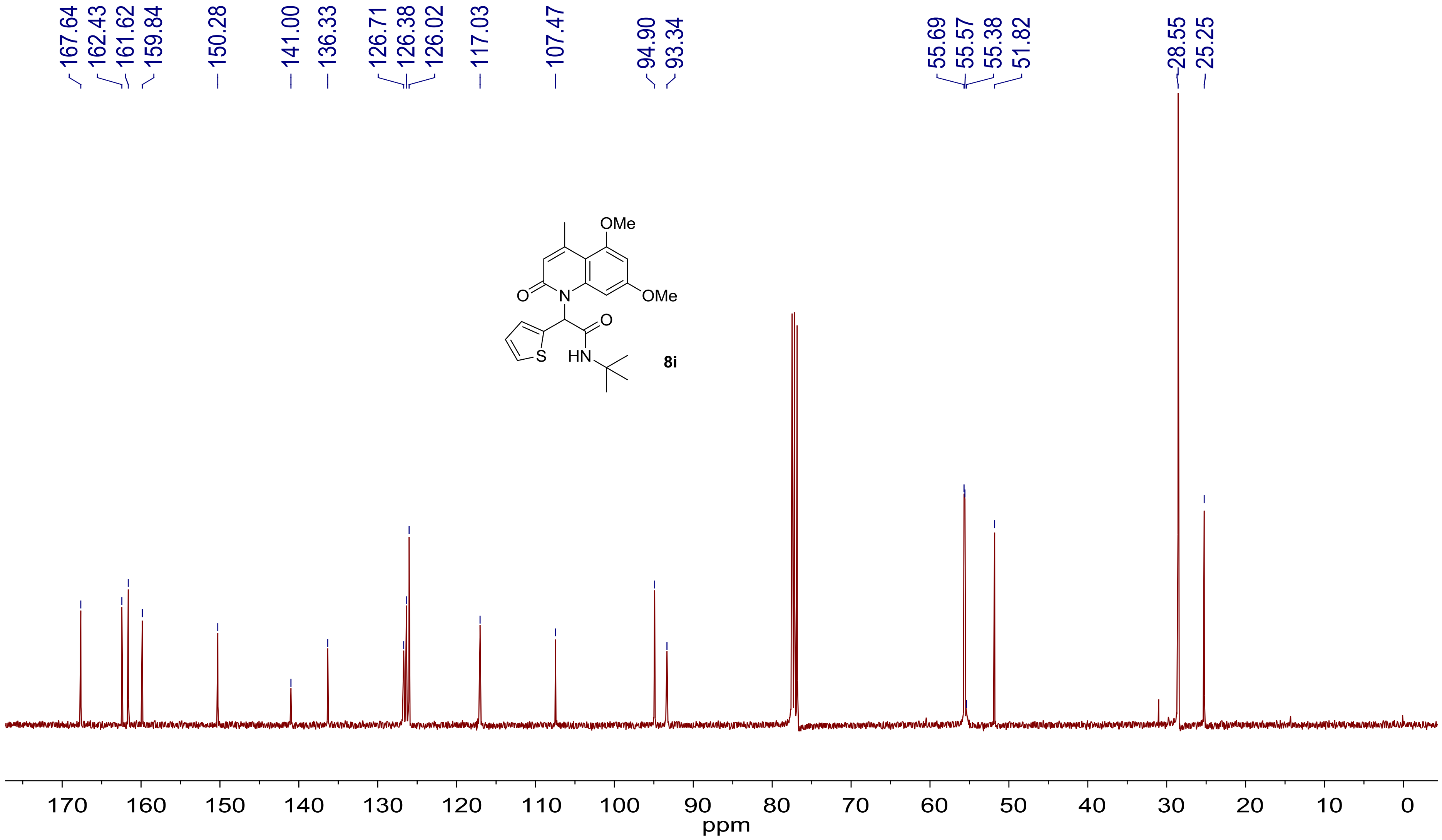
167.64
162.43
161.62
159.84
150.28
141.00
136.33
126.71
126.38
126.02
117.03
107.47
94.90
93.34
55.69
55.57
55.38
51.82
28.55
25.25
OMe
O
N
OMe
O
S
HN
8i
170 160 150 140 130 120 110 100 90 80 70 60 50 40 30 20 10 0
ppm

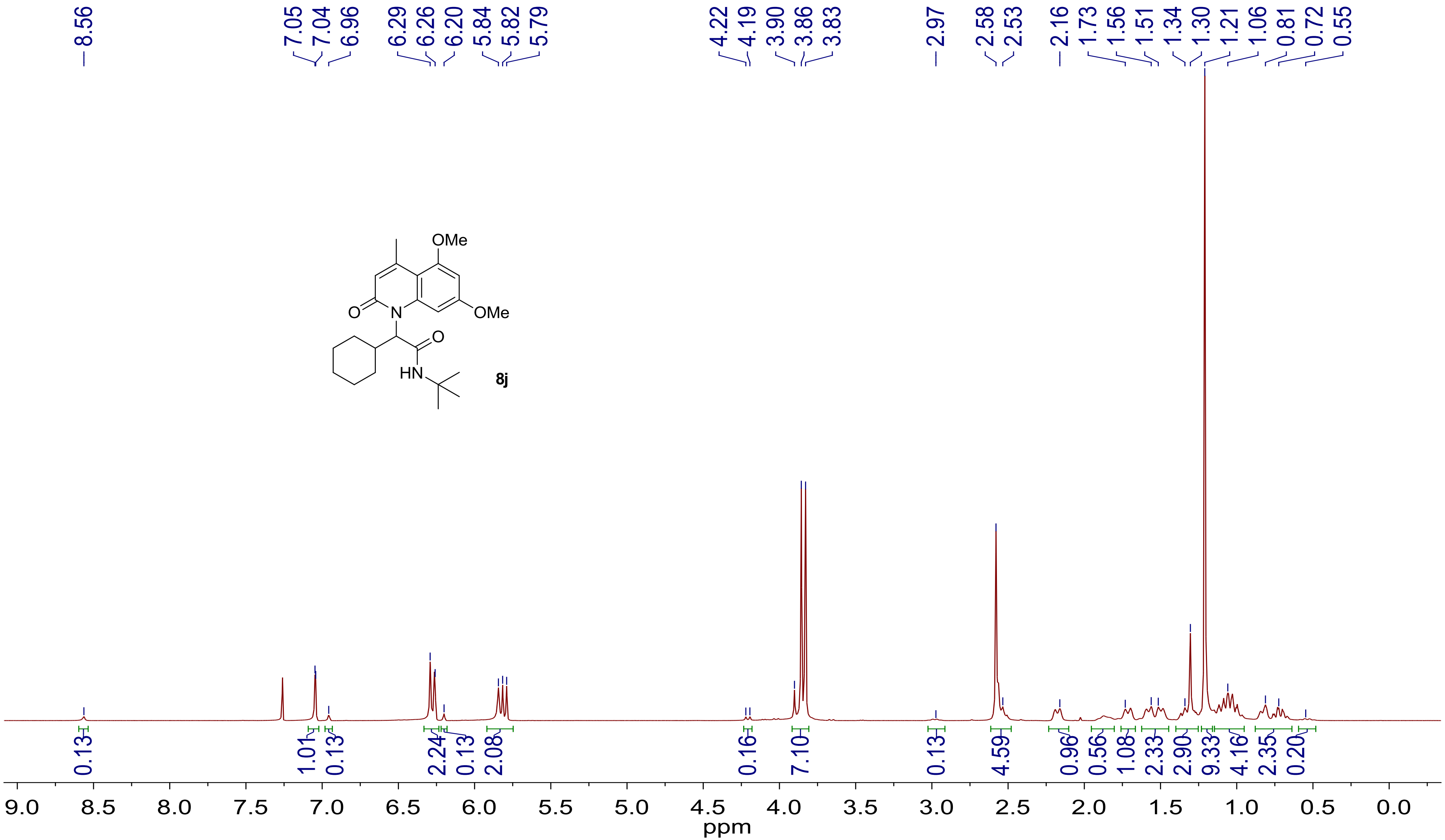

8.56
7.05
7.04
6.96
6.29
6.26
6.20
5.84
5.82
5.79
4.22
4.19
3.90
3.86
3.83
2.97
2.58
2.53
2.16
1.73
1.56
1.51
1.34
1.30
1.21
1.06
0.81
0.72
0.55
OMe
O
N
OMe
O
HN
8j
0.13
1.01
0.13
2.24
0.13
2.08
0.16
7.10
0.13
4.59
0.96
0.56
1.08
2.33
2.90
9.33
4.16
2.35
0.20
9.0
8.5
8.0
7.5
7.0
6.5
6.0
5.5
5.0
4.5
4.0
3.5
3.0
2.5
2.0
1.5
1.0
0.5
0.0
ppm

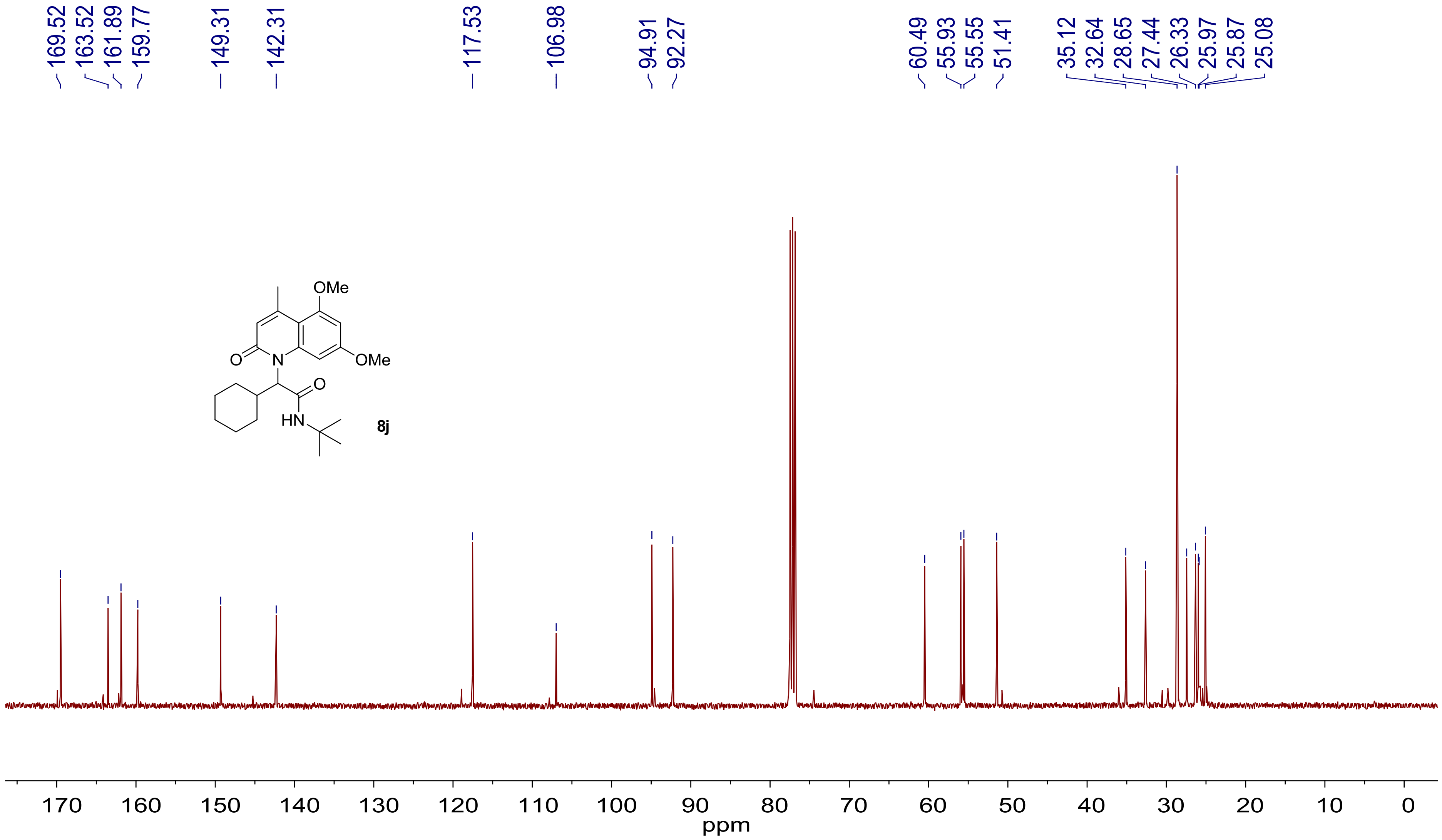
169.52
163.52
161.89
159.77
149.31
142.31
117.53
106.98
94.91
92.27
60.49
55.93
55.55
51.41
35.12
32.64
28.65
27.44
26.33
25.97
25.87
25.08
OMe
OMe
O
N
O
HN
8j
170
160
150
140
130
120
110
100
90
80
70
60
50
40
30
20
10
0
ppm

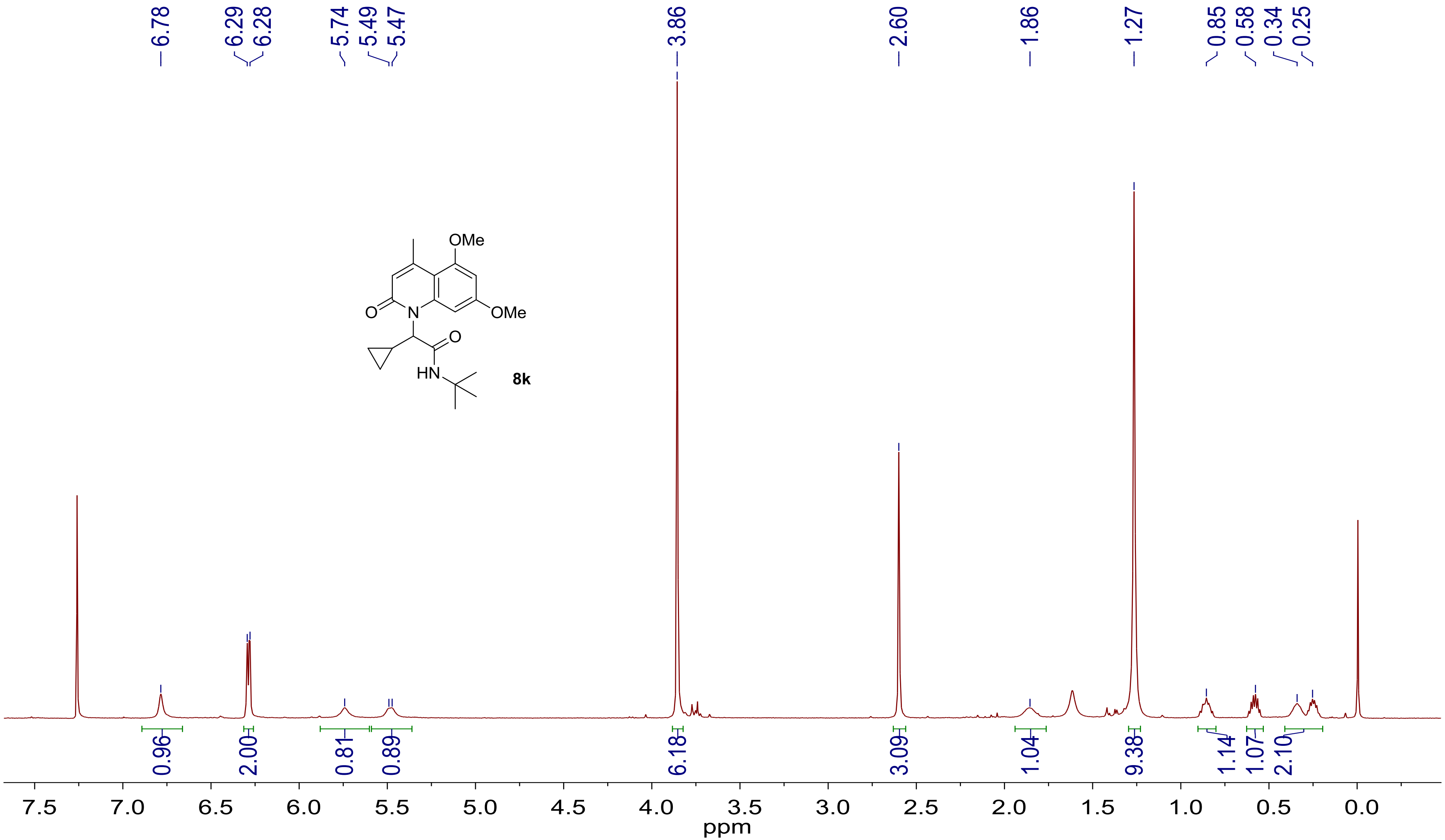
6.78
6.29
6.28
5.74
5.49
5.47
3.86
2.60
1.86
1.27
0.85
0.58
0.34
0.25
OMe
OMe
O
N
O
HN
8k
0.96
2.00
0.81
0.89
6.18
3.09
1.04
9.38
1.14
1.07
2.10
7.5
7.0
6.5
6.0
5.5
5.0
4.5
4.0
3.5
3.0
2.5
2.0
1.5
1.0
0.5
0.0
ppm

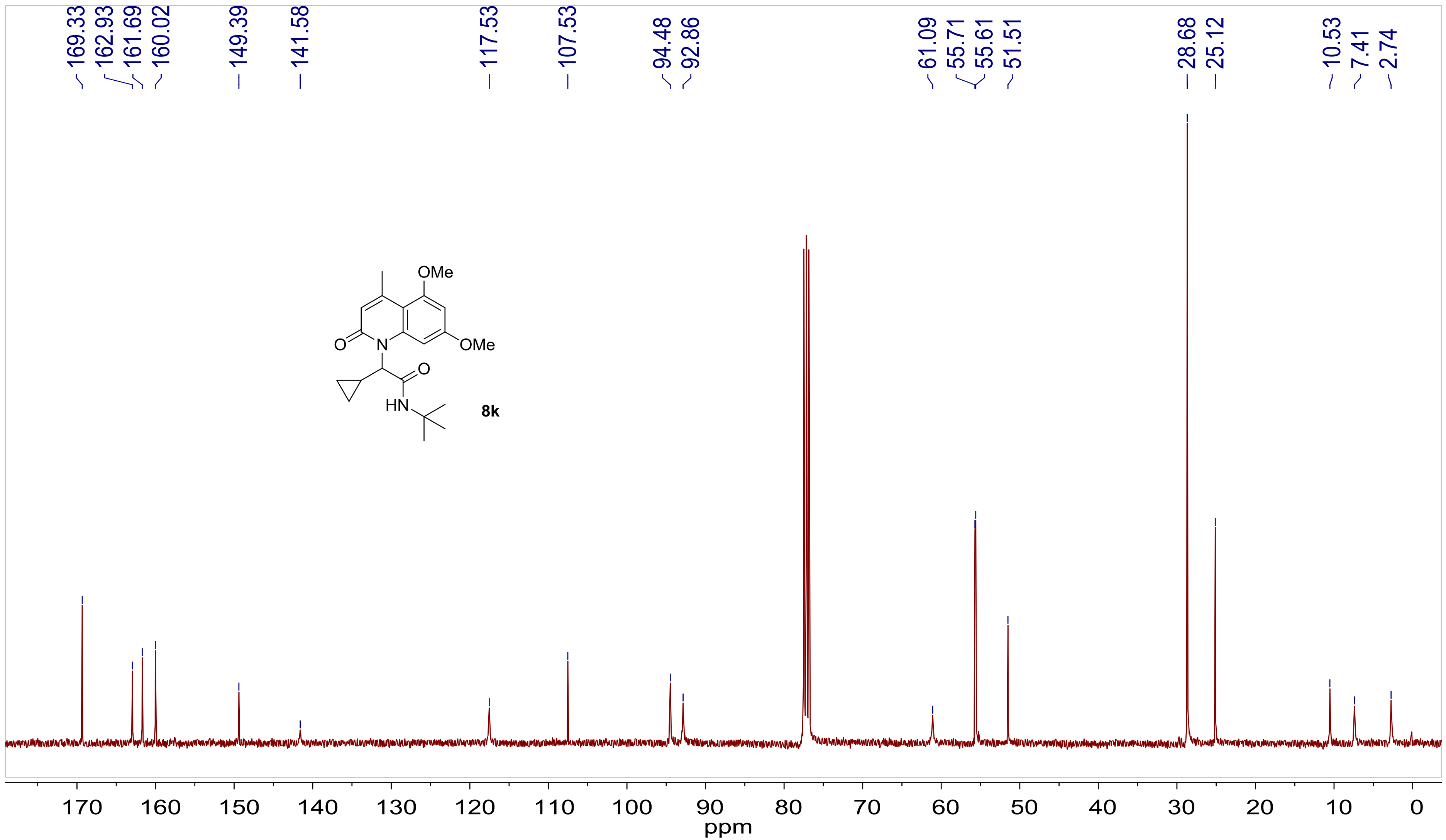
169.33
162.93
161.69
160.02
149.39
141.58
117.53
107.53
94.48
92.86
61.09
55.71
55.61
51.51
28.68
25.12
10.53
7.41
2.74
OMe
OMe
O
N
O
HN
8k
170
160
150
140
130
120
110
100
90
80
70
60
50
40
30
20
10
0
ppm

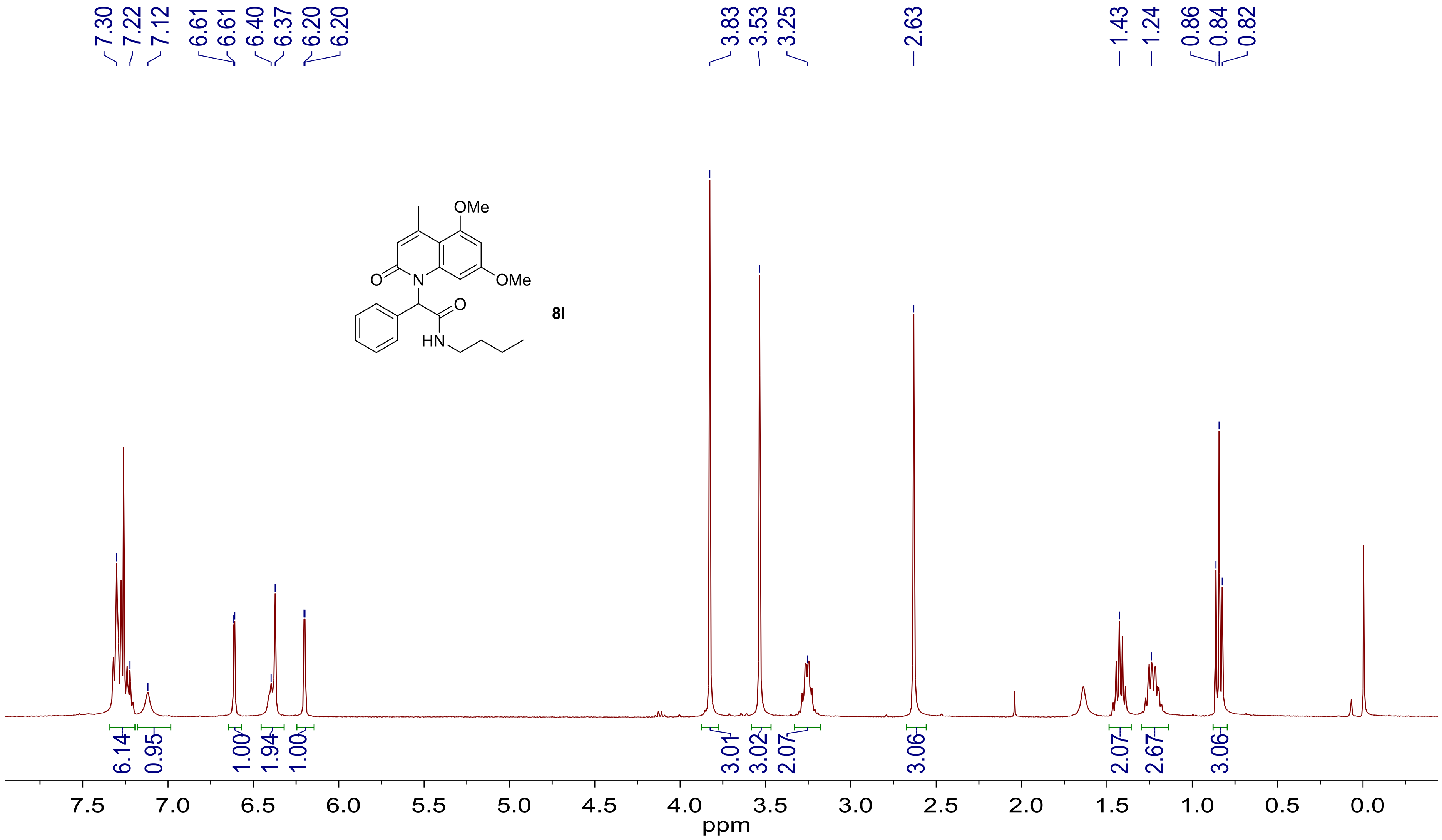
7.30
7.22
7.12
6.61
6.61
6.40
6.37
6.20
6.20
3.83
3.53
3.25
2.63
1.43
1.24
0.86
0.84
0.82
OMe
OMe
O
N
O
HN
8l
6.14
0.95
1.00
1.94
1.00
3.01
3.02
2.07
3.06
2.07
2.67
3.06
7.5
7.0
6.5
6.0
5.5
5.0
4.5
4.0
3.5
3.0
2.5
2.0
1.5
1.0
0.5
0.0
ppm

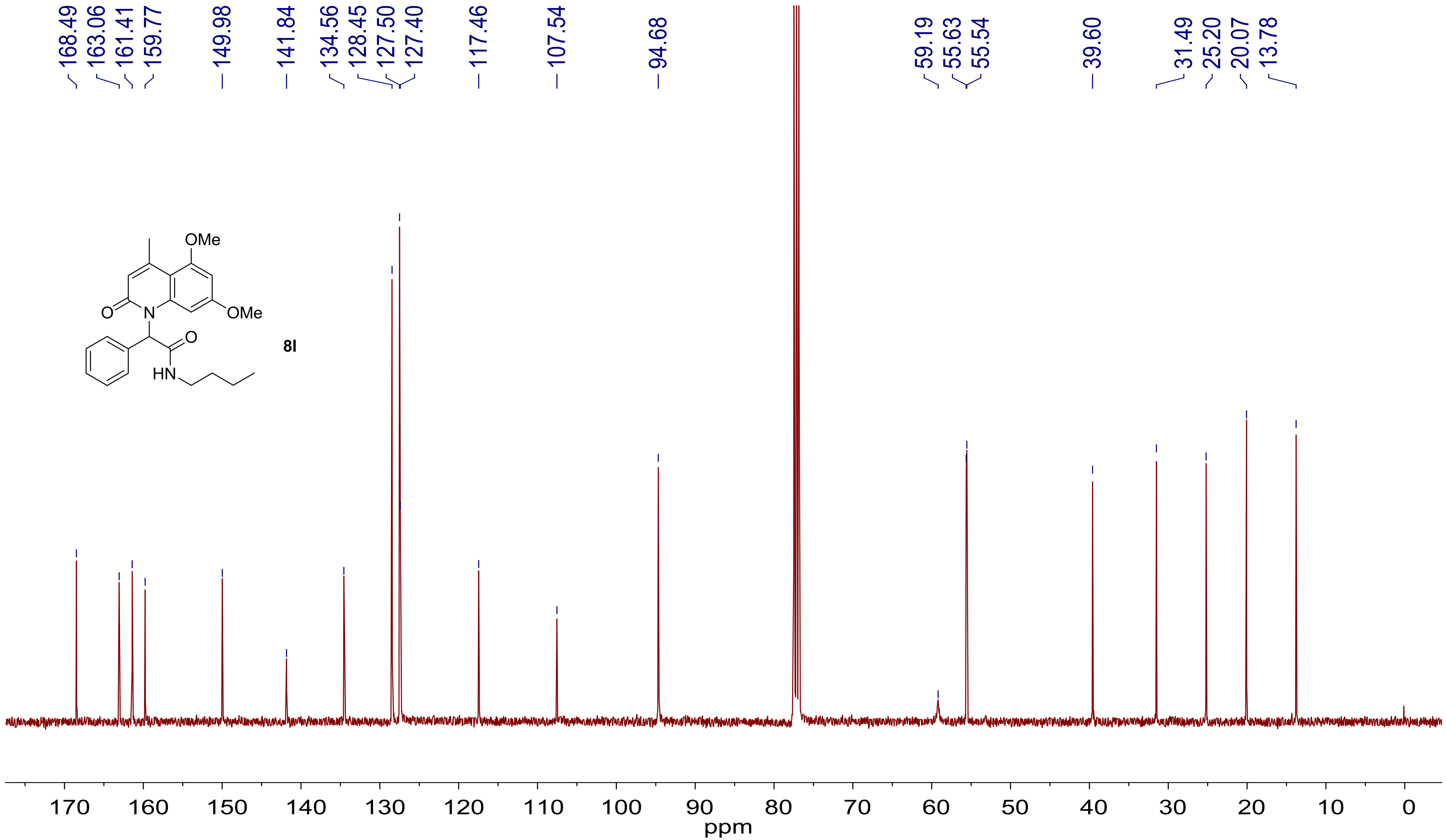
168.49
163.06
161.41
159.77
149.98
141.84
134.56
128.45
127.50
127.40
117.46
107.54
94.68
59.19
55.63
55.54
39.60
31.49
25.20
20.07
13.78
OMe
O
N
OMe
O
HN
8l
170 160 150 140 130 120 110 100 90 80 70 60 50 40 30 20 10 0
ppm

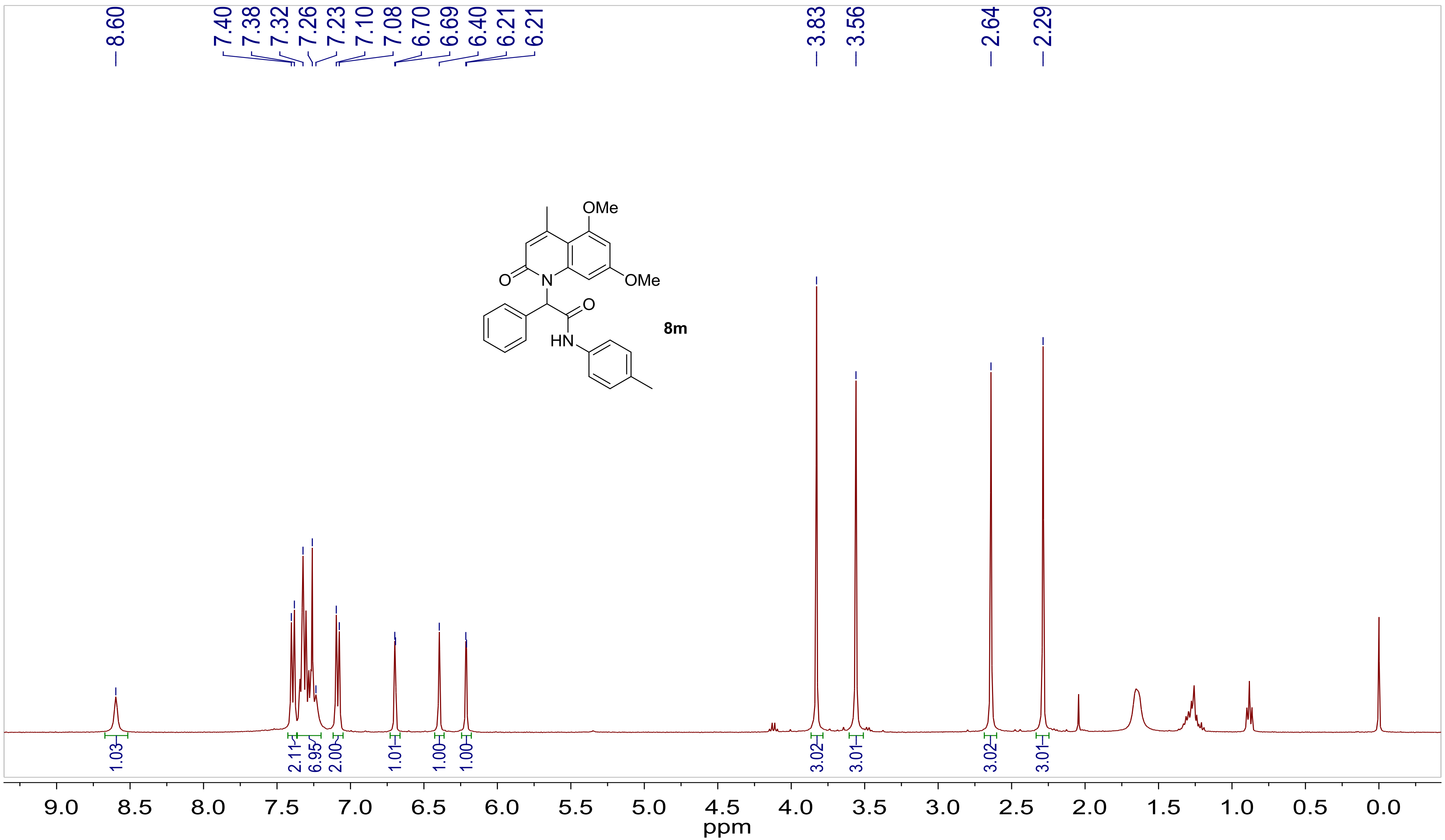
8.60
7.40 7.38 7.32 7.26 7.23 7.10 7.08 6.70 6.69 6.40 6.21 6.21
3.83 3.56 2.64 2.29
OMe
OMe
O
N
O
HN
8m
1.03 2.11 6.95 2.00 1.01 1.00 1.00 3.02 3.01 3.02 3.01
9.0 8.5 8.0 7.5 7.0 6.5 6.0 5.5 5.0 4.5 4.0 3.5 3.0 2.5 2.0 1.5 1.0 0.5 0.0
ppm

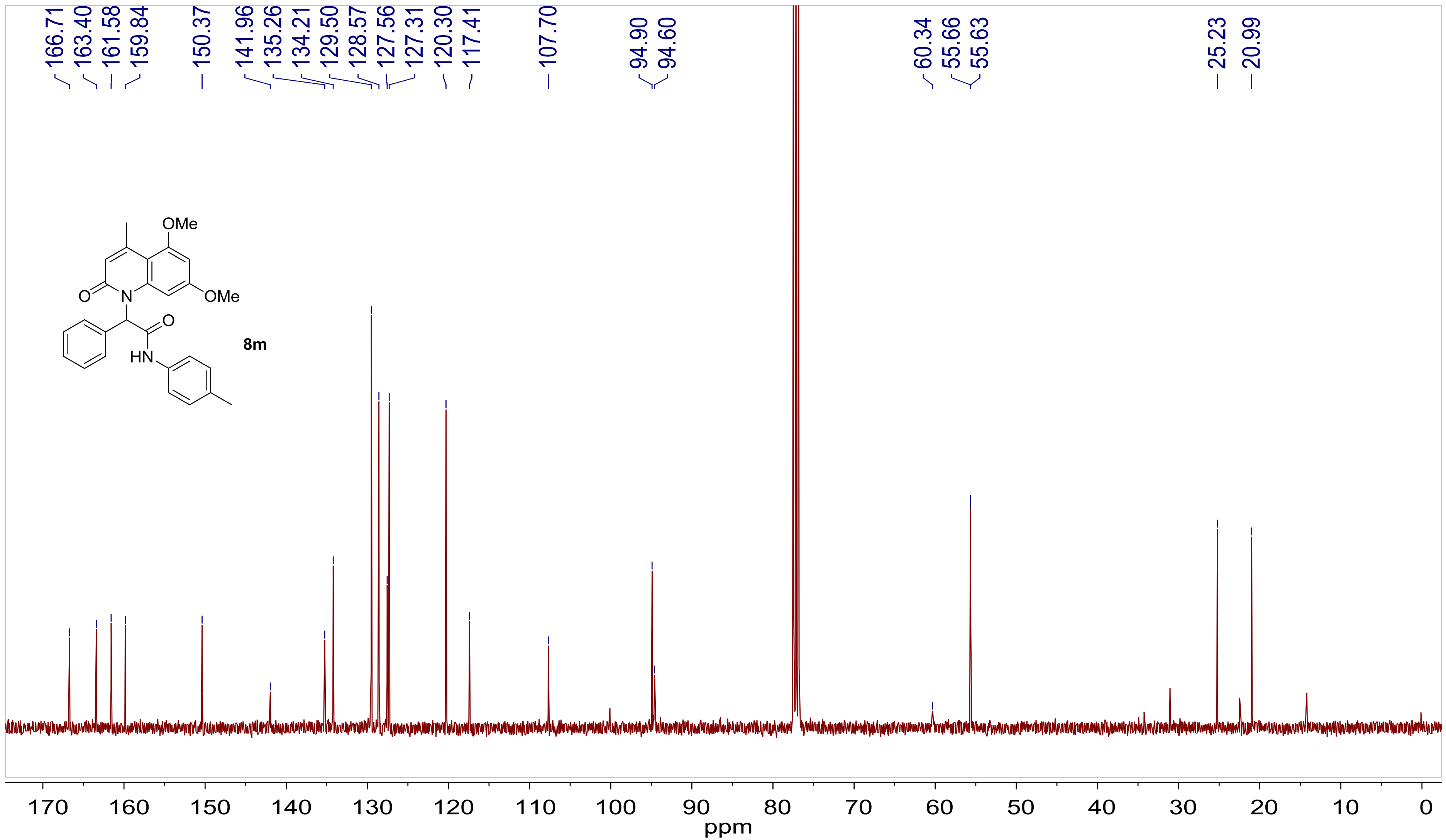
166.71
163.40
161.58
159.84
150.37
141.96
135.26
134.21
129.50
128.57
127.56
127.31
120.30
117.41
107.70
94.90
94.60
60.34
55.66
55.63
25.23
20.99
OMe
OMe
O
N
O
HN
8m
170
160
150
140
130
120
110
100
90
80
70
60
50
40
30
20
10
0
ppm

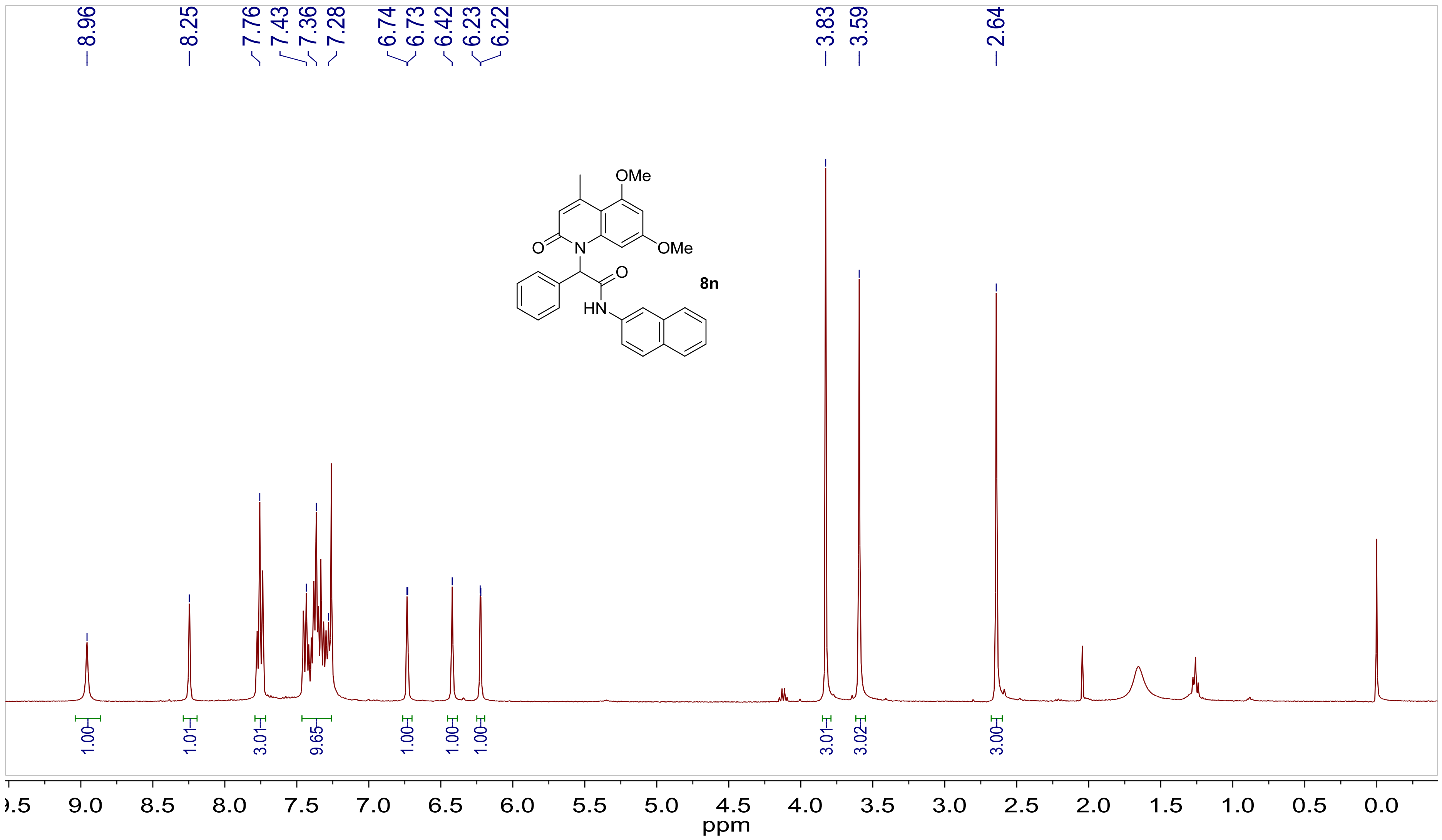
8.96
8.25
7.76
7.43
7.36
7.28
6.74
6.73
6.42
6.23
6.22
3.83
3.59
2.64
OMe
O
N
OMe
O
HN
8n
1.00
1.01
3.01
9.65
1.00
1.00
1.00
3.01
3.02
3.00
9.5
9.0
8.5
8.0
7.5
7.0
6.5
6.0
5.5
5.0
4.5
4.0
3.5
3.0
2.5
2.0
1.5
1.0
0.5
0.0
ppm

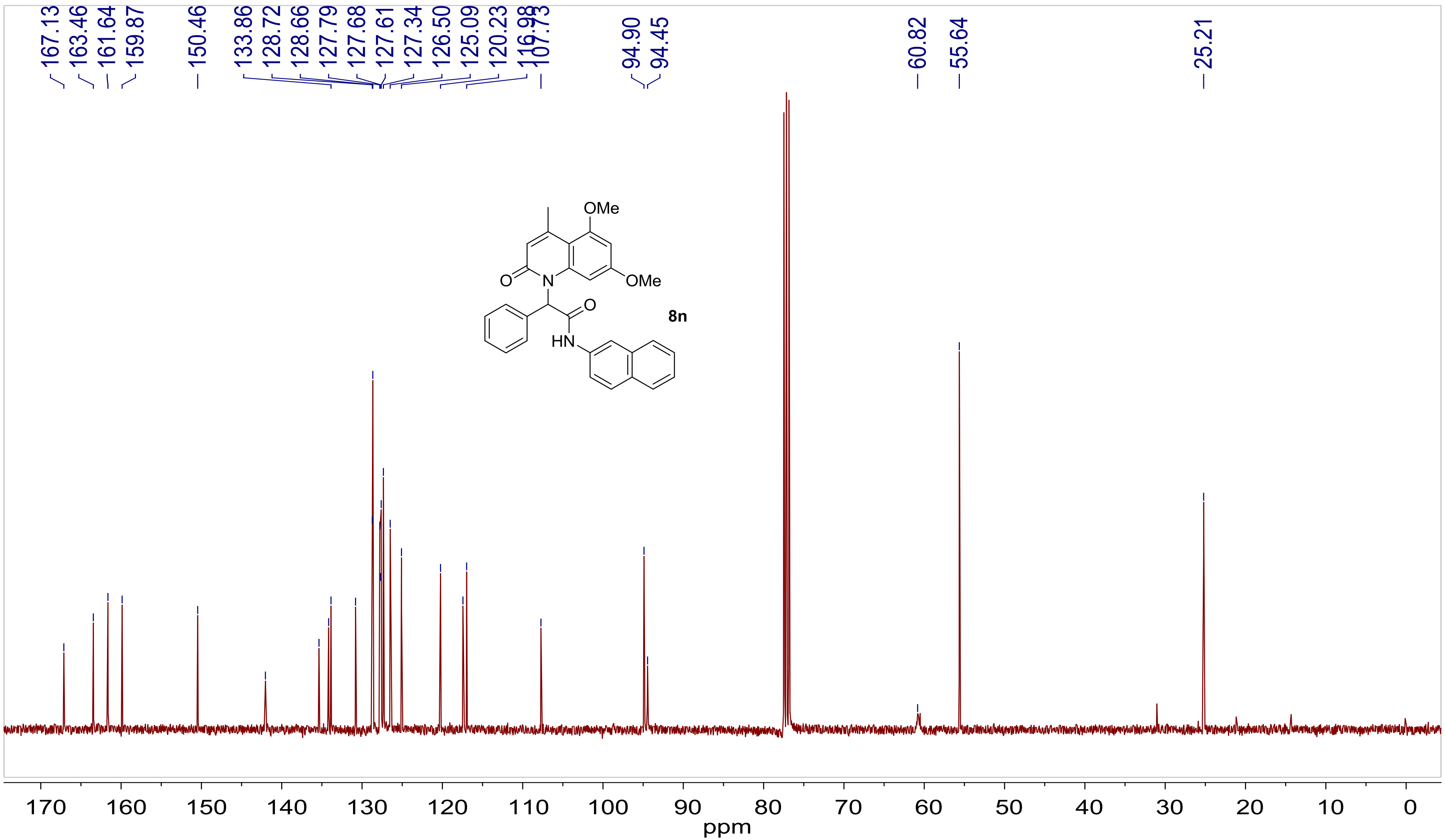

167.13
163.46
161.64
159.87
150.46
133.86
128.72
128.66
127.79
127.68
127.61
127.34
126.50
125.09
120.23
116.98
107.73
94.90
94.45
60.82
55.64
25.21
OMe
OMe
O
N
O
HN
8n
170 160 150 140 130 120 110 100 90 80 70 60 50 40 30 20 10 0
ppm

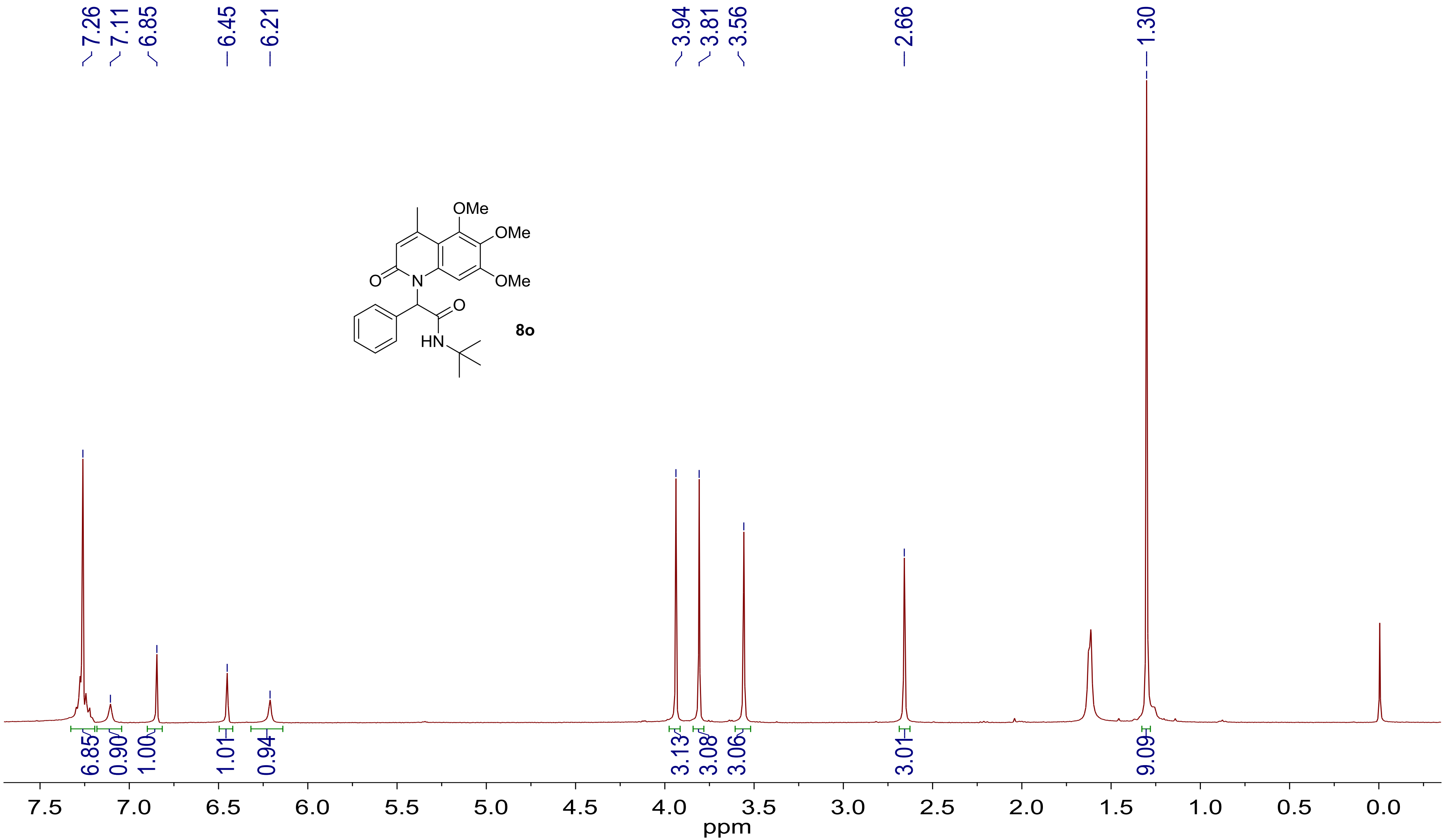
7.26
7.11
6.85
6.45
6.21
3.94
3.81
3.56
2.66
1.30
OMe
OMe
OMe
O
N
O
HN
8o
6.85
0.90
1.00
1.01
0.94
3.13
3.08
3.06
3.01
9.09
7.5 7.0 6.5 6.0 5.5 5.0 4.5 4.0 3.5 3.0 2.5 2.0 1.5 1.0 0.5 0.0
ppm

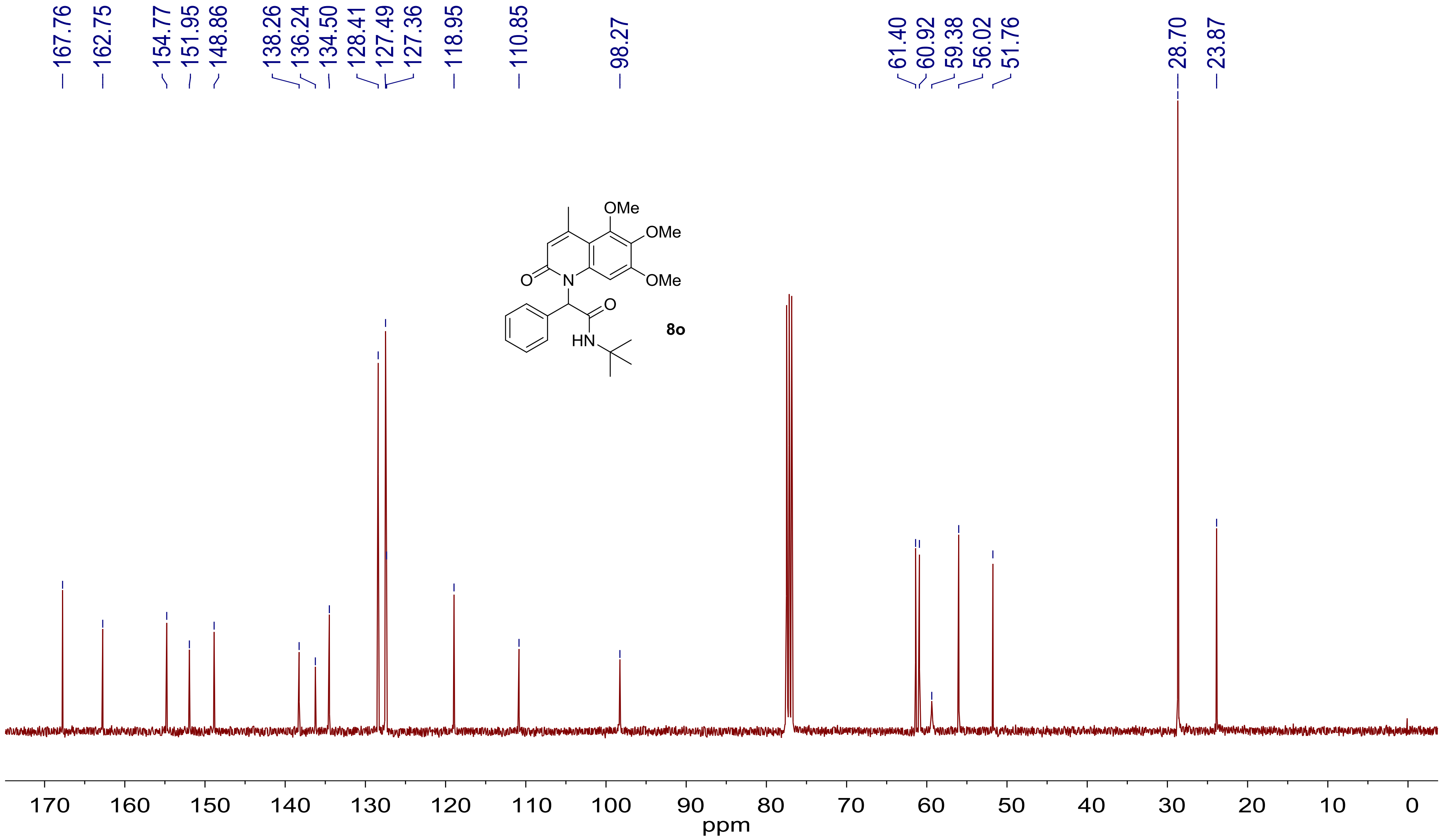
167.76
162.75
154.77
151.95
148.86
138.26
136.24
134.50
128.41
127.49
127.36
118.95
110.85
98.27
61.40
60.92
59.38
56.02
51.76
28.70
23.87
OMe
OMe
OMe
O
N
O
HN
8o
170
160
150
140
130
120
110
100
90
80
70
60
50
40
30
20
10
0
ppm

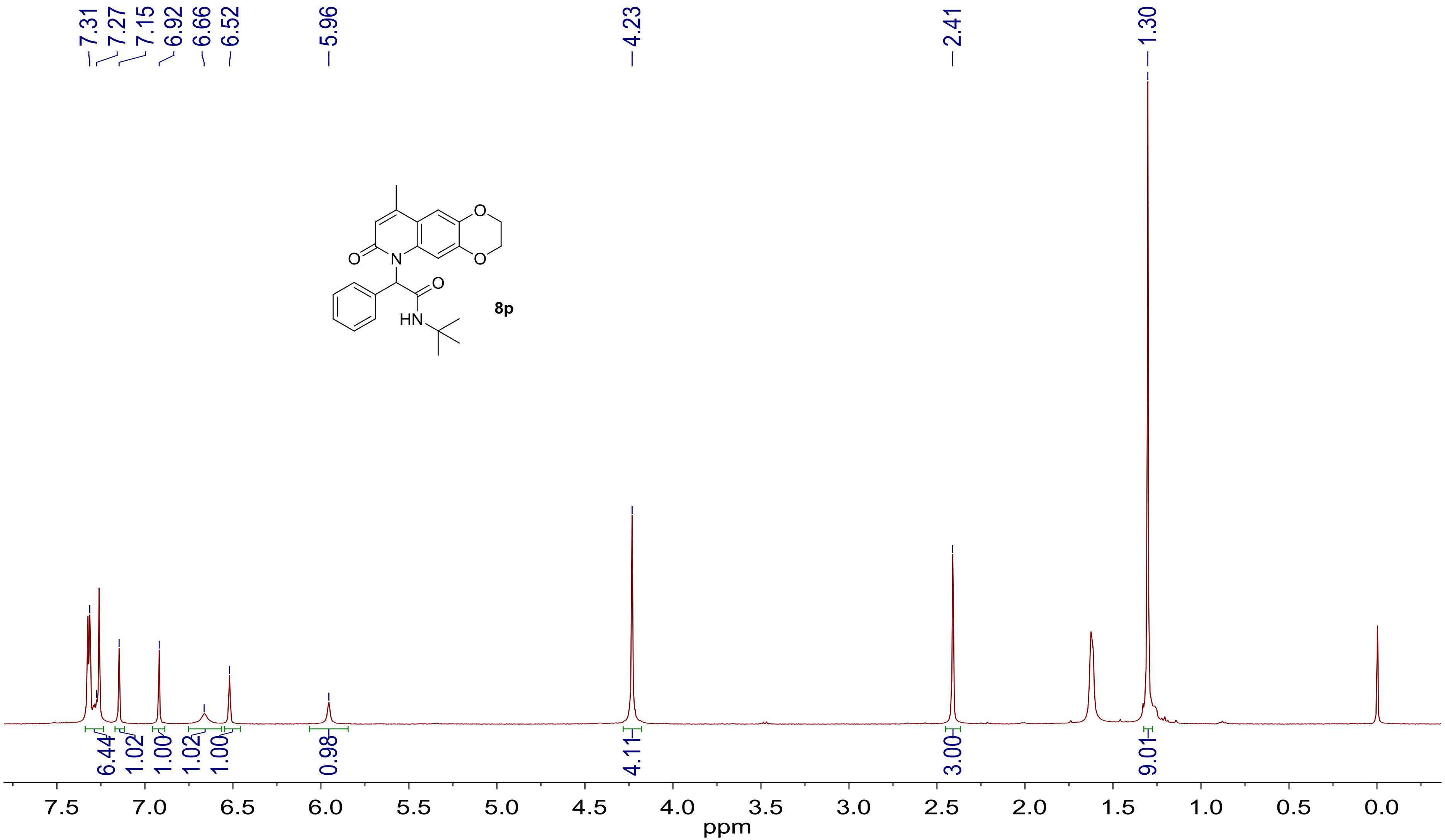
7.31
7.27
7.15
6.92
6.66
6.52
5.96
4.23
2.41
1.30
8p
6.44
1.02
1.00
1.02
1.00
0.98
4.11
3.00
9.01
7.5 7.0 6.5 6.0 5.5 5.0 4.5 4.0 3.5 3.0 2.5 2.0 1.5 1.0 0.5 0.0
ppm

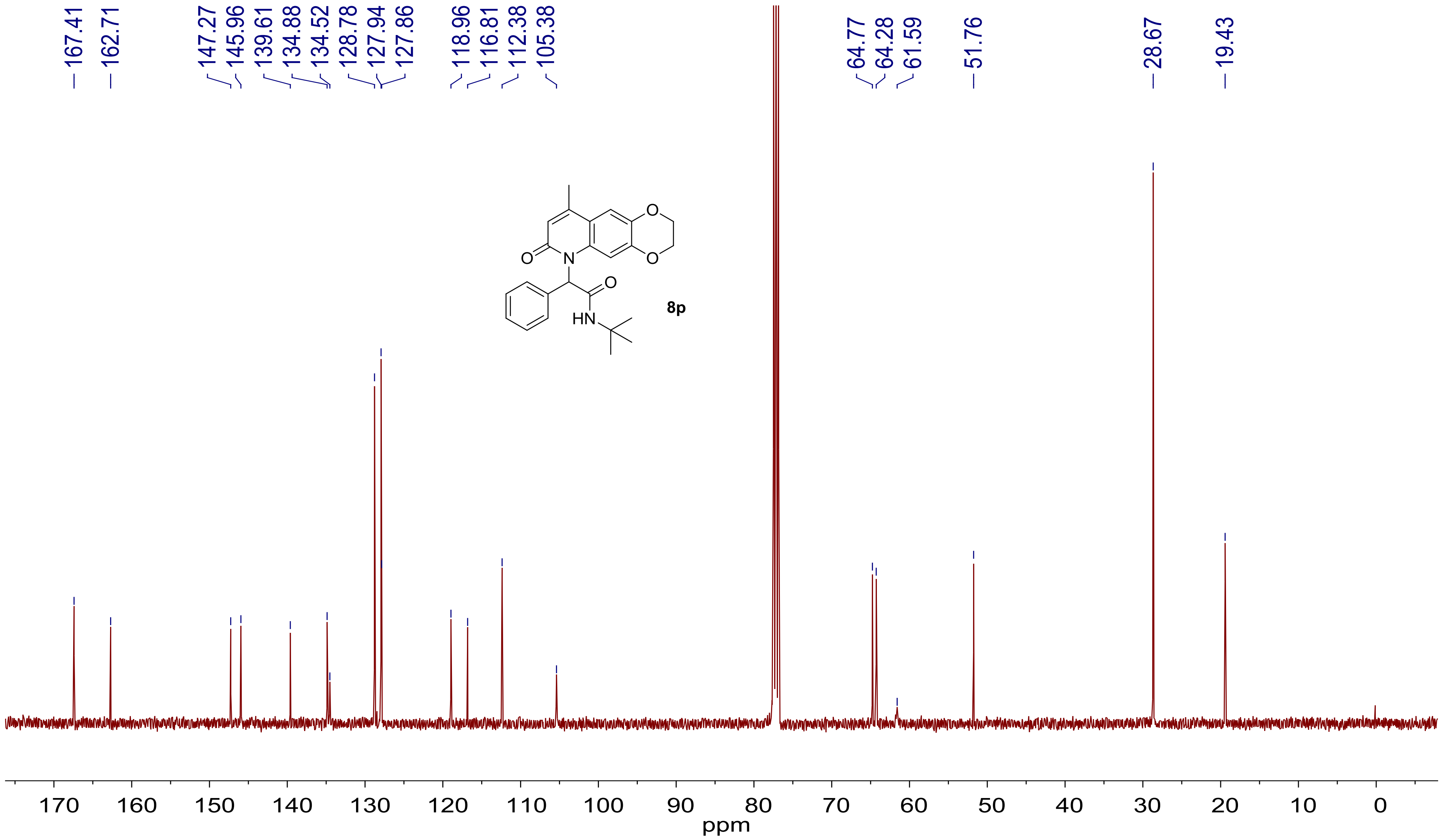
167.41
162.71
147.27
145.96
139.61
134.88
134.52
128.78
127.94
127.86
118.96
116.81
112.38
105.38
64.77
64.28
61.59
51.76
28.67
19.43
O
O
O
N
O
HN
8p
170 160 150 140 130 120 110 100 90 80 70 60 50 40 30 20 10 0
ppm

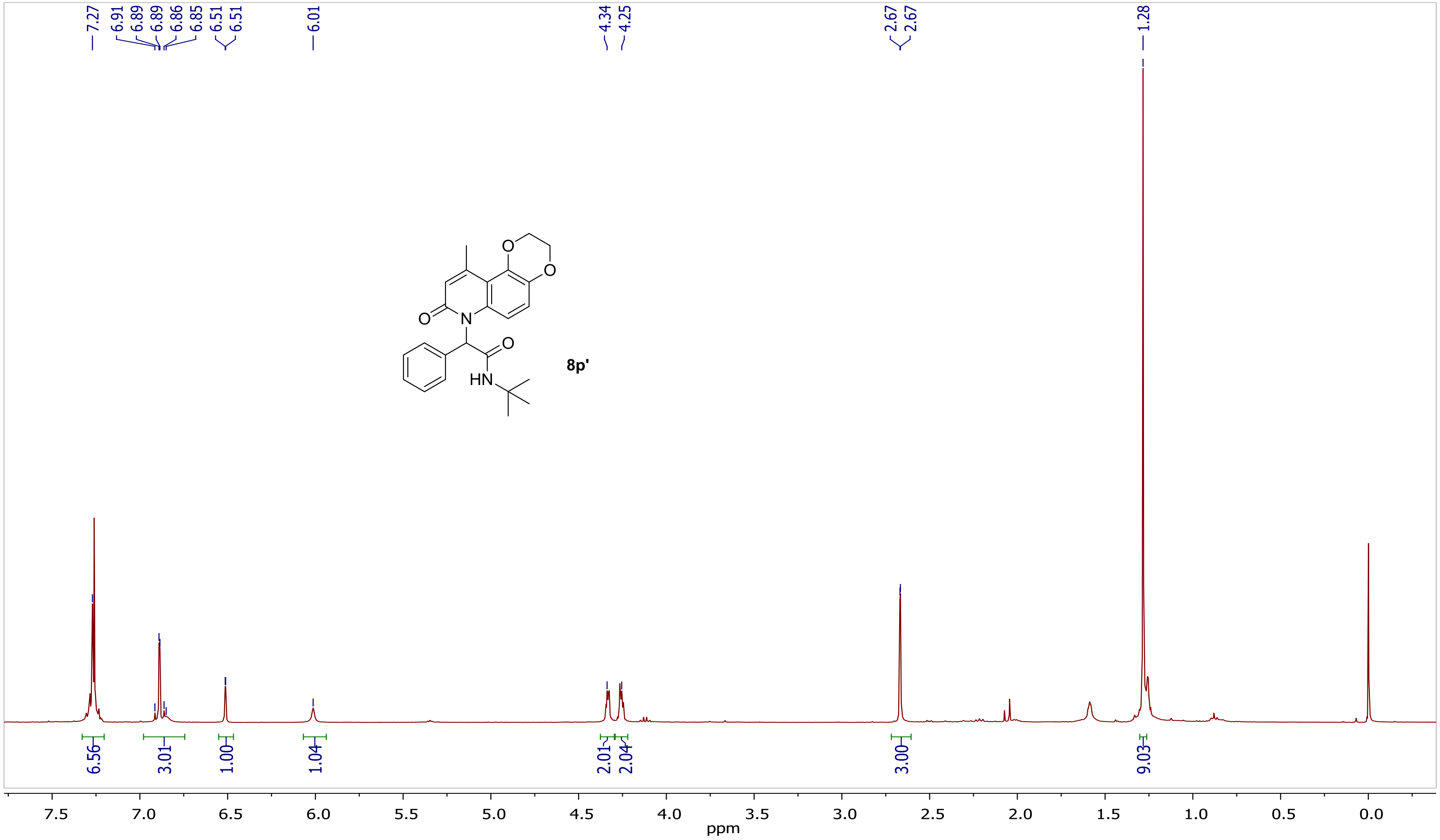
7.27
6.91
6.89
6.89
6.86
6.85
6.51
6.51
6.01
4.34
4.25
2.67
2.67
1.28
O
O
O
N
O
HN
8p'
6.56
3.01
1.00
1.04
2.01
2.04
3.00
9.03
7.5
7.0
6.5
6.0
5.5
5.0
4.5
4.0
3.5
3.0
2.5
2.0
1.5
1.0
0.5
0.0
ppm

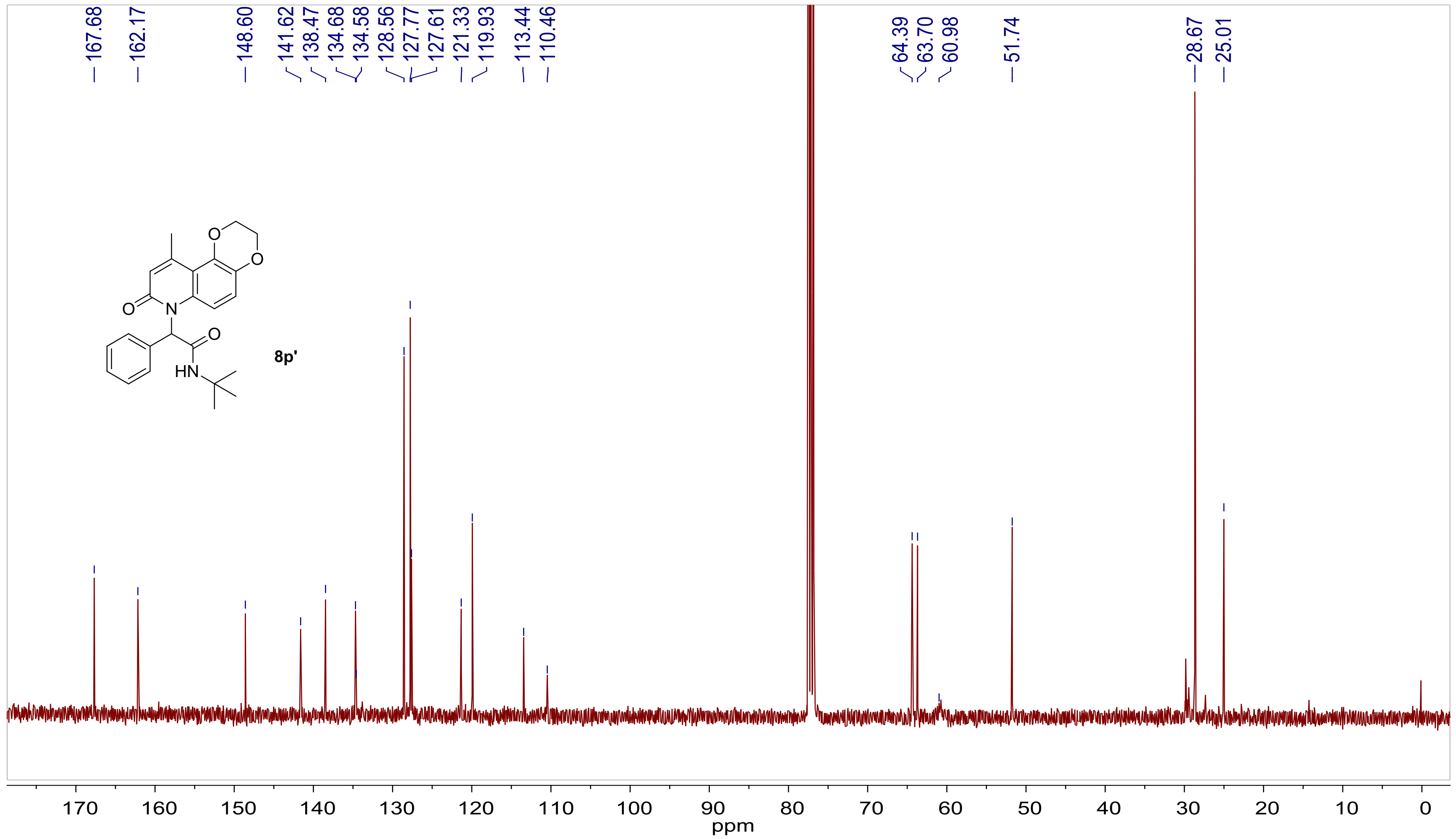

167.68
162.17
148.60
141.62
138.47
134.68
134.58
128.56
127.77
127.61
121.33
119.93
113.44
110.46
64.39
63.70
60.98
51.74
28.67
25.01
O
O
O
N
O
HN
8p'
170
160
150
140
130
120
110
100
90
80
70
60
50
40
30
20
10
0
ppm

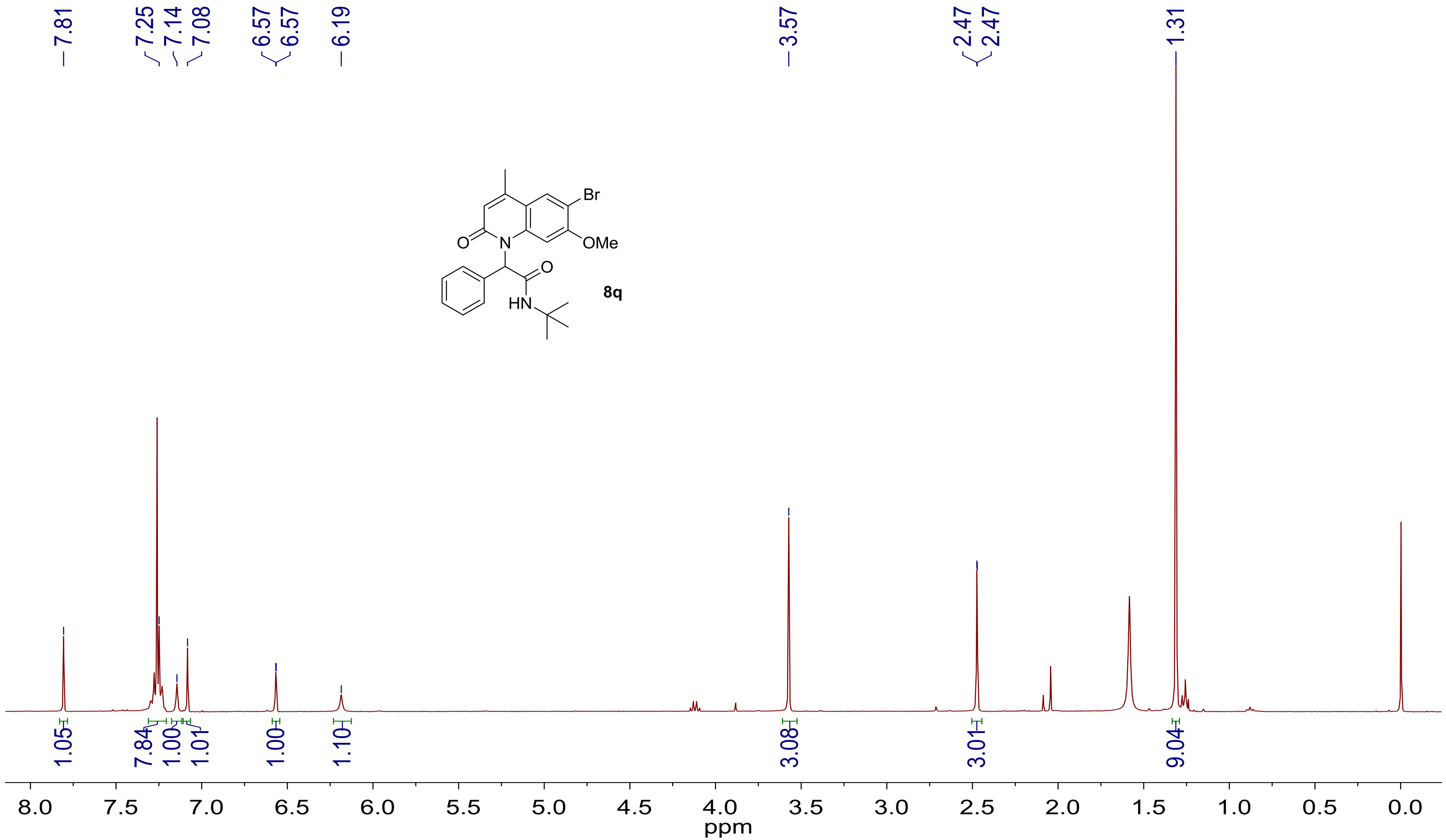
7.81
7.25
7.14
7.08
6.57
6.57
6.19
3.57
2.47
2.47
1.31
Br
O
N
OMe
O
HN
8q
1.05
7.84
1.00
1.01
1.00
1.10
3.08
3.01
9.04
8.0
7.5
7.0
6.5
6.0
5.5
5.0
4.5
4.0
3.5
3.0
2.5
2.0
1.5
1.0
0.5
0.0
ppm

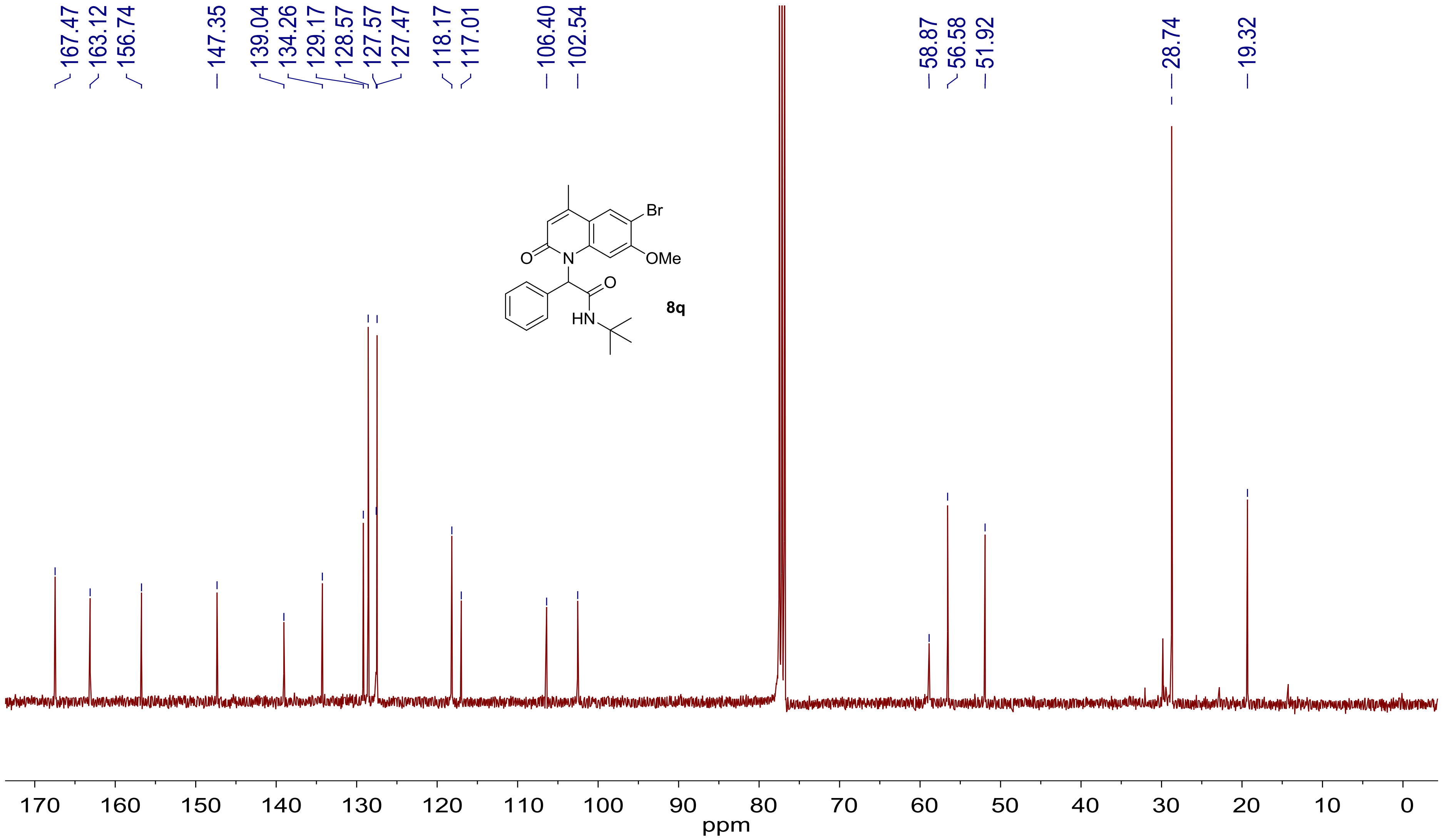
167.47
163.12
156.74
147.35
139.04
134.26
129.17
128.57
127.57
127.47
118.17
117.01
106.40
102.54
58.87
56.58
51.92
28.74
19.32
Br
O
N
OMe
O
HN
8q
170
160
150
140
130
120
110
100
90
80
70
60
50
40
30
20
10
0
ppm

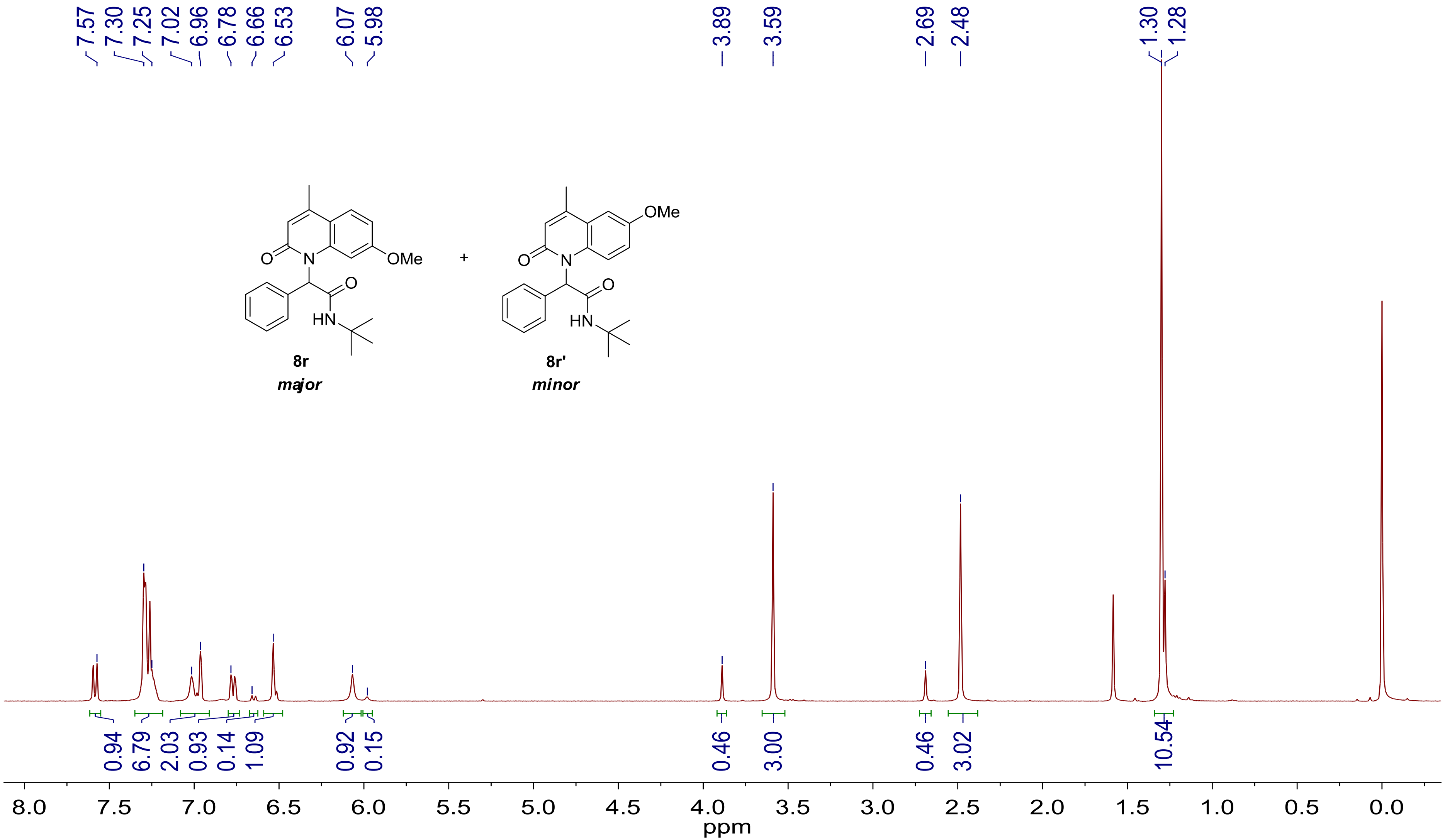
7.57
7.30
7.25
7.02
6.96
6.78
6.66
6.53
6.07
5.98
3.89
3.59
2.69
2.48
1.30
1.28
OMe
O
N
O
HN
8r
major
+
OMe
O
N
O
HN
8r'
minor
0.94
6.79
2.03
0.93
0.14
1.09
0.92
0.15
0.46
3.00
0.46
3.02
10.54
8.0
7.5
7.0
6.5
6.0
5.5
5.0
4.5
4.0
3.5
3.0
2.5
2.0
1.5
1.0
0.5
0.0
ppm

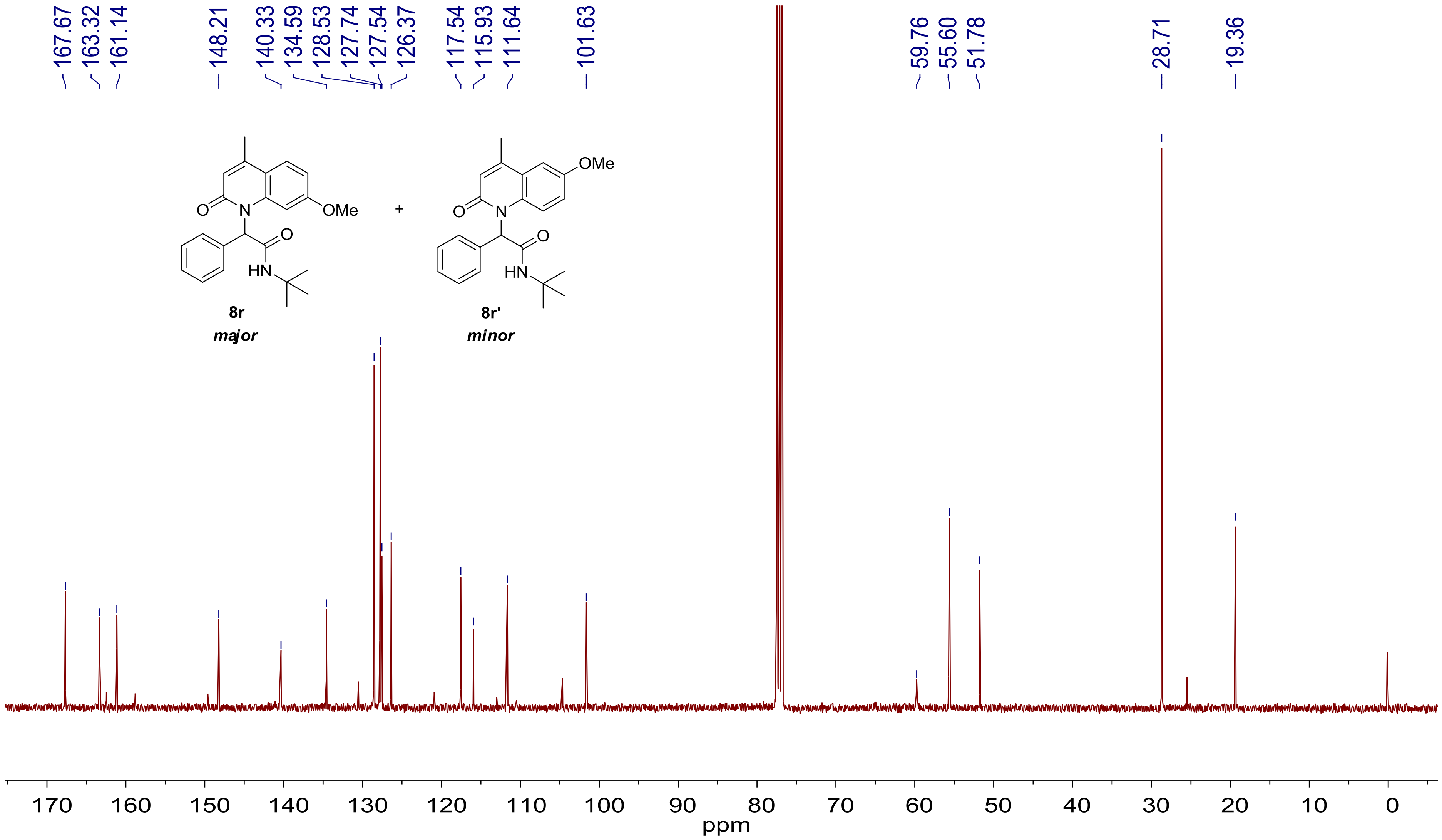
167.67
163.32
161.14
148.21
140.33
134.59
128.53
127.74
127.54
126.37
117.54
115.93
111.64
101.63
59.76
55.60
51.78
28.71
19.36
OMe
O
N
HN
8r
major
+
8r'
minor
170
160
150
140
130
120
110
100
90
80
70
60
50
40
30
20
10
0
ppm

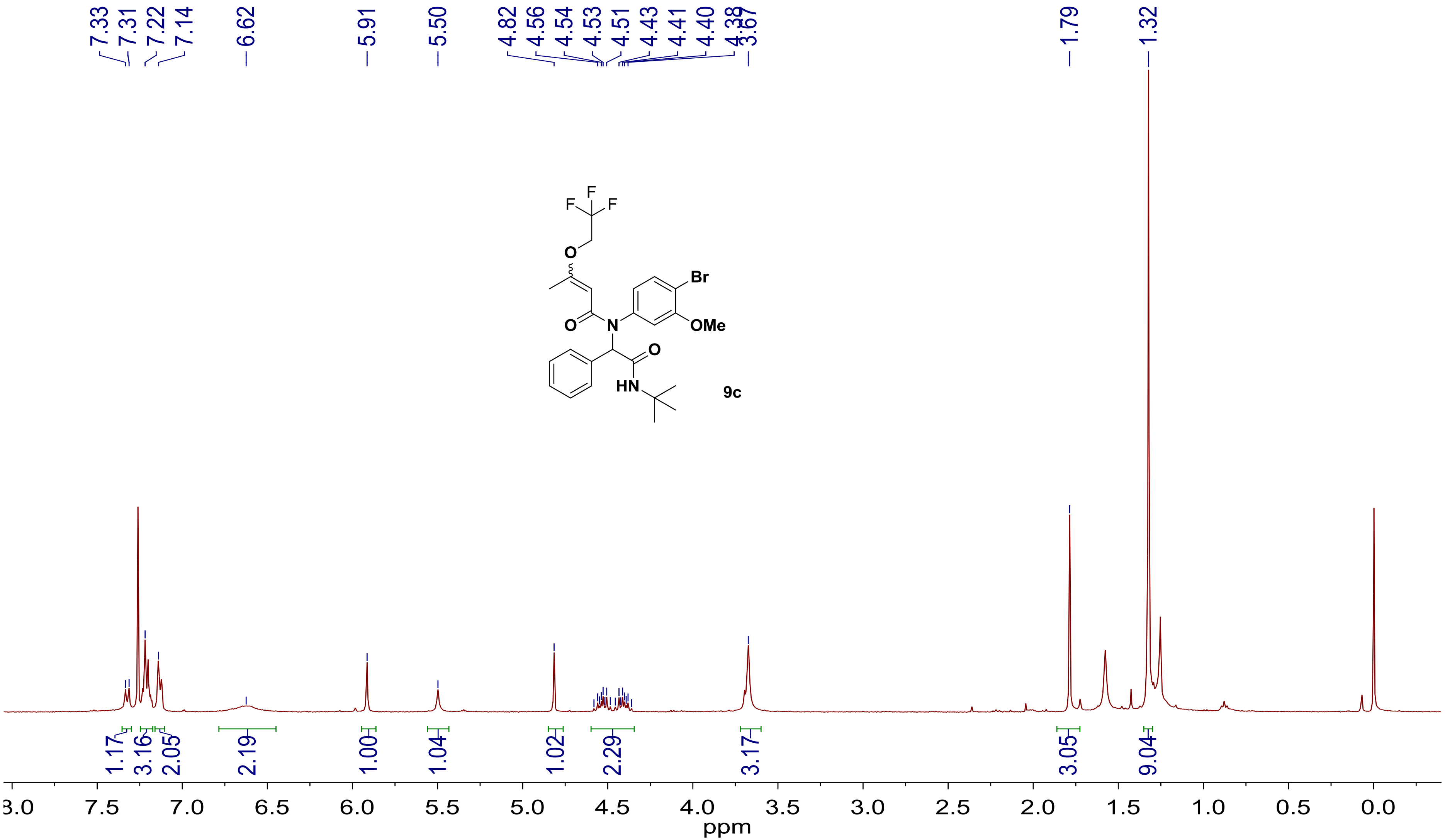
7.33
7.31
7.22
7.14
6.62
5.91
5.50
4.82
4.56
4.54
4.53
4.51
4.43
4.41
4.40
4.38
3.67
1.79
1.32
F
F
F
O
Br
O
N
OMe
O
HN
9c
1.17
3.16
2.05
2.19
1.00
1.04
1.02
2.29
3.17
3.05
9.04
8.0
7.5
7.0
6.5
6.0
5.5
5.0
4.5
4.0
3.5
3.0
2.5
2.0
1.5
1.0
0.5
0.0
ppm

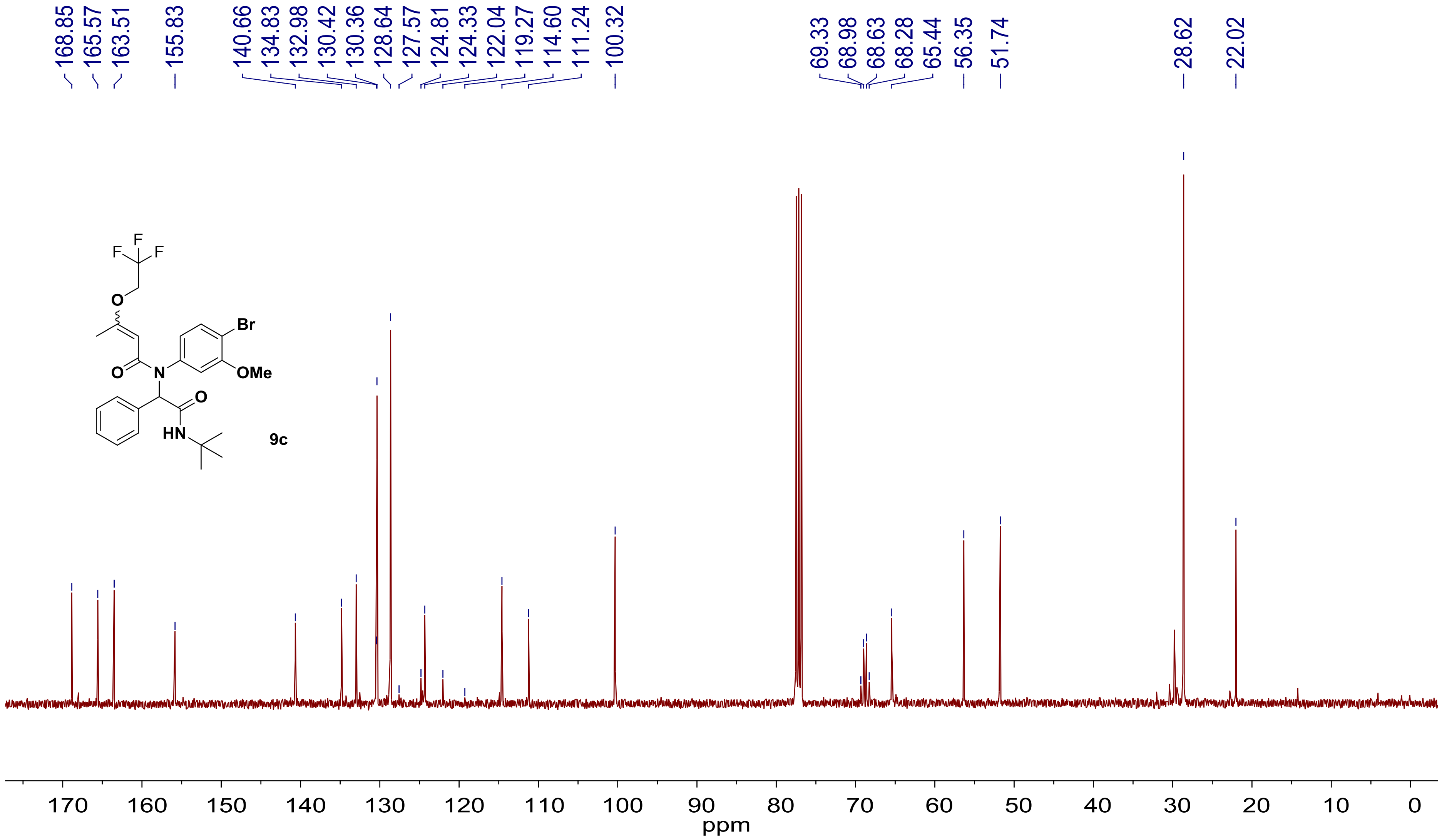
168.85
165.57
163.51
155.83
140.66
134.83
132.98
130.42
130.36
128.64
127.57
124.81
124.33
122.04
119.27
114.60
111.24
100.32
69.33
68.98
68.63
68.28
65.44
56.35
51.74
28.62
22.02
F
F
F
O
Br
O
N
OMe
O
HN
9c
170 160 150 140 130 120 110 100 90 80 70 60 50 40 30 20 10 0
ppm
